# The presence of residual gold nanoparticles in samples interferes with the RT-qPCR assay used for gene expression profiling

**DOI:** 10.1186/s12951-017-0299-9

**Published:** 2017-10-10

**Authors:** Natasha M. Sanabria, Mary Gulumian

**Affiliations:** 1 National Institute for Occupational Health, Johannesburg, South Africa; 2 Haematology and Molecular Medicine Department, School of Pathology, University of the Witwatersrand, Johannesburg, South Africa

**Keywords:** RT-qPCR, AuNPS, Nanoparticles, Engineered nanomaterials, Toxicology, Risk assessment

## Abstract

**Background:**

RT-qPCR is routinely used in expression profiling of toxicity pathway genes. However, genetic and molecular level studies used to determine, understand and clarify potential risks of engineered nanomaterials (ENMs) are still incomplete. Concerns regarding possible interference caused by intracellular ENMs during analyses have been raised. The aim of this study was to verify a qPCR procedure for gene expression assays, which can be used in toxicity and exposure assessments.

**Results:**

Amplification of ten reference genes was performed to test the expression stability. A preliminary study was performed on RNA from BEAS-2B cells that had been treated with AuNPs. Also, a reference total RNA standard from ten cell lines was spiked with various amounts of the same AuNP. This treatment mimics exposure assessment studies, where assay-interference may be caused by intracellular residual ENMs still being present in the biological samples (during and after isolation/purification procedures). Both types of RNA samples were reverse transcribed and then amplified by qPCR. The qPCR-related software and statistical programs used included BestKeeper, NormFinder, REST and qBase+. These results proved that using standard qPCR analysis and statistical programs should not be the only procedure applied to verify the assay for gene expression assessment related to ENMs. A comparison of SYBR Green to EVA Green was discussed, in addition to a comparison to the latest reports regarding the influence of ENM thermal conductivity, surface interactions with ENMs, effects of ENM size and charge, as well as, the limit of detection in a qPCR assay.

**Conclusions:**

AuNPs have the potential to interfere with the assay mechanism of RT-qPCR, thus, assay verification is required for AuNP-related gene expression studies used to evaluate toxicity. It is recommended to use *HSP90* and *YWHAZ* as reference genes, i.e. these were the most stable in our study, irrespective of the source of the RNA, or, the point at which the AuNPs interacted with the assay. This report describes steps that can be utilised to generate a suitable method for gene expression studies associated with toxicity testing of various ENMs. For example, RNA standards that have been spiked with known amounts of ENMs should be run in conjunction with the unknown samples, in order to verify any RT-qPCR assay and determine the degree of error.

**Electronic supplementary material:**

The online version of this article (doi:10.1186/s12951-017-0299-9) contains supplementary material, which is available to authorized users.

## Background

Engineered nanomaterials (ENMs) are defined as materials with at least one dimension smaller than 100 nm. Since ENMs have unique surface characteristics, they have become popular in consumer- and medical-based industries. However, there is a growing concern regarding the related toxicity. Therefore, the risk of exposure has increased and the possible toxicity of these ENMs must be determined. In addition, it has become increasingly important to validate assay parameters for techniques used to determine cyto- and genotoxicity. Overall, there is a lack of assay validation when conducting research with ENMs, especially with regard to routine tests for nucleic acid quantification [1]. Therefore, gene expression assays that rely heavily on RNA with excellent quality should be undertaken with great care, since it has already been shown that AuNPs could interfere with traditional RNA analyses [1].

The most common method for studying gene expression is “real-time” reverse transcription quantitative polymerase chain reaction (RT-qPCR). It is a highly sensitive technique that requires normalisation of the expression data between samples. Although different normalization strategies are available, the most common approach is to use reference genes as internal controls [2–4]. Reference genes are used to compensate for differences in the amount of starting material, efficiency of amplification, as well as, differences in expression between cells and the overall level of transcription [5, 6]. Theoretically, the perfect reference gene would be stably expressed irrespective of the cell type or experimental conditions. However, this is not physically possible since there is no universal reference gene that is found in all cells and could be stably expressed under all experimental conditions.

The identification of stable reference genes, especially for normalisation in RT-qPCR studies employed to assess AuNPs, is essential. Although many different effects of AuNPs in PCR have been published, it is not being applied to the field of toxicology or nano-toxicology studies, i.e. where data can be misinterpreted as being non/toxic simply due to assay interference. Possibilities exist that ENMs, specifically AuNPs, may interfere with the binding of the reverse transcriptase enzyme to the template RNA strand and subsequent transcription. In addition, AuNPs have the potential to influence the *Taq* enzyme binding to the cDNA, as well as, the dyes in the PCR cocktail. Consequently, the unique properties of ENMs may result in expression variation of the reference genes between different samples and/or under different treatment conditions. Hence, variation of the reference genes would significantly affect the analysis of expression alterations of the actual genes-of-interest or targets [4, 7]. Therefore, the identification and use of appropriate and reliable reference genes for normalization is of fundamental importance in gene expression experiments used to analyse new groups of materials (e.g. ENMs). Candidates for reference genes to be tested for AuNP or ENM-related studies can be identified from literature. Although not all the reports may be specific to ENMs, the reports are still able to identify reliable genes from human cell lines under other various experimental conditions [8, 9]. The genes identified in this manner include Human 18S ribosomal RNA (*18S*), beta-actin (*ACTB*), glyceraldehyde-3-phosphate dehydrogenase (*GAPDH*), beta glucuronidase (*GUSB*), Heat shock protein 90 kDa alpha (cytosolic), class B (*HSP90*), hypoxanthine phosphoribosyltransferase 1 (*HPRT1*), peptidylprolyl isomerase A (cyclophilin A) (*PPI*), succinate dehydrogenase complex subunit A flavoprotein (*SDH*), TATA-box binding protein (*TBP*), and Tyrosine 3-monooxygenase/tryptophan 5-monooxygenase activation protein zeta polypeptide (*YWHAZ*).

Numerous methods and algorithms have been developed to select for stable reference genes, where the most common are BestKeeper, NormFinder, REST and qBase+. The BestKeeper method is an Excel-based software tool that makes use of pair-wise correlations in order to determine the most stable reference gene [10]. It uses all the raw quantitative cycle (C_q_) values, from which the geometric mean is calculated to generate the BestKeeper index. Thereafter, Pearson correlations can be calculated between each individual gene and the index. This is then reported as the BestKeeper correlation coefficient. The highest BestKeeper coefficient indicates the most stable expressed gene. Although the analyses are performed in the background, this method still functions even if some data is missing, since the calculations remain visible to the user [10, 11]. NormFinder relies on an input “Q” value that is derived from the C_q_, relative to the PCR efficiency [12, 13]. This program log transforms the data, followed by analysis of variance. The deviation of the measured value, compared to the expected value, is used to calculate a stability value. This stability value is then used to rank the genes, where the lowest stability value indicates the most stable gene expression [11]. The qBase+ software is based on a combination of the proven geNorm technology, as well as, qbase. Specifically, the qBase software is based on models for relative quantification and inter-run calibration, which also includes proper error propagation throughout the calculation track [14]. On the other hand, the geNorm program uses normalised Cq values, where the Cq of a particular gene is first normalised to the sample with the highest expression for that gene, i.e. the minimum Cq value. Pair-wise comparisons are performed for each gene, with every other gene, in order to determine their relative stability (M) [5, 15]. The lower M values, therefore, represent genes with more stable expression across the samples being analysed. This method is simpler and more user-friendly, but the equations are hidden from the user. Therefore, any missing data necessitates the removal of the entire data set [11]. The relative expression software tool (REST; [16]) is a stand-alone software tool to estimate up- and down-regulated gene expression. REST 2009 Software applies a mathematic model that takes into account the different PCR efficiencies of the gene of interest and reference genes. The software addresses issues surrounding the measurement of uncertainty in expression ratios by using randomization and bootstrapping techniques. Graphical output of the data via whisker-box plots provides a visual representation of variation for each gene that highlights potential issues such as a distribution skew [17].

The deliberate addition of ENMs to alter the specificity and efficiency of a PCR reaction has been reported [18, 19]. However, if the deliberate addition of an ENM may alter the mechanics of the PCR, then the unintentional intracellular (residual) amount of ENM will also alter the PCR assay. Thus, it is a form of assay interference that can generate false readings for gene expression based studies of toxic exposures. In fact, RT-qPCR has been used to assess cytotoxic responses to ENMs, but without any assay verification. This is surprising considering the recent review on the wide spread assay interference caused by NPs, which has a serious implication for nano-toxicity testing [20]. This lack of verification prompted our study presented herein.

In a previous study, altered gene expression in human lung epithelial cells (BEAS-2B) was observed after exposure to ZnO NPs of 20 nm [21]. It was found that sub-lethal concentrations of ZnO were able to increase the expression of apoptosis and oxidative stress responsive genes, i.e. *BNIP*, *PRDX3*, *PRNP* and *TXRND1*. They did use titanium dioxide as a positive control and also took into account interference caused by ZnO with the dye in the MTS assay. However, they did not mention testing for assay interference of fluorescence for ROS studies, or, absorbance for LDH testing. In addition, a pathway-specific qPCR-based method was used (SuperArray, Bioscience), by normalising to only *GAPDH* as the one reference gene. However, no prior testing was reported to verify if these nanoparticles (NPs) reacted with the assay itself. In another study, the effects of nano-copper oxide (CuO 0, 1, 10 and 50 mg/mL) were analysed via RT-qPCR [4]. A total of 13 reference gene candidates (*act*-*1*, *cdc*-*42*, *pmp*-*3*, *eif*-*3.C*, *actin*, *act*-*2*, *csq*-*1*, *Y45F10D.4*, *tba*-*1*, *mdh*-*1*, *ama*-*1*, *F35G12.2*, and *rbd*-*1*) were tested to determine their expression stability under the different doses. Four algorithms, geNorm, NormFinder, BestKeeper and the comparative ΔΔCt method, were employed to evaluate these 13 candidate expressions. As a result, only three genes were identified as the most reliable and, thus, were recommended to be used as reference genes in future studies of NP-induced genetic response using *C. elegans* (a nematode roundworm model system). Although they did screen for suitable reference genes based on stability, they still did not determine if any assay interference was caused by the presence of (residual) ENMs in the genetic material. A third study that assessed the genotoxic response in HeLa cells caused by a 48 h exposure to a silver NP-based hydrogel has also been reported [22]. RT-qPCR was used to verify the differentially expressed genes, where data was normalised to only one reference gene, actin, via the comparative ΔΔCt method. Again, no assay verification was reported in this study. In a last study, the cytotoxic, genotoxic and inflammatory responses to NPs from photocopiers was also assessed, where changes in gene expression data from THP-1 cells was determined using *18S* and *GAPDH* as internal controls via multiple internal control normalisation [23]. Yet again, no assay verification was reported in this study. Recently, a study that used carriers consisting of modified AuNPs in order to deliver siRNA, reported interference of the RT-qPCR assay [24]. These authors initially thought that it was due to an interaction between the AuNPs and the SYBR Green dye used in the PCR. However, when they repeated their experiment using a regular end-point RT-PCR reaction without any dye, they obtained the same results in the absence of SYBR Green. Hence, they referred to our own previous study where we have reported assay interference, i.e. that AuNPs could damage RNA during the isolation procedure [1].

**Fig. 1:**
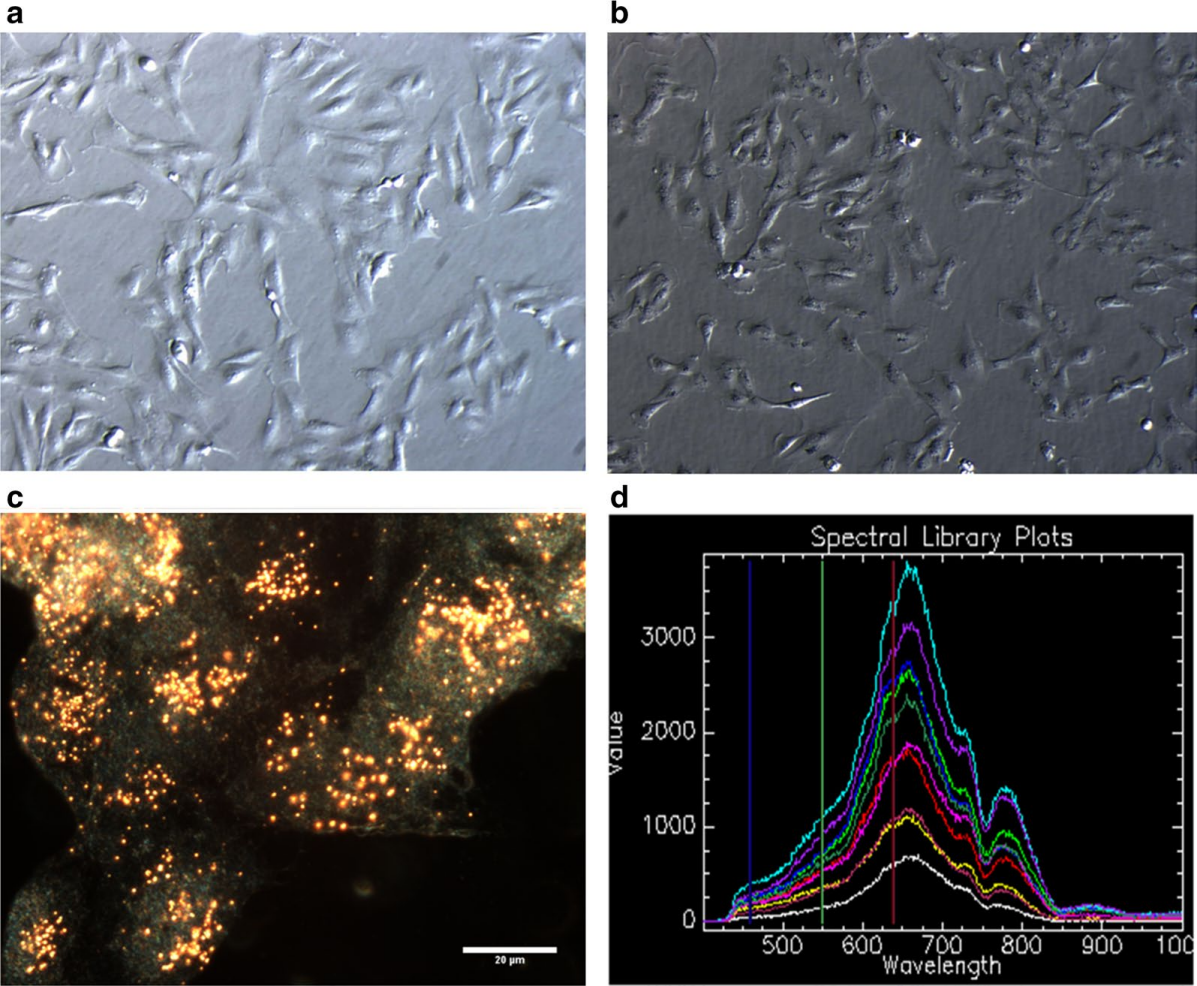
Representative microscopy of BEAS-2B cells treated with 1 nM of 14 nm citrate stabilised AuNPs (adapted from [1]). **a** Control untreated BEAS-2B cells, at × 10 magnification. **b** BEAS-2B cells treated with AuNPs for 24 h, at × 10 magnification. **c** Dark field image of BEAS-2B cells incubated with AuNPs for 24 h, at × 60 magnification (insert scale 20 µm). **d** The spectral profile of singularly dispersed and aggregated 14 nm citrate-stabilised AuNPs, collected from ten randomly selected particles

All the studies presented above raised concerns regarding the fact that no prior testing was reported to verify whether or not the NPs reacted with the assay itself. Hence, the results reported below are a continuation of our investigations on assay interference and verification. This is the first, step-for-step, detailed report of how the presence of AuNPs can alter the interpretation of the effects of AuNPs in a biological assay based on qPCR results. Our work was aimed at raising awareness regarding the interference caused by intracellular ENMs (see Fig. 1), which may remain in biological samples after isolation/purification procedures [1], so as to caution scientists who use RT-qPCR for expression profiling of toxicity pathway genes after exposure to any ENMs.

## Methods

The aim of this study was to verify the RT-qPCR procedure to test gene expression in human cell lines after treatment with AuNPs. A literature search narrowed the list of putative human reference genes down to ten candidates before initiating any wet-bench experiments. Thereafter, in silico analysis was performed in order to predict conformational changes under experimental conditions (see Additional file 1). A preliminary test was performed using BEAS-2B cells that had been treated for 24 h with a non-cytotoxic concentration of 1 nM AuNPs (see Additional file 2), since these are the conditions for all planned gene expression work yet to be performed. Thereafter, the RT-qPCR assay was verified by using RNA that had been spiked with known amounts of AuNPs. The universal RNA standard obtained from ten cell lines, which excluded BEAS-2B, was also used as the template in an RT-qPCR assay, using EVA green for HRM, in order to determine any assay interference that may have been caused by the AuNPs. The series of experiments performed during this verification step, were analysed in two different ways, i.e. based on either the point at which the AuNPs were added to the reaction, or, the software method of analysis used.

### Synthesis of AuNPs

The AuNPs were fully characterised as previously described [25]. These AuNPs were 14 nm in size and suspended in ultra-pure water, which is recognised as a reference sample (NM-330) by the OECD working party of the Manufactured Nanomaterials (WPMN) safety testing programme. Briefly, the AuNPs were prepared by Mintek (South Africa) with sodium citrate, where trisodium citrate aqueous solution (10 mL, 17 mM) was added to 180 mL (0.3 mM) of boiling HAuCl_4_·3H_2_O aqueous solution [26, 27]. The mixture was boiled under reflux for 15 min and allowed to cool to room temperature. The resultant citrate-capped AuNP suspension was stirred overnight at room temperature. The AuNP suspension was filtered using a 0.25 mm sterile syringe filter (Acrodisc 25 mm PF, 0.2 mm; nonpyrogenic) before use. The synthesis was performed under sterile conditions. Sterility of the AuNP samples were further confirmed by plating the samples on Tryptic soy agar (TSA) and incubating at 37 °C for 3 days in order to determine if any bacterial growth occurred. Tetrachloroaurate (HAuCl_4_·3H_2_O) and trisodium citrate (Na_3_C_6_H_5_O_7_·2H_2_O) were purchased from Sigma Aldrich (USA) and used without further purification.

### In silico analysis of primers

The in silico analysis was performed using the integrated DNA technologies (IDT) software. [28]. The target type was selected as “DNA” since the samples would be RNA that had been reverse transcribed into cDNA using the random hexamer and oligo-dT primers. The determining factor for the selection was based on the structure with a Gibbs free energy within acceptable limits (see Additional file 1).

### Overview of the preliminary exposure study

For the preliminary study, total RNA was isolated from a treated bronchial epithelial human cell line, BEAS-2B, as previously reported [1]. Briefly, BEAS-2B cells were seeded at 3 × 10^4^ cells/cm^2^ in a 75 cm^2^ flask and allowed to proliferate for 24 h before treatment. The samples consisted of three biological repeats, where BEAS-2B cells were treated with AuNPs for 24 h (see Additional file 2). This RNA was reverse transcribed to generate cDNA and then amplified using ten reference gene primer pairs. The BestKeeper analysis is shown in Additional file 2: Table S1. The NormFinder analysis is indicated in Additional file 2: Table S2. The REST analysis is shown in Additional file 2: Figure S1. The CFX Manager software was used to obtain the PCR efficiency (E), the linearity of the PCR assay (R^2^) as well as the slope obtained for the standard curve, which is summarised in Additional file 2: Tables S3, S4. The melt peak for each primer pair is shown in Additional file 2: Figures S2–S6.

In order to visualise the internalisation of the AuNPs, CytoViva dark field microscopy and HIS was used, as previously reported [1]. For these uptake studies, cells were seeded in 8-well Millicell EZ slides (Millipore, Germany) prior to treatment. Following incubation, cells were immobilized onto the slides. Dark field images were captured at 60 × magnification using the CytoViva 150 Unit integrated onto the Olympus BX43 microscope. Images were acquired using a Dagexcel 616 camera and the associated software (see Fig. 1).

To investigate the AuNPs that remain after purification of RNA, a drop of either AuNP in Milli-Q water or the isolated RNA from AuNP-treated cells was placed onto a Millicell EZ slide and allowed to dry. Both samples were visualized using dark-field microscopy as described above. A spectral library of the 14 nm AuNPs was created by randomly selecting spectra of AuNPs, where each spectrum of the library represents a single pixel obtained from the HSI scan, as previously reported [1]. In order to investigate the presence of the AuNPs in the RNA sample, the image classification algorithm, spectral angle mapper (SAM), was performed using ENVI software to map the spectral libraries onto the scans. Therefore, the spectral profile collected was representative of a spectral library of the AuNP onto the HSI scan of the RNA (see Fig. 1).

### RNA isolation, quantification and integrity analyses

For the preliminary study, total RNA was isolated using the RNeasy plus mini kit (Qiagen, GmbH), according to the manufacturer’s instructions. In addition, the QIAshredder spin columns (Qiagen, GmbH) were used to homogenize the samples. Since it takes time to both treat and process samples over the course of a time study, the RNAprotect stabilizing solution was used for all samples in order to minimise variations during the incubation and storage time periods. Following trypsinization and harvesting of the cells, RNAprotect solution was added to intact cells to stabilize the RNA. The RNA lysis buffer with guanidine thiocyanate was added and vortexed to lyse the cells. The cell lysate was passed through a QIAshredder column to aid homogenization. Thereafter, this eluent was passed through a gDNA Eliminator column to remove genomic DNA. Ethanol was added and the sample loaded onto an RNeasy MinElute column, where RNA binds to the column and contaminants were washed away during subsequent wash steps with the RNA wash buffer and the RNA ethanol-based buffer. Finally, RNA was eluted with RNase-free water. Each experiment was performed on a fresh isolation of RNA from BEAS-2B cells from a different passage number, i.e. completely separate experiments, where each time a new cDNA pool was reverse transcribed and amplified.

For the assay verification of the study conditions and parameters, a universal human reference total RNA standard was purchased for qPCR (Agilent Technologies, USA). This qPCR human reference total RNA was composed of total RNA from ten human cell lines, with quantities of RNA from the individual cell lines optimized to maximize representation of gene transcripts present in low, medium, and high abundance. The cell line derivations included an adenocarcinoma (mammary gland), hepatoblastoma (liver), adenocarcinoma (cervix), embryonal carcinoma (testis), glioblastoma (brain), melanoma (skin), liposarcoma, histiocytic lymphoma (macrophage, histocyte), lymphoblastic leukaemia (T lymphoblast) and plasmacytoma (myeloma, B lymphocyte). According to the manufacturer, this reference RNA was carefully screened by spectrophotometry, MOPS agarose gel electrophoresis and analysis using the Agilent 2100 Bioanalyzer. In addition, the RNA was manufactured in large batch-lots in order to eliminate inconsistencies over long-term experiments, and, was treated with DNAse. Each experiment was performed on a fresh aliquot of the same universal RNA standard, where each time a new cDNA pool was reverse transcribed and amplified. It should be noted that the universal RNA standard does not include BEAS-2B, which was the template used for the preliminary study (see Additional file 2).

### AuNP treatments

As explained directly above, AuNP treatments consisted of either exposure of BEAS-2B cells, or, as deliberate applications to the RNA standard. For the preliminary study, BEAS-2B cells were treated with a non-cytotoxic concentration of 1 nM AuNPs for 24 h (see Additional file 2), as determined by cell impedance analyses [25]. For the assay verification, the AuNPs were added to the RT-qPCR reaction, i.e. spiked at 25, 50 and 75% vol/vol, where the final concentration (FC) in a final PCR volume of 40 µL was 0.72 nM, 1.44 nM and 2.2 nM, respectively. These various amounts of AuNPs were either added to the universal RNA standard at the reverse transcription step (part 1 of assessment), or, spiked at the PCR amplification step (part 2 of assessment), respectively.

### cDNA synthesis

For the preliminary study, 1 µg of total RNA was extracted as previously reported [1]. The first strand cDNA was transcribed using an oligo-dT primer and random hexamers synthesised by IDT (USA; see Table 1), with SuperScript III Reverse transcriptase (Invitrogen, USA), according to the manufacturer’s instructions.

**Table 1:** List of primers and sequences (according to HUGO gene nomenclature; http://www.genenames.org)

Primer name (abbreviated) and NCBI RefSeq	Forward primer sequence and Tm	Reverse primer sequence and Tm	Amplicon size (bp)	References
18S (NR_003286.2; NT_167214.1)	5′-AGAAACGGCTACCACATCCA-3′ 56.3 °C	5′-CACCAGACTTGCCCTCCA-3′ 57.3 °C	169	[8]
ACTB (NM_001101)	5′-AGAAAATCTGGCACCACACC-3′ 55.6 °C	5′-TAGCACAGCCTGGATAGCAA-3′ 56.1 °C	173	[9]
GAPDH (NM_002046)	5′-CGACAGTCAGCCGCATCTT-3′ 57.8 °C	5′-CCCCATGGTGTCTGAGCG-3′ 58.5 °C	63	[8]
GUSB (NM_000181)	5′-AGCCAGTTCCTCATCAATGG-3′ 54.9 °C	5′-GGTAGTGGCTGGTACGGAAA-3′ 56.8 °C	160	[8]
HPRT1 (NM_000194)	5′-TGACACTGGCAAAACAATGCA-3′ 56.0 °C	5′-GGTCCTTTTCACCAGCAAGCT-3′ 58.0 °C	94	[8]
HSP90 (NM-007355)	5′-TCTGGGTATCGGAAAGCAAGCC-3′ 59.4 °C	5′-GTGCACTTCCTCAGGCATCTTG-3′ 58.4 °C	80	[8]
PPI (NM_021130)	5′-AGACAAGGTCCCAAAGAC-3′ 51.6 °C	5′-ACCACCCTGACACATAAA-3′ 50.7 °C	118	[8]
SDH (NM004168)	5′-TGGGAACAAGAGGGCATCTG-3′ 57.2 °C	5′-CCACCACTGCATCAAATTCATG-3′ 54.8 °C	86	[8]
TBP (NM_003194)	5′-TGCACAGGAGCCAAGAGTGAA-3′ 58.9 °C	5′-CACATCACAGCTCCCCACCA-3′ 59.8 °C	132	[8]
YWHAZ (NM_003406)	5′-ACTTTTGGTACATTGTGGCTTCAA-3′ 55.5 °C	5′-CCGCCAGGACAAACCAGTAT-3′ 57.4 °C	94	[8]

A universal RNA standard from Stratagene (Agilent, USA) was used to verify the qPCR assay. The RNA was spiked at two different points, either the reverse transcription step (part 1), or, at the DNA amplification step (part 2). In this manner, the transcription and amplification efficiency was assessed in response to the addition of AuNPs, at various concentrations. Specifically, a 1:1 ratio of an oligo-dT primer and random hexamer (IDT, USA), was used to reverse transcribe 1 µg of the RNA Standard, using SuperScript III Reverse transcriptase (Invitrogen, USA), according to the manufacturer’s instructions. Assay interference of the reverse transcription (caused by the AuNPs), was assessed by analysing the resulting PCR efficiency percentage, the linearity of the PCR assay as well as the gradient or slope obtained for the standard curve.

### RT-qPCR

For the preliminary study, cDNA was generated from the RNA that was isolated after BEAS-2B cells were exposed to 1 nM AuNPs for 24 h. However, in order to verify the assay, the resulting cDNA from the RNA standard was also amplified using specific primers (IDT, USA), as indicated below in Table 1 with the associated NCBI GenBank accession reference sequence.

The SsoFast EvaGreen qPCR super-mix was used in a CFX96 thermocycler with high resolution melt (HRM) capabilities (Biorad, USA). The cycling conditions include: enzyme activation at 95 °C for 30 s, followed by 35 cycles of denaturation at 95 °C for 5 s, primer annealing at 60 °C for 5 s and primer extension at 72 °C for 5 s, with a final melt from 50 to 95 °C (in 0.2 °C increments). The reference genes were selected based on a literature review specific to examples where RT-qPCR genetic studies were used in human cell lines, in addition to those genetic studies performed to assess the effects of ENMs [4, 8, 9, 22, 23, 29]. All primers had an annealing temperature (T_a_) of 60 °C, in order to analyse all variables in one experimental run and to create a uniform experimental condition for future diagnostic applications. Assay interference caused by the AuNPs was assessed by analysing the PCR products via different algorithms and qPCR statistical software.

### Agarose gel electrophoresis

The resulting amplicons obtained from the spiked RNA (part 1 of the assessment) and spiked cDNA (part 2) were separated on a 1% agarose gel (see Additional file 3). The gel was subjected to electrophoresis at 100 V, whilst being submerged in 89 mM Tris-borate and 2 mM EDTA at pH 8.3 (TBE) buffer (Sigma Aldrich, USA) and was stained with 10 µg/mL ethidium bromide. Images were obtained using GeneSys software version1.3.3.0 on a Syngene G:Box instrument (grey-scale).

### Gene expression and statistical analyses

The expression data generated was analysed using traditional algorithms and qPCR statistical software, i.e. BestKeeper, NormFinder, REST and qBase+. The first software processed data based on the crossing points or C_q_ [30]. Therefore, the expression of the reference genes was quantified separately. The PCR efficiency may be calculated using the qPCR Standard Curve Slope to Efficiency Calculator [31]. The standard deviation and coefficient of variance were calculated and used to rank the candidate reference genes. All those genes that were considered to be stably expressed were combined and the geometric mean used to create the BestKeeper Index. The geometric mean refers to the central number in a geometric progression, calculated as the nth root of a product of “n” numbers. Correlation between each candidate reference gene and this index was calculated and can be described by the Pearson correlation coefficient. The NormFinder software ranked candidate reference genes according to their respective expression stability within the given sample group and experimental design [32]. Firstly, a standard curve was generated. Thereafter, the C_q_ values of the possible reference genes were plotted on the standard curve in order to obtain linear scale expression quantities. This linear data was used as the input for the program, which then estimates the overall expression variation, as well as, variation between sample subgroups (e.g. untreated and treated samples, or, normal and cancer samples). A stability value was generated for each gene, within the given sample group and experimental design. The REST software program processed the Cq input data. This software applied integrated randomization and bootstrapping methods to test the statistical significance of calculated expression ratios, even when the data included outliers. Therefore, it takes into account the different PCR efficiencies of the gene of interest and reference genes. Expression variation for each gene was visualized in a whisker-box plot to highlight potential issues, such as a distribution skew. The manual assessment of the range of the C_q_ results was based on data generated using the CFX Manager™ software (Biorad, version 3.0) [33]. In the qBase+ software program, a geNorm pilot study can be used to determine similar expression profiles within a group, between treated and untreated samples. The qBase+ software determines the average expression stability of the reference genes, which was visualised as a geNorm M plot, where very high reference target stability has an average geNorm ≤ 0.2. This indicates the average expression stability value “M” for the reference genes at each step during a stepwise exclusion of the least stably expressed gene. Therefore, the genes are ranked according to increasing expression stability, i.e. the least stable gene starts on the left side and the plot ends with the most stable genes on the right. The internal quality control (QC) check classifies reference gene stability as “good to normalize” when the geNorm M value is ≤ 0.2. This QC check will indicate when the results do not meet the threshold cut-off, i.e. reference genes are not stable due to high values when the geNorm M value is 0.5. In addition, the geNorm V chart illustrates the average levels of variation for reference gene stability, i.e. the normalisation factor is calculated with a sequential addition of each gene to the equation. This output is an indication of the minimum number of reference genes required for normalisation. The measure is a pair wise variation (V), starting with the most stably expressed genes on the left, then moving to the right with the inclusion of a 3rd, 4th, 5th gene etc. The optimal reference target selection in an experiment is selected based on geNorm V < 0.15, when comparing the normalisation factor.

## Results

This is the first detailed report indicating, in a step-for-step manner, how the presence of intracellular residual AuNPs can alter the interpretation of the biological effect of AuNPs in an assay based on qPCR results. The in silico analysis was performed in order to predict conformational changes under experimental conditions. This analysis predicted that the primers for *18S*, *GUSB*, *HSP90*, *SDH a*nd *YWHAZ* should produce acceptable PCR products (see Additional file 1).

A preliminary test was performed using BEAS-2B cells that had been treated with a non-cytotoxic concentration of 1 nM AuNPs. It was observed that the AuNPs localised within these cells (see Fig. 1). The RNA obtained from these cells was used to assess the amplification of reference genes with SYBR Green in order to screen the primers (see Additional file 2). Assessment of the amplification of the reference genes, as influenced by AuNPs, included use of the CFX Manager software to obtain the PCR efficiency, the linearity of the assay as well as the slope obtained from the standard curve. The melt curves were also analysed. The preliminary study confirmed that the primers could amplify the required amplicons in accordance with guidelines for minimum information for publication of quantitative real-time PCR experiments (MIQE) [6]. However, it was suspected that the AuNPs caused interference. This preliminary study has, therefore, lead to the requirement for RT-qPCR method validation. As a result, it was decided to test the primers again, but instead using an RNA standard that had been deliberately spiked with AuNPs.

The RT-qPCR assay was verified using RNA that had been spiked with known amounts of AuNPs, in order to determine any degree of error for the measurements. This treatment mimics exposure assessment studies, where assay-interference may be caused by intracellular residual ENMs still being present in the biological samples (during and after isolation/purification procedures). Specifically, the universal RNA standard obtained from ten cell lines was used as the template in an RT-qPCR assay (using EVA green for HRM), in order to determine any assay interference that may have been caused by the AuNPs. The series of experiments performed were analysed based on:

A. The point at which the AuNPs were added to the reaction, i.e. either spiked at the reverse transcription step (part 1), or, spiked at the PCR amplification step only (part 2). This method assesses the effect that AuNPs could have on RT-qPCR if AuNPs were internalised in cell lines, i.e. it mimics the situation where AuNPs are internalised and may interact with cytosolic cellular content of mRNA (exposed during translation), or, single-strand DNA (exposed during cell replication).
B. The method of analysis used, i.e. BestKeeper, NormFinder, REST, or the CFX Manager™ and qBase+ software. All were used for both part 1 and part 2 mentioned above.

It should be noted that most reports on the possible effect on gene induction, as caused by AuNPs, are based on the assumption that the cells are still intact. However, internalised AuNPs (see Fig. 1), may interact and have an effect on the exposed cellular contents after cell lysis and during the assay procedure. This would cause an unintentional effect on the assay [1], which produces false interpretations of toxicity. Hence, the controls in this study included “non-functionalised ENMs”, represented by citrate stabilised AuNPs, as well as, samples with “no ENMs”, represented by the 0% AuNP samples.

### AuNP-spiking of the reverse transcription step

In the first series of experiments (part 1), the universal RNA standard was spiked with AuNPs, before reverse transcription, which generates cDNA. This treatment has biological significance because it mimics any possible interference of intracellular residual ENMs present in biological samples, which would co-precipitate with isolated RNA intended for analyses during exposure assessment studies. Hence, this design should determine if any assay-interference is caused by the AuNPs. A mix of an oligo-dT primer and a random hexamer was used so that the conditions could be as generic as possible, in order to design an “open”-assay procedure for multiple future uses. Amplification was performed with EVA Green in order to perform HRM analyses.

**Fig. 2:**
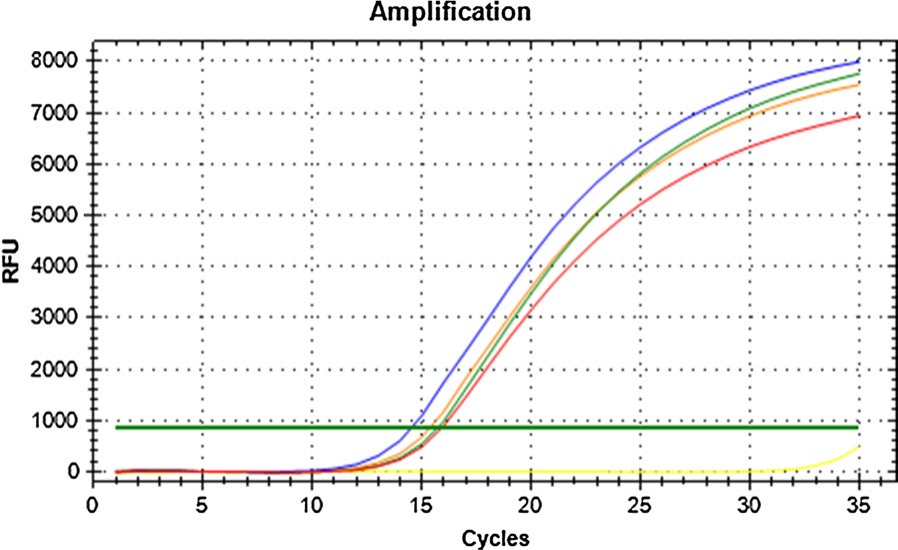
Representative amplification plot using the CFX Manager™ software (Biorad, version 3.0) for one gene (*18S*) in the absence and presence of various amounts of AuNPs. Before the addition of AuNPs, blue represents 0%; After the addition of AuNPs, green represents 25%; orange represents 50%; red represents 75% AuNPs; Without DNA, yellow represents the non-template control (NTC)

**Fig. 3:**
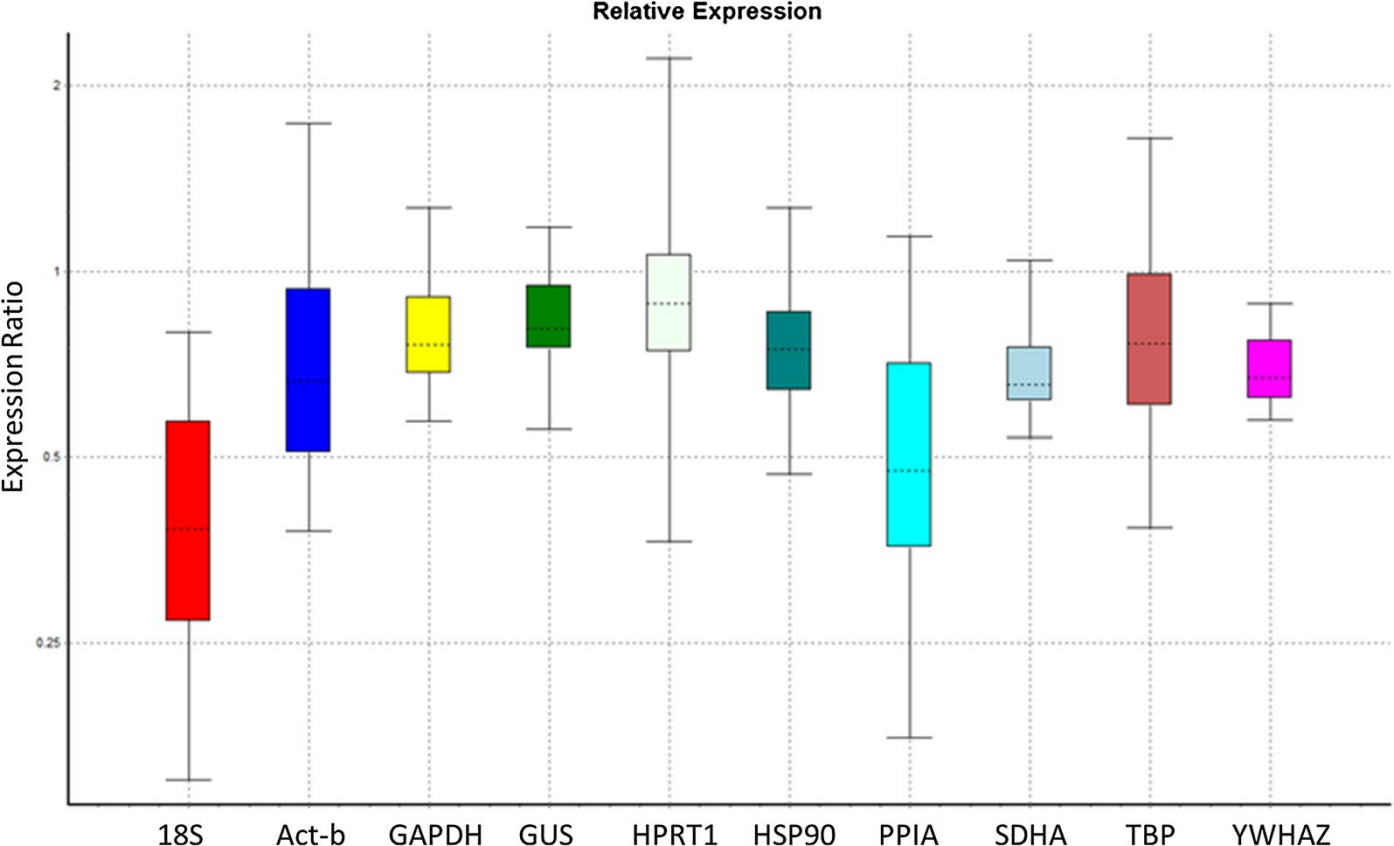
Representative results for REST analysis showing the expression ratio vs. the gene, where the RNA was spiked before transcription (part 1), based on the best efficiency for that reference gene. Multiple standard curves, from multiple experiments, create multiple efficiency values that are difficult for the software to process

Initial observations of only the amplification plots indicated a change in the profiles for some of the reference genes, i.e. before and after deliberate addition of AuNPs (see Fig. 2; Additional file 3). Thereafter, the stability of the reference genes was assessed by qPCR specific software programs. The BestKeeper software program ranked the reference genes from a pool of ten candidates (see Table 2). All C_q_ were compared over the entire study, including the control and all the treatment groups. Therefore, a biological triplicate, with 4 different treatments, generated 12 data points (*n* = 12). The NormFinder software program processed an average in order to rank the genes based on three separate analyses performed (see Table 3). However, the global summary refers to the average of the triplicates, where all data was analysed together as one data set, which then differs from separate or individual assessments. The REST software program generated a graphical output of the data, see Fig. 3.

**Table 2:** Summarised results of the universal RNA standard spiked before transcription (part 1), which shows initial descriptive statistics and secondary correlations, as generated by BestKeeper analyses

	Least stable									Most stable
SD	*P* *P* *I *(0.61)	*18S* (0.59)	*HPRT1* (0.53)	*ACTB* (0.42)	*TBP* (0.36)	*HSP90* (0.27)	*GAPDH* (0.26)	*YWHAZ* (0.26)	*SDH* (0.26)	*GUSB* ^a^ (0.23)
CV	*1* *8* *S* (3.80)	*PPI* (3.15)	*ACTB* (2.36)	*HPRT1* (2.33)	*GAPDH* (1.46)	*TBP* (1.44)	*HSP90* (1.32)	*YWHAZ* (1.22)	*SDH* (1.09)	*GUSB* ^b^ (0.96)
*r*	*HPRT1* (0.396)	*TBP* (0.499)	*GAPDH* (0.529)	*ACTB* (0.546)	*PPI* (0.630)	*GUSB* (0.681)	*HSP90* (0.797)	*YWHAZ* (0.875)	*SDH* (0.903)	*18S* ^c^ (0.957)

SD: standard deviation (^a^ stability = ranked low to high)

CV: coefficient of variance (^b^ stability = ranked low to high)

*r*: Pearson correlation coefficient (^c^ stability = ranked high to low)

**Table 3:** Summarised results of the universal RNA standard spiked before transcription (part 1), as generated by the NormFinder analyses

Reference gene	NormFinder technical REPEAT 1	NormFinder technical REPEAT 2	NormFinder technical REPEAT 3	NormFinder average	Manual ranking using the NormFinder average of the reference gene	NormFinder global summary of the best gene^a^
*18S*	(0.679)	(0.314)	(0.131)	0.374	1 *SDH* (most stable) 2 *YWHAZ* 3 *GUSB* 4 *TBP* 5 *HSP90* 6 *GAPDH* 7 *HPRT1* 8 *ACTB* 9 *18S* 10 *PPI* (least stable)	*GUSB* and *HSP90* (0.121) *GUSB* (0.126) *HSP90* (0.136)
*ACTB*	(0.368)	(0.212)	(0.118)	0.232		
*GAPDH*	(0.180)	(0.175)	(0.099)	0.151		
*GUSB*	(0.198)	(0.088)	(0.037)	0.107		
*HPRT* *1*	(0.202)	(0.149)	(0.169)	0.172		
*HSP90*	(0.094)	(0.118)	(0.155)	0.122		
*PPI*	(0.246)	(0.668)	(0.260)	0.391		
*SDH*	(0.063)	(0.078)	(0.070)	0.070		
*TBP*	(0.095)	(0.145)	(0.106)	0.115		
*YWHAZ*	(0.197)	(0.038)	(0.029)	0.088		

^a^The best gene has the lowest stability value

In addition to traditional qPCR analysis software and statistical programs, a manual assessment was also performed. The CFX Manager software was used to obtain the PCR efficiency, the linearity of the PCR assay as well as, the gradient or slope obtained for the standard curve, which is summarised below in Table 4. The reference gene expression was screened to determine changes in the dissociation assay melt peaks of the different products formed (see Additional file 3). The manual assessment yielded results that fell within acceptable parameters. However, this assessment was extended further to determine whether or not AuNPs could interfere with the assay. The increasing amounts of AuNPs lead to changes in how quickly the genes were amplified, indicated by changes in the C_q_ (see Table 5).

**Table 4:** Summarised results of the universal RNA standard spiked before transcription (part 1), obtained using CFX Manager software

	18S	ACTB	GAPDH	GUSB	HPRT1	HSP90	PPI	SDH	TBP	YWHAZ
E (90 to 110%)^a^	***98.9*** to *126.3%*	***99.7*** to *158.7%*	*82.2* to *89.1*%	***102.7*** to ***105.7***%	***91.3*** to ***94.7***%	***100*** to ***101.5***%	***104*** to *127.7*%	*125.2* to *146.6*%	***99.7*** to ***101.2***%	*81.7* to *88.2*%
R^2^ (> 0.980)^b^	*0.973* to ***0.996***	*0.834* to *0.996*	*0.955* to ***0.997***	*0.960* to ***0.993***	***0.981*** to ***0.999***	***0.987*** to ***0.997***	0.969 to ***0.992***	*0.910* to ***0.996***	***0.992*** to ***0.997***	***0.984*** to ***0.995***
Slope (− 3.1 to − 3.6)^a^	− *2.819* to − ***3.348***	− *2.422* to − ***3.330***	***−*** ***3.614*** to − *3.842*	***−*** ***3.193*** to ***−*** ***3.258***	***−*** ***3.457*** to ***−*** ***3.507***	***−*** ***3.286*** to ***−*** ***3.321***	− *2.798* to −***3.226***	− *2.551* to − *2.837*	***−*** ***3.236*** to ***−*** ***3.329***	− 3.642 to − 3.855
NTC C_q_	*35.85 to 38*	***N/A***	***N/A***	***N/A***	***N/A***	***N/A***	***N/A***	***N/A***	***N/A***	***N/A***

*Bold italics* acceptable result;* italics* unacceptable result

^a^For a PCR efficiency (E) of 100%, the slope is − 3.32. A good reaction should have an efficiency between 90 and 110%, which corresponds to a slope between − 3.58 and − 3.10

^b^The linearity of the assay (R^2^), where R^2^ < 0.980 unacceptable; R^2^ ≥ 0.980 acceptable; R^2^ > 0.990 expected; R^2^ > 0.995 exceptional

**Table 5:** Summarised C_q_ results for AuNP-interference assessment of the universal RNA standard spiked before transcription (part 1), by using CFX Manager™ software

Reference gene C_q_^a^	18S	ACTB	GAPDH	GUSB	HPRT1	HSP90	PPI	SDH	TBP	YWHAZ
0%AuNP	13.81–14.90	17.24–18.02	17.36–17.71	23.40–24.02	21.98–23.07	19.53–19.90	18.15–18.73	23.39–23.65	24.48–25.22	21.06–21.24
25%AuNP	***15.23***–*15.71*	***17.55–18.11***	***17.82–18.14***	***23.64–24.18***	***22.06–23.32***	***19.85–*** *20.43*	***18.55–*** *19.48*	***23.79–24.11***	***25.06–25.52***	***21.45–*** *21.94*
50%AuNP	15.36–*16.57*	***17.22–*** *18.53*	***17.34–*** *18.24*	***23.82–24.31***	*22.21*–***23.49***	***20.02–*** *20.62*	*19.24*–*20.66*	***23.59–24.11***	***24.50–*** *25.86*	***21.43–21.73***
75%uNP	*15.65*–*16.44*	***18.22–*** *18.64*	***17.50–17.96***	***23.74–24.36***	***21.88–23.38***	***19.56–20.32***	***19.22–*** *20.22*	***24.09–*** *24.28*	***24.69–*** *25.78*	***21.64–*** *21.87*

*Bold italic* acceptable result;* italics* unacceptable result;* plain text* base-line result, e.g. for untreated/0%AuNP samples

^a^A C_q_ change of 0.2 is acceptable, but > 0.5 is unacceptable

**Fig. 4:**
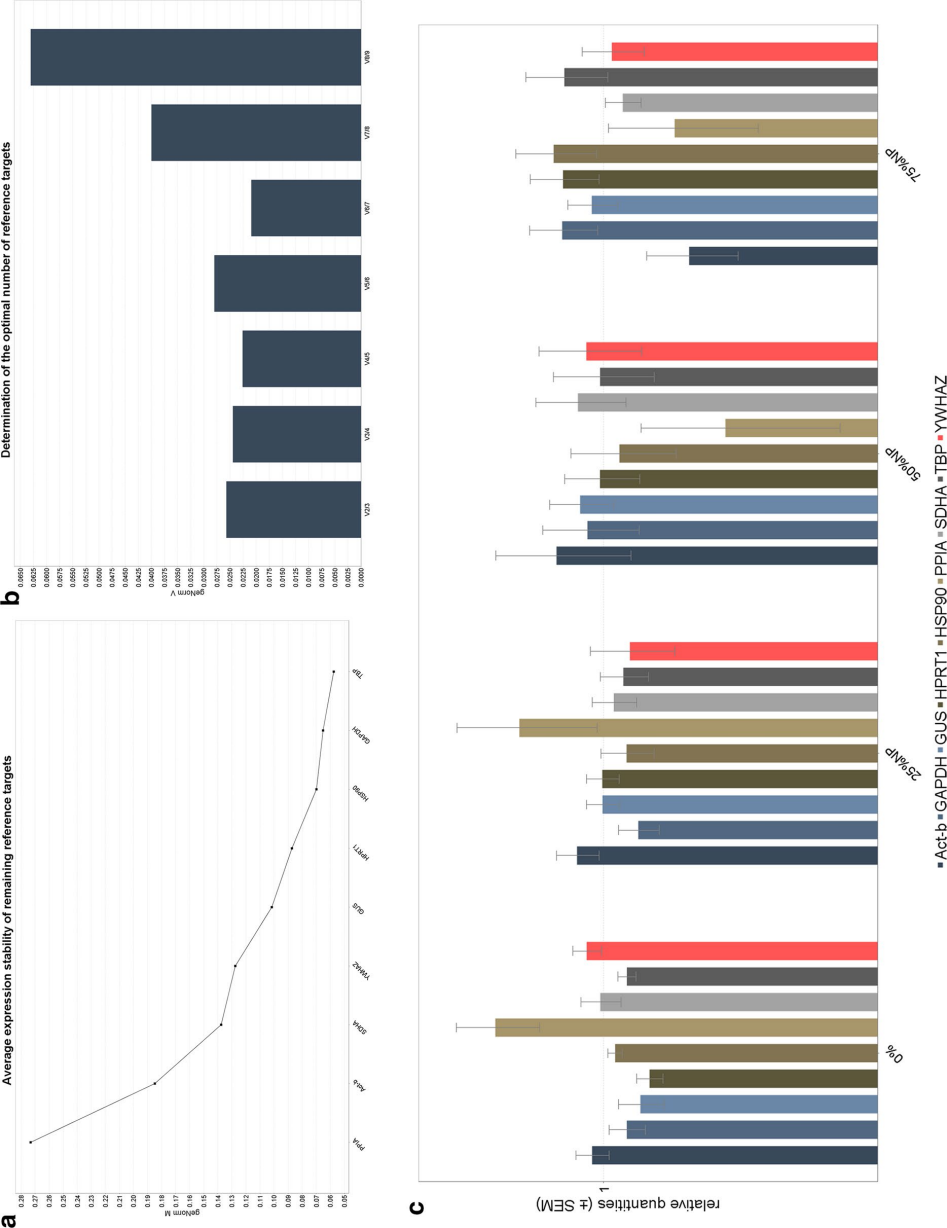
Representative results for qBase+ results obtained for samples that were spiked before transcription (part 1). **a** The results obtained for the geNorm M analyses. **b** The results obtained for the geNorm V analyses. **c** The geNorm multi-target bar chart with error bars

The qBase+ software program processed the data (see Fig. 4), where the average expression stability or geNorm M results are displayed as a chart (see Fig. 4a). The geNorm V chart illustrates the average levels of variation for reference gene stability (see Fig. 3b). The last output generated by the software was a multi-target bar chart for each reference gene, grouped according to the AuNP treatment (see Fig. 4c).

### AuNP-spiking of the DNA amplification step only

In the second series of experiments (part 2), the universal RNA standard was reverse transcribed to generate cDNA. Again, a mix of an oligo-dT primer and a random hexamer was used. However, the amplification step was spiked with AuNPs and the DNA was amplified using an enzyme cocktail including EVA Green in order to perform HRM analyses. This represented a technical control sample within the experiment. This method also assesses the effect that AuNPs could have on RT-qPCR in the event that AuNPs were internalised, i.e. it mimics the situation where AuNPs may interact with single-strand DNA, which is usually exposed during cell replication. Please note, any residual ENMs would usually be present after the isolation procedure and before reverse transcription. It should also be noted that only a 25% AuNP spike was added to the amplification step due to restrictions on the final volume of the reaction.

**Fig. 5:**
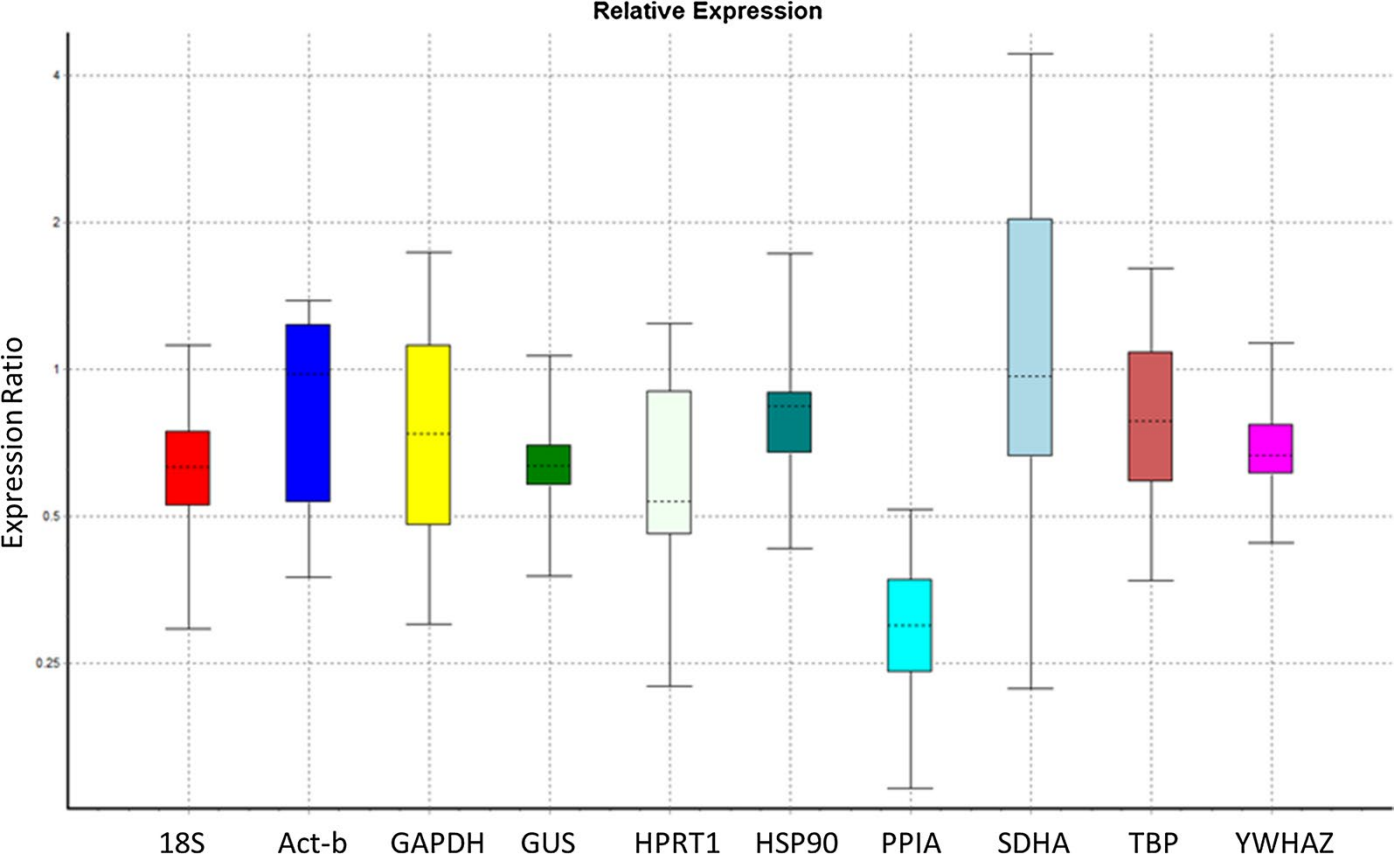
Representative results for REST analysis showing the expression ratio vs. the gene, where the universal RNA standard was spiked at the amplification step (part 2). Multiple standard curves, from multiple experiments, create multiple efficiency values that are difficult for the software to process

**Fig. 6:**
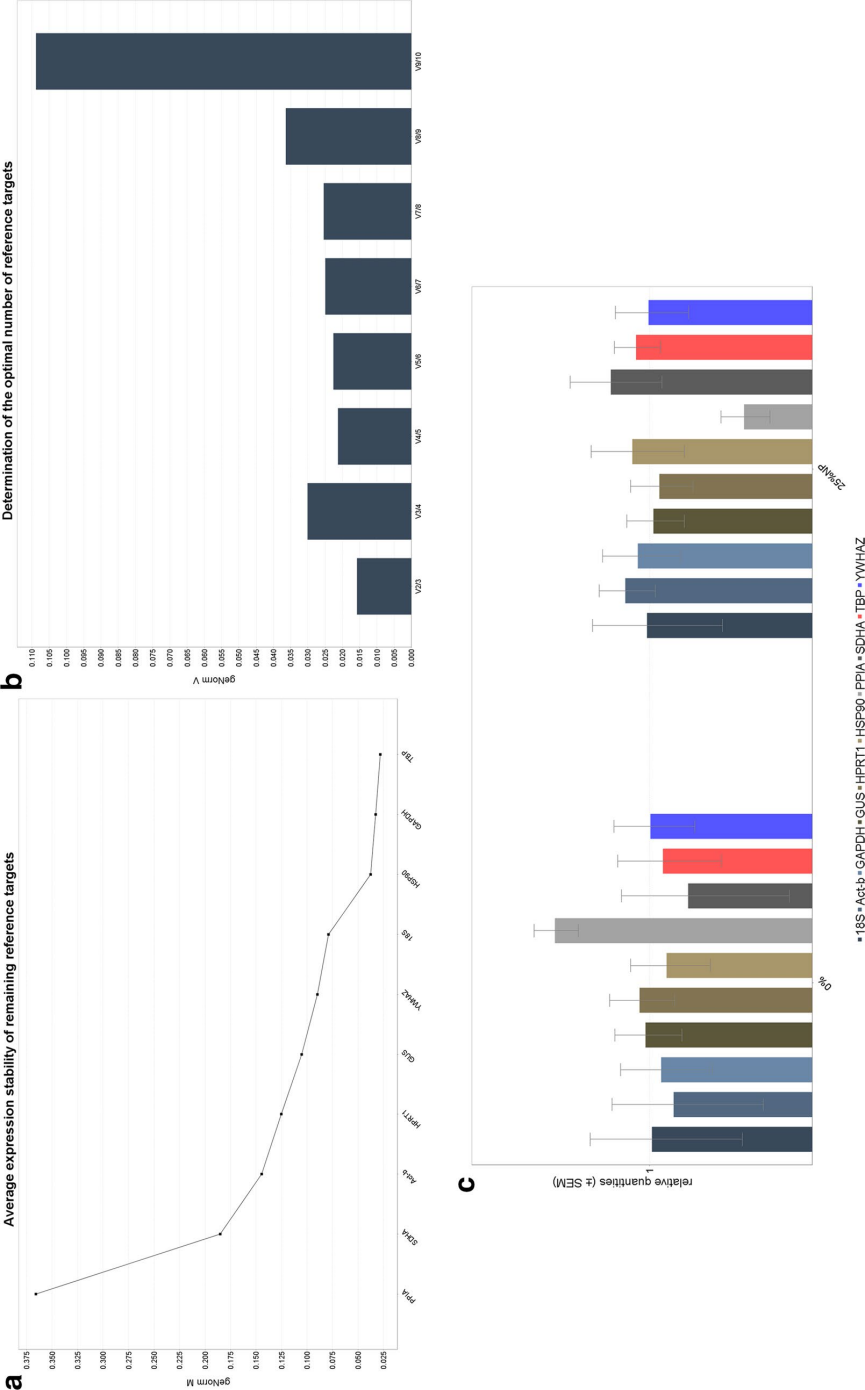
Representative results for qBase+ results obtained for samples that were spiked at the amplification step (part 2). **a** The results obtained for the geNorm M analyses. **b** The results obtained for the geNorm V analyses. **c** The geNorm multi-target bar chart with error bars

As mentioned in the previous section, the amplification plots identified changes in the PCR profiles for the reference genes, i.e. before and after deliberate addition of 25% AuNPs (see Additional file 3). Thereafter, the same software programs were used again. In addition, results indicating changes caused by 25% AuNPs at the amplification step only (part 2), were summarised (see Figs. 5, 6; Tables 6, 7, 8, 9).

**Table 6:** Summarised results of the universal RNA standard spiked at the amplification step (part 2), showing initial descriptive statistics and secondary correlations, as generated by BestKeeper analyses

	Least stable									Most stable
SD	*PPI* (0.89)	*SDH* (0.63)	*GAPDH* (0.50)	*HPRT1* (0.46)	*HSP90* (0.42)	*TBP* (0.40)	*YWAHZ* (0.37)	*ACTB* (0.36)	*18S* (0.36)	*GUSB* ^a^ (0.35)
CV	*PPI* (4.56)	*GAPDH* (2.78)	*SDH* (2.67)	*18S* (2.54)	*HSP90* (2.10)	*ACTB* (2.02)	*HPRT1* (1.99)	*YWHAZ* (1.74)	*TBP* (1.58)	*GUSB* ^b^ (1.46)
*r*	*YWHAZ* (0.634)	*PPI* (0.694)	*SDH* (0.729)	*ACTB* (0.821)	*GAPDH* (0.845)	*HPRT1* (0.882)	*HSP90* (0.906)	*TBP *(0.913)	*GUSB* (0.980)	*18S* ^c^ (0.994)

SD: standard deviation (^a^ stability = ranked low to high)

CV: coefficient of variance (^b^ stability = ranked low to high)

*r*: Pearson correlation coefficient (^c^ stability = ranked high to low)

**Table 7:** Summarised results of the universal RNA standard spiked during amplification (part 2), as generated by the NormFinder analyses

Reference gene	NormFinder technical repeat 1	NormFinder technical repeat 2	NormFinder technical repeat 3	NormFinder average	Manual ranking using the NormFinder average of the reference gene	NormFinder global summary of the best gene^a^
*18S*	(0.040)	(0.007)	(0.011)	0.019	1 *18S* (most stable) 2 *HPRT1* 3 *GUSB* 4 *YWHAZ* 5 *GAPDH* 6 *HSP90* 7 *ACTB* 8 *TBP* 9 *SDH* 10 *PPI* (least stable)	*GUSB* (0.093) *HPRT1* and *YWHAZ* (0.069)
*ACTB*	(0.467)	(0.151)	(0)	0.206		
*GAPDH*	(0.074)	(0.007)	(0.185)	0.088		
*GUSB*	(0.014)	(0.021)	(0.070)	0.035		
*HPRT1*	(0.078)	(0.017)	(0)	0.031		
*HSP90*	(0.035)	(0.017)	(0.368)	0.14		
*PPI*	(0.999)	(0.893)	(0.519)	0.803		
*SDH*	(0.286)	(0.502)	(0.158)	0.315		
*TBP*	(0.069)	(0.214)	(0.415)	0.232		
*YWHAZ*	(0.074)	(0.024)	(0.114)	0.070		

^a^The best gene has the lowest stability value

**Table 8:** Summarised results for the universal RNA standard spiked at the amplification step (part 2) during AuNP-interference assessment, using CFX Manager™ software

	18S	ACTB	GAPDH	GUSB	HPRT1	HSP90	PPI	SDH	TBP	YWHAZ
E (90 to 110%)^a^	*116* to *124.7*	*84.7* to ***106.8***	*84.2* to ***98.2***	***108.8*** to *117.5*	***96.2*** to ***100.9***	***99.1*** to ***106.4***	***105.3*** to *154.1*	*135.0* to *145.9*	***105.6*** to *364.8*	***90.0*** to ***98.5***
R^2^ (> 0.980)^b^	***0.994*** to ***1.000***	*0.961* to ***0.998***	***0.995*** to ***1.000***	***0.986*** to ***1.000***	***0.990*** to ***1.000***	***0.995*** to ***1.000***	*0.970* to ***1.000***	*0.956* to ***0.994***	*0.945* to ***0.997***	***0.989*** to ***1.000***
Slope (− 3.1 to − 3.6)^a^	− *2.845* to − *2.986*	***−*** ***3.169*** to − *3.751*	***−*** ***3.365*** to − *3.770*	− *2.964* to ***−*** ***3.129***	***−*** ***3.300*** to ***−*** ***3.416***	***−*** ***3.177*** to ***−*** ***3.343***	− *2.469* to ***−*** ***3.200***	− *2.559* to − *2.695*	− *1.499* to ***−*** ***3.195***	***−*** ***3.358*** to ***−*** ***3.588***
NTC C_q_	*35.44* to *37.60*	***N/A***	***N/A***	*Peaks* ^c^	*38.05*	*33.04* to *39.44*	***N/A***	*35.04*	*26.39* to *39.27*	*39.24*

*Bold italic* acceptable result;* italics* unacceptable result

^a^For an PCR efficiency (E) of 100%, the slope is − 3.32. A good reaction should have an efficiency between 90 and 110%, which corresponds to a slope between − 3.58 and − 3.10

^b^The linearity of the assay (R^2^), where R^2^ < 0.980 unacceptable; R^2^ ≥ 0.980 acceptable; R^2^ > 0.990 expected; R^2^ > 0.995 exceptional

^c^The peaks had shoulders, i.e. indication of secondary structures within the molecule

**Table 9:** Summarised C_q_ results for AuNP-interference assessment of the universal RNA standard spiked at the amplification step (part 2), by using CFX Manager™ software

Reference gene C_q_^a^	18S	ACTB	GAPDH	GUSB	HPRT1	HSP90	PPI	SDH	TBP	YWHAZ
0%AuNP	13.36–14.39	16.80–18.12	16.93–18.25	23.03–23.75	22.16–23.41	19.14–20.12	18.29–19.05	22.45–24.64	24.12–25.52	20.47–21.34
25%AuNP	***14.20***–*14.95*	***17.67***–***18.15***	***17.44***–***18.69***	***23.66***–*24.36*	***23.10***–*24.30*	***19.33***–***20.37***	*19.97*–*21.04*	***22.89***–***24.22***	***24.86***–***25.50***	***21.15***–***21.73***

Due to restrictions on the final reaction volume per qPCR, only a 25% vol/vol AuNP spike could be accommodated

*Bold italics* acceptable result;* italics* unacceptable result;* plain text* base-line result, e.g. for untreated/0%AuNP samples

^a^A C_q_ change of 0.2 is acceptable, but > 0.5 is unacceptable

## Discussion

Although many different effects of AuNPs in PCR have been published, it has not been applied to the field of Toxicology or nano-toxicology studies, i.e. where data can be misinterpreted as being non/toxic simply due to assay interference [20]. However, this current report is the first, step-for-step, detailed description indicating how the intracellular residual presence of AuNPs in a sample can alter the RT-qPCR results and, thus, the interpretation of the toxicity of AuNPs based on the assay. This work, therefore, promotes awareness regarding the effects of residual intracellular ENMs (i.e. those remaining in biological samples after cell lysis followed by traditional isolation/purification procedures). The work explains why scientists who conduct molecular biology work related to nanomaterials must be aware that the traditional reasoning for assessing the toxicity of larger chemicals does not apply since those molecules may not be metabolised, whereas metal or metal oxide ENMs may be bio-durable and remain within the cell for long time periods.

### Preliminary study

The in silico analyses assessed the design of the primers. This analysis predicted that the primers for *18S*, *GUSB*, *HSP90*, *SDH* and *YWHAZ* genes would not form secondary structures, i.e. these primers would amplify and produce acceptable PCR amplicons (see Additional file 1). Thereafter, a preliminary test was performed using SYBR Green in order to screen these primers. This part of the study was completed by analysing the RNA obtained from BEAS-2B cells that had been treated with AuNPs (see Additional file 2).

Furthermore, the manual assessment determined whether or not AuNPs could interfere with the assay. Assessment of the amplification of the reference genes, as influenced by AuNPs, included use of the CFX Manager software to obtain the PCR efficiency, the linearity of the PCR assay as well as the gradient or slope obtained from the standard curve. The melt curves of the final PCR amplicon were also analysed. The preliminary study did confirm that the primers could amplify the required amplicons in accordance with MIQE guidelines [6]. However, possible assay interference caused by AuNPs may have occurred and should not be ignored.

Admittedly, ENMs may have different coating and capping agents, in addition to forming a protein corona when incubated in different culture media, which may alter their intracellular uptake and toxicity [34]. It is only those that internalize and are bio-durable that should be considered when assessing their interference upon cell lysis during assay procedures. The proposed interactions that may have caused this interference are discussed in detail below. Hence, it is generally understood that surface properties of ENMs will affect intracellular uptake and subsequent gene expression within intact cells. In contrast, what is not generally understood is the consequence of internalised ENMs that are already present within the cells before and during lysis. This aspect has not been widely investigated. Therefore, it was decided to test the primers and validate the RT-qPCR assay. Appropriate control samples were included to represent samples with “non-functionalised ENMs” and “no ENMs” so as to focus on the interference specific to the RT-qPCR assay, instead of the biological interaction at the cell’s intact surface membrane.

### Assessment of reverse transcription

The universal RNA standard, obtained from ten cell lines, was spiked with AuNPs, before reverse transcription, in order to generate cDNA. This method assesses the effect that AuNPs could have on RT-qPCR if AuNPs were internalised in cells, i.e. it mimics the situation where AuNPs may interact with cytosolic cellular content of mRNA, which is usually exposed during translation. Thus, the treatment has biological significance because it mimics any possible interference of residual ENMs present in biological samples, which would co-precipitate with isolated RNA intended for analyses during toxicity exposure assessment studies.

Initial observations of only the amplification plots obtained from the CFX Manager software indicated a change in the profiles for some of the reference genes, i.e. before and after deliberate addition of AuNPs (see Fig. 2; Additional file 3). The fluorescence reading was quenched and, thus, the C_q_ increased. Finally, the qPCR software and statistical programs assessed the accuracy and stability of the gene expression. Both traditional analyses, as well as, manual assessments were performed on the results. In addition, the results were analysed based on the point at which the AuNPs were added to the reaction, i.e. either spiked at the reverse transcription step (part 1), or, spiked at the amplification step only (part 2).

The analyses relied on traditional software packages that are specific for qPCR assays in order to perform relevant statistical comparisons (see Additional file 4). To summarise, when comparing results obtained from the universal RNA that had been spiked with AuNPs at the reverse transcription step (part 1), all the statistical analysis programs found that the same reference genes exhibited the highest stability. These included *HSP90*, *SDH* and *YWHAZ*. In addition, stable combinations of reference genes were also identified, i.e. *GUSB* with *HSP90*. When working with ENMs, it is recommended to not solely rely on traditional qPCR software analysis programs, but to also do manual assessments, e.g. determine PCR efficiency variations between treatments, as well as changes in the dissociation assay (melts) of the different products formed (see Additional file 5). Due to the changes in the Cq, *GAPDH*, *GUSB*, *HPRT1* or *SDH* could possibly be suitable reference genes. However, the melt peak analysis of all the products formed did not identify any significant differences (data not shown).

### Assessment of DNA amplification

The second part of this assessment analysed the effect of AuNPs on only the DNA amplification step (part 2; see Additional file 6). This method assesses the effect that AuNPs could have on RT-qPCR when AuNPs are internalised in cells, i.e. it mimics the situation where AuNPs may interact with single-strand DNA, which is usually exposed during cell replication. These experiments were therefore required for continuity in order to compare the interference of AuNPs throughout each point in the experiment, i.e. continuation of the detailed step-for-step assessment.

The cDNA, obtained from the universal RNA standard, was spiked with AuNPs after reverse transcription was completed, in order to amplify the cDNA under PCR conditions with deliberate AuNP interference. This treatment would mimic those samples where DNA was isolated from AuNP-treated samples and amplified by qPCR, e.g. assessment of methylation studies, SNP and mutation analyses or genomic DNA etc. This also implies that testing only the amplification step (but nothing prior), is not sufficient to verify qPCR assays for genotoxic studies related to ENM exposure assessments, where the starting material might be contaminated with residual ENMs. In general, when comparing results obtained from the universal RNA that had been spiked with AuNPs at the amplification step (part 2), the combined results showed that only three reference genes exhibited the highest stability, i.e. *GUSB*, *HSP90* and *YWHAZ*.

Further analyses deemed *GAPDH* and *GUSB* to be inappropriate as reference genes. Separation of the PCR amplicons via electrophoresis identified the formation of multiple products for some of the replicates of *GUSB*, which was subsequently disqualified as a suitable reference gene. In addition, although *GAPDH* is a popular reference gene in many studies, it does have limitations [35–37]. Firstly, *GAPDH* plays a role in glycolysis and as such, may result in variable expression in different tissues or disease states. Secondly, some *GAPDH* pseudogenes are expressed, where primers will detect the presence of both the pseudogenes and the cDNA of the active transcript. Lastly, considering that the human genome may contain up to 60 pseudogenes for *GAPDH*, DNase treatment may not always degrade the entire genomic DNA in which these sequences reside. It was, thus, proposed that only *YWHAZ* and/or *HSP90* be used as reference genes. In addition, future studies should focus on the clustering feature available in the Precision Melt Analysis™ software, i.e. HRM. In fact, a few HRM shifts were identified by an in-depth analysis of the melt profiles, where readings were taken every 0.2 °C (data not shown). Thus, these genes have been identified as targets for developing a “diagnostic tool” and are currently being investigated further.

### Proposed interference based on comparisons to previous studies

There is a continuing concern with assay interference caused by ENMs that has not been addressed, which has a serious implication for nano-toxicity testing in assays using PCR-based techniques. When ENMs enter cells, the residual amount of intracellular ENM remaining in a sample could alter the PCR assay and, thus, generate false readings for gene expression based exposure-studies. The results reported herein indicate that BEAS-2B cells could internalise the AuNPs, where the AuNPs would have access to cellular content during cell lysis. In addition, the deliberate addition of AuNPs into a sample, at either the reverse transcription or PCR amplification stage of the assay would interact with these macromolecules and/or assay reagents to cause assay interference.

The section below serves to highlight the main points to consider for cytotoxic and genotoxic studies, by highlighting common observations within all these different reports.

#### Interference with the dye used for detection

When a fluorophore is in close proximity to a metal nanoparticle displaying plasmon resonance, its fluorescence emission may change [38]. These authors identified factors that affect the fluorescence of a fluorophore when it is near AuNPs, i.e. the particle size, coatings, as well as, the wavelengths of the incident light and emitted light. Hence, they concluded that fluorescence may be enhanced or quenched by changing the distance between the fluorophore and the AuNP. In fact, Rosa and colleagues developed a tool for dealing with this modulation between fluorescence enhancement or quenching when close to AuNPs [39]. The authors concluded that this was a more accurate method for determining fluorescence emission near AuNPs. In other words, one can correct for the spectral response when fluorophores are conjugated to AuNPs, which is relevant to nano-diagnostics that rely on quantification assays.

The fluorescence detected in qPCR is generated by a DNA-binding dye. This implies that the conformational structure of DNA requires and ensures a specific spatial interaction with the dye. A study was found to be similar in design to that reported herein, i.e. a presentation by Prado and colleagues at the qPCR-NGS 2013 Symposium held in Germany [40]. The authors noted that the amplification plot of SYBR Green was affected by the addition of increasing concentrations of Fe_3_O_4_ NPs. In contrast, in our study reported herein, AuNPs that were 14 nm in size and citrate stabilised, were used as the treatment. It should also be emphasised that the dye used by Prado and colleagues was SYBR Green. This is a “relocating” dye, where even though the SYBR dye melts off at a dissociated part of the DNA strands, it has the potential to re-attach at another point in the same DNA strand that has not melted. Therefore, SYBR Green has the potential to generate false readings, where a study by Yang and colleagues reported partial quenching of the fluorescence of SYBR Green in qPCR caused by AuNPs [41]. The authors explained that since SYBR Green only becomes fluorescent after binding to the minor groove of dsDNA, it implies that AuNPs must first bind to dsDNA before it can quench the fluorescence in a qPCR assay. This is, therefore, a non-specific interaction between the ENM and the DNA, which could explain conflicting genotoxicity reports published to date. Irrespective of the mechanism, by using amplification profiles and melt analyses, changes in a PCR have been attributed to AuNPs that cause fluorescence quenching and DNA duplex destabilisation [42]. These authors concluded that a thorough evaluation and validation of the impact of AuNPs on any qPCR assay should be undertaken and further serves to emphasise the importance of our study presented herein. The possibility that SYBR Green could further be compromised by interacting with ENMs was again proposed recently [24]. Shaat and colleagues investigated AuNPs as carriers for delivering siRNA when they detected possible assay interference. However, they concluded that their results did not change when they repeated the experiment using an end-point RT-PCR assay in the absence of the SYBR Green dye, i.e. the interference was not specifically due to the interaction with the dye. Hence, they referred to our own previous study where we have reported assay interference during an RNA isolation procedure [1]. Again, the importance of the effect of residual intracellular ENMs in the elucidation of genotoxicity studies has become a recurring theme in the latest publications.

EVA Green was used as the dye in our study reported herein. This is a “saturated” dye, which binds at each position in the DNA strands and reduces false readings, i.e. it is a better option compared to SYBR Green. Even so, as mentioned above, we observed that the addition of AuNPs reacted with the PCR enzyme cocktail and changed the colour to a clear solution. It should be noted that the super-mix contains the Sso7d-fusion polymerase, which employs an antibody-mediated hot-start feature in order to sequester the enzymatic activity prior to the initial PCR denaturation step. Once the antibodies denature irreversibly during the heat activation step, the DNA polymerase is released and is fully active. Therefore, it is proposed that in our study the AuNPs have the potential to interfere with the PCR assay by interacting with the buffer components, the hot-start antibody or the polymerase enzyme, but not necessarily the dye.

#### Influence of thermal conductivity of ENMs

Gold colloids can form a highly ordered liquid layer that can lead to higher thermal conductivity [43]. Therefore, when DNA is in close proximity to gold colloids it can induce a fast heat transfer and, thus, enhance PCR locally around that DNA [44]. Li and colleagues proved that most of the primer and DNA did not bind the gold colloid, even though some reports propose that ssDNA replaces the citrate ions to bind to the gold colloids. Li and colleagues went further and analysed PCR reactions using both traditional DNA polymerase (*Taq*), as well as, SYBR Green enzyme cocktails, with citrate-capped AuNPs 12.7 ± 0.8 nm in order to investigate the influence on PCR efficiency. They determined the effective concentration of gold colloid to be 0.7 nM. Our study reported herein used a FC 0.72 nM for the 25% AuNP-spiked samples. Therefore, the lowest concentration used in our study was sufficient to investigate possible assay interference. Li and colleagues proved that the thermal conductivity of the AuNPs played a significant role in shortening the time required for heat dispersion or equilibrium and, thus, increased the efficiency of the traditional end-point PCR reaction tested. When Li and colleagues tested a qPCR reaction, similar increased PCR efficiencies were obtained, but the C_q_ values shifted by 11 cycles compared to the positive control. The authors interpreted this finding as increased PCR efficiency. However, it may actually be a sign of ENM-induced assay interference in a PCR.

#### Interactions with components of the PCR enzyme cocktail mix

A recent report assessed the effects of specifically AuNPs on PCR [19]. Briefly, the authors reported that an excess of ENMs would inhibit PCR by either adsorbing to the polymerase, to Mg^2+^, to oligo-nucleotide primers or to the cDNA/DNA templates. The study by Bai and colleagues differs to that reported herein since lamda (λ) DNA and amino-modified silica-coated magnetic NPs were used. Instead in our study we used AuNPs and RNA from ten cell lines so that the study would have biological significance, i.e. the results obtained mimic those of genotoxic analyses after AuNP exposure. Furthermore, the authors disproved the theory that ENMs inhibit non-specific amplification by false priming. Rather, they showed that it was a concentration-dependent phenomenon, i.e. low concentrations of NPs inhibit amplification of long amplicons, and, increased amounts of NPs inhibit amplification of short amplicons. This means that for our study, where non-cytotoxic amounts or < 50% vol/vol of AuNPs were used, the low concentration of NPs might only interfere with the longer amplicons, e.g. 18S, *ACTB*, *GUSB*, *PPI* and TBP (see Table 1). This is currently being investigated further with the development of the proposed diagnostic tool.

Furthermore, Li and colleagues reported that enhancement of the PCR efficiency depended on the DNA polymerase used [44]. These enzymes may have different proof-reading abilities, which would influence the binding and amplification of the DNA strand, where altered or damaged templates would have different amplification efficiencies. Hence, the findings by Li and colleagues support our hypothesis that AuNPs have the potential to interfere with the hot-start antibody or the polymerase enzyme, as mentioned above. The complex interaction between AuNPs and the DNA polymerase (Taq) was also reported elsewhere [41].

#### Binding of nucleic acids relevant to DNA amplification

The composition of the DNA sequence may also play a role in the degree of assay interference observed. For example, one study found that the dA mononucleotide had a stronger affinity for 5 nm AuNPs, most probably due to the freely accessible amine group [45]. Therefore, mRNA that contains a characteristic poly-A tail at the 3′-end of the molecule might be more susceptible to interference caused by gold-based ENMs. This means that for some genotoxicity studies, which may use mRNA as a starting material, the results could be influenced by this affinity for small AuNPs. However, the poly-nucleotides tested by Yang and colleagues were more rigid due to the phosphate backbone of DNA, which resulted in altered affinities to the AuNPs (see further discussion below regarding the effect of charge). In addition, they pointed out that the dsDNA dissociation caused by AuNPs was strongly dependent on the particle size (see further discussion below regarding the effect of size). These findings imply that primers that are designed to amplify A-rich regions in the DNA sequence may be problematic. Also, amplification of the 3′-end of gene may be challenging, especially when using mRNA as the starting material.

Another study went further and explored the effect of charge on DNA binding [46]. Initially, they found that the small uncharged AuNPs were able to bind the minor groove in DNA, but did not damage the structural integrity of the helix or disrupt Watson–Crick pairing. Thereafter, they used charged AuNPs that were 1.4 nm in diameter and found that at high concentrations it could bind DNA via electrostatic interactions from the cationic ligands, which then lead to bending and strand separation. In contrast, our study used larger citrate-stabilised AuNPs (14 nm in size), with a negative net charge. Hence, interaction between our large negatively charged AuNPs and the negatively charged phosphate backbone of nucleic acids found in template RNA/cDNA is not very probable. In fact, a thiol group is usually required in modified dsDNA terminals in order to enable covalent bonding with the metal surface [45]. Rather, the proposed interaction between the AuNPs and the polymerase enzyme or the Mg^2+^ in the buffer, as well as, the effect caused by a higher thermal conductivity around the DNA are more plausible reasons for any variations observed in the results, i.e. assay interference.

The effect of ENM size has also been investigated [47]. Although 5, 10 and 20 nm sized particles were tested, the authors reported that AuNPs could inhibit a PCR reaction. However, that this was due to the concentration of the AuNPs used. Specifically, the larger sizes caused PCR inhibition at lower concentrations, in comparison to smaller sizes. The “threshold” concentrations were determined to be 5.5, 1.1 and 0.24 nM for the 5, 10 and 20 nm sized particles, respectively. They proposed that their observations could be due to AuNPs that bind to the polymerase. They also went further and explained that all their results showed that the total surface area of the AuNPs altered the PCR product yield, i.e. the size influences the surface area, which then prompts interactions with the PCR components. Specifically, a higher concentration of the smaller sized AuNPs was required to cause inhibition of the PCR. In addition, when the concentration was adjusted to make sure that total surface area in the PCR reaction was the same for the 5, 10 and 20 nm sized particles, it resulted in equal inhibition of the PCR. This could explain why different studies show different degrees of PCR inhibition, i.e. the different studies do not take into account the entire surface area of the ENM that is able to interact with the PCR components. The only problem with their findings is that these statements were based on observations made on amplification occurring between 35 and 50 cycles. It is well documented that primer-dimers will form after 35 cycles of amplification. Hence, most qPCR assays do not exceed 40 cycles to avoid quantification of a non-target amplicon. However, the authors declared that a 119 bp *invA* amplicon was observed after electrophoresis [47].

Selective adsorption of single-stranded oligo-nucleotides onto the surface of AuNPs, via electrostatic interactions, has been reported [48]. However, dsDNA did not adsorb and this difference between the two states of DNA was used to detect changes in oligo-nucleotide lengths in the presence of a dye, i.e. parts of hybridised chains would have electrostatic properties of ssDNA and other parts would have properties of dsDNA (due to contact between the dye and gold). This observation was further developed where the authors reported on probes, consisting of small oligo-nucleotides, which are able to bind to RNA. The fluorescence of unbound probes was quenched by the presence of AuNPs. This difference in (quenched) fluorescence was used to indicate that probes (which were designed for a particular target RNA), did actually bind in the sample, i.e. identification and confirmation of the target sequence [49]. The structure of the RNA was determined based on which of these probes hybridised, i.e. mismatched sequences would result in weaker binding. Furthermore, in a different study performed by Yang et al. of a qPCR assay with SYBR Green, they reported that smaller AuNPs had a higher binding affinity for single-stranded (ss) DNA, i.e. the nitrogen atoms in the amines of DNA underwent covalent interaction with the gold atoms and disrupted the hydrogen bonds formed between complimentary oligonucleotides [41]. Yang and colleagues went further to propose a non-specific interaction between small AuNPs and the phosphate backbone, which may disrupt geometric hybridisation and, therefore, cause partial dissociation of dsDNA. These findings correlate to the Railsback study mentioned above, as well as, our own previous findings from the study of the interaction of AuNPs with single-stranded RNA [1]. It should be noted here that different kinds of interactions are being referred to, i.e. the non-specific binding refers to uncharged ENMs that are able to fit in the minor groove of a dsDNA complex, whereas the charged ENMs are able to cause electrostatic interactions that can bend and separate the DNA stand and, lastly, the covalent interactions rely on sharing of electrons, e.g. thiol group with a metal group. In addition, the reports discussed above imply that when AuNPs dissociate dsDNA, it could lead to lower melting point of the final PCR products because the structure of the DNA-duplex is distorted, similar to the mechanism discussed above, where increased heat transfer caused by a gold colloid could lead to easier DNA duplex strand separation for PCR amplification, i.e. lower C_q_ values [44]. In other words, the qPCR melt peak would shift to a different/lower temperature and the HRM profile would also change for qPCR-based results. Hence, an analysis of HRM profiles is being developed further by us as diagnostic tool.

#### Limitations of assay

Another factor to consider is the limit of detection of the qPCR assay itself. According to the MIQE guidelines [6], a lower C_q_ is correlated with higher target expression in a sample. In other words, the C_q_ values are inversely proportional to the amount of target nucleic acid present in the sample, where the lower the C_q_, the greater the amount of target present. In addition, the C_q_ value must be compared to the non-template control (NTC) as well as to either, (a) another C_q_ value in another sample to calculate the relative expression, or, (b) a standard curve where known amounts of target are analysed leading to absolute quantification. Without this normalisation step, the C_q_ values should not be used to draw any conclusions. For example, a recent study noted that high concentrations of AuNPs increased C_q_ values and concluded that it was an inhibitory effect [50]. Although the deliberate addition of AuNPs did create a false estimation of the initial DNA, the authors interpreted this change as increased sensitivity. It is a misconception to link lower C_q_ values with increased sensitivity [51]. A true indicator of sensitivity is actually the limit of detection. The results reported by Gurjar and colleagues should rather be viewed as an example of how 33.2 and 51.51 nm sized AuNPs interfered with the SYBR Green based qPCR assay. Upon closer inspection, it appears that the assay interference caused by the AuNPs altered the melt profiles of the PCR amplicons, where the melt peak shape and peak amplitude was changed, i.e. their analyses of the final PCR products generated indicated that the products were not the same.

In another recent study, upconversion nanoparticles (UCNPs) were reported to improve the specificity of the PCR [52]. This was an end-point assay, not a “real-time” qPCR assay and the results were interpreted as increased specificity because the number of additional amplicons decreased as the intensity of the main PCR amplicon also decreased, i.e. total PCR inhibition. The gene target in their PCR was a 120 bp *5S rRNA* repeat. These *5S RNA* repeats are present as multiple copies of target sequence in the genome. The *5S rRNA* genes are organised as tandem repeated clusters and the gene copies range from 100 to 300,000. Therefore, depending on the stringency of the primer design, a primer pair may amplify many similar copies, which will appear as many different bands on an agarose gel. Hwang and colleagues observed multiple products in the control sample that did not contain UCNPs, which was most probably due to the primer design. Subsequently, when the PCR was inhibited (including the main amplicon), all these non-specific products were also inhibited. In contrast, to unambiguously show an increase in PCR specificity, one would first need an optimised PCR reaction without non-specific products and then prove that the addition of a specific ENM altered the rate of amplification or the proof-reading ability and resulted in the production of a superior PCR product. Hwang and colleagues did emphasise a decrease in PCR amplification as caused by the UCNPs and, although it may not be an example of increased PCR specificity, it most definitely is another example of assay interference. Again, this recurring theme in the latest publications of how residual intracellular ENMs are able to alter assays needs clarification for the correct interpretation of genotoxicity studies.

### Recommendations

Although the reports of previous studies mentioned above are ambiguous, continued analyses of studies with the deliberate addition of AuNPs can be used to predict assay interference, i.e. deliberately change the mechanism of a PCR reaction to mimic conditions where intracellular ENMs may unintentionally change the dynamics of the PCR components. This applies to all situations where ENMs, irrespective of the functionalised surface modification, are internalised into cells. It is important because in such cases where intracellular ENMs remain in PCR-related samples, the results would incorrectly be interpreted as an indicator of gene expression and be used to determine ENM toxicity in the sample. Where one suspects residual intracellular ENM contamination of starting material (especially gold), the following should be avoided:

1. Design of primers to amplify near A-rich regions in the DNA sequence.
2. Amplification of the 3′-end (near the poly-A tail) of gene when using mRNA as the starting material.

Other recommendations include the use combinations of genes (e.g. *HSP90* or *YWHAZ*), for improved gene expression normalisation. It is further recommended that RNA standards, which have been spiked with known amounts of the ENM (e.g. AuNPs), should be run in conjunction with the unknown RNA samples. Thus, one could determine the degree of error with the associated compensation required for assays influenced by ENMs. This implies that multiple forms of analyses are required, in order to determine the degree of error in the assay. As described herein, this can be achieved by using both traditional software programs and via manual assessments of the individual parameters.

## Conclusions

Although many different effects of AuNPs in PCR have been published, it is not being applied to the field of toxicology or nano-toxicology studies. There is a continuing problem with assay interference caused by intracellular ENMs that has not been addressed, which has a serious implication for nano-related testing in assays using PCR-based techniques. This study was performed in order to verify a qPCR procedure required for gene expression assays related to engineered nanomaterial exposure assessments. This is the first step-for-step detailed report indicating how the presence of AuNPs can alter the RT-qPCR results. A preliminary test was performed using BEAS-2B cells that had been treated with a non-cytotoxic concentration of 1 nM AuNPs, since these are the proposed conditions for all planned gene expression work yet to be performed. The in silico analyses assessed the design of the primers. The traditional qPCR software and statistical programs assessed the accuracy and stability of the gene expression. The manual assessment determined whether or not AuNPs could interfere with the assay. Although there were differences in the ranked order, all the software analysis programs found that the same group of reference genes exhibited the highest stability after 14 nm citrate-stabilised AuNP treatments. However, it was found that the AuNPs interfered with the qPCR assay, which would influence the RT efficiency, PCR efficiency and dissociation dynamics. Therefore, these analyses identified genes that were suitable as the best reference genes to be used for normalisation, e.g. *YWHAZ* and/or *HSP90*. The discussion also covered a comparison between SYBR Green and EVA Green, in addition to the influence of ENM thermal conductivity, surface interactions with ENMs, effects of ENM size and charge, as well as, the limit of detection in qPCR. This work, thus, promotes awareness regarding the effects of residual intracellular ENMs (i.e. those remaining in biological samples after traditional isolation/purification procedures). The work explains why scientists who conduct molecular biology work related to the toxicity of nanomaterials must be cautious. Lastly, this report describes steps that can be utilised to generate a suitable method for gene expression studies related to various ENMs, i.e. it can be applied to other types of ENMs in qPCR-based toxicity assays. This report proves that qPCR is flawed when used for toxicity testing of (gold) nano-particles and, as such, is novel, currently applicable and essential for understanding the varied and often contradicting reports regarding nanoparticle toxicity.

## Additional files

**Additional file 1.** In silico analysis of primers.

**Additional file 2.** Assessment of the qPCR amplification of the reference genes, as influenced by AuNP treatment of BEAS-2B human cell line.

**Additional file 3.** Dissociation dynamics melt peaks of qPCR amplification of the reference genes, from the universal RNA standard.

**Additional file 4.** Traditional software and statistical analyses.

**Additional file 5.** Manual assessment of qPCR results.

**Additional file 6.** Analyses based on the point where the assay was spiked with AuNPs.

## Abbreviations

18S: human 18S ribosomal RNA
ACTB: beta-actin
AM: arithmetic mean
AuNPs: gold nanoparticles
BEAS-2B: primary and immortalized (*BEAS 2B*) human bronchial epithelial cells
cDNA: complementary DNA
CV: coefficient of variance
C_q_: quantitative cycle, also known as cycle threshold (C_T_) and cycle crossing point (C_P_)
E: PCR efficiency
ENMs: engineered nanomaterials
EVA Green: next-generation green fluorescent DNA-binding dye
FC: final concentration
GAPDH: glyceraldehyde-3-phosphate dehydrogenase
GM: geometric mean
GUSB: beta glucuronidase
HKG: “house-keeping” gene (preferred term according to MIQE guidelines is “reference gene”)
HPRT1: hypoxanthine phosphoribosyltransferase 1
HRM: high resolution melt
HSP90: heat shock protein 90 kDa alpha (cytosolic), class B
IDT: integrated DNA technologies
LDH: lactate dehydrogenase cytotoxicity assay
M: average expression stability value used in geNorm software
MIQE: minimum information for publication of quantitative real-time PCR experiments
MTS: Muir–Torre syndrome colorimetric cell metabolic activity assay
OECD: The Organization for Economic Cooperation and Development
PPI: peptidylprolyl isomerase A (cyclophilin A)
Q: quantitative cycle value used in geNorm software
QC: quality control
r: Pearson correlation coefficient
R^2^: linearity of the PCR assay
REST: relative expression software tool
ROS: reactive oxidant species
RT-qPCR: reverse transcriptase quantitative polymerase chain reaction
SD: standard deviation
SDH: succinate dehydrogenase complex subunit A flavoprotein
siRNA: short interfering RNA
SYBR Green: asymmetrical cyanine dye used as a nucleic acid stain
Taq: DNA polymerase named after the *Thermus aquaticus* from which it was originally isolated
TBE: Tris–borate and EDTA buffer
TBP: TATA-box binding protein
THP-1: human monocytic cell line derived from an acute monocytic leukemia patient
WPMN: working party on manufactured nanomaterials
YWHAZ: tyrosine 3-monooxygenase/tryptophan 5-monooxygenase activation protein zeta polypeptide
ZnO: zinc oxide
