# Supplementary material for: The presence of residual gold nanoparticles in samples interferes with the RT-qPCR assay used for gene expression profiling
Source: J Nanobiotechnology. 2017 Oct 10;15:72. doi: 10.1186/s12951-017-0299-9 (PMC5633869; doi:10.1186/s12951-017-0299-9)

**Additional File 1: *In silico* analysis of primers**

Title: The presence of residual gold nanoparticles in samples interferes with the RT-qPCR assay used for gene expression profiling.

Authors: Natasha M Sanabria and Mary Gulumian

The presence of secondary structures in the primers can lead to poor or no yield of the product. The structures are produced by inter-molecular or intra-molecular interactions and, thus, adversely affect primer template annealing. These structures are able to reduce the availability of the primers’ nucleotides to the reaction and, therefore, affect the amplification. The *in silico* analysis was performed using the of Integrated DNA technologies (IDT) OligoAnalyzer, version 3.1 software (https://eu.idtdna.com/calc/analyzer). The target type was selected as “DNA” since the samples would be RNA that had been reverse transcribed into cDNA using the random hexamer and oligo-dT primers, i.e. before amplification with reference gene specific primers. Hairpin structures are formed by intra-molecular interaction within the primer and should be avoided **(**http://www.premierbiosoft.com/tech_notes/PCR_Primer_Design.html). Hairpin analyses were performed at 60 °C since this would be the primer annealing temperature in the qPCR reaction. The presence of hairpins at the 3' end most adversely affects the reaction. However, a 3' end hairpin with a ΔG of -2 kcal/mol (and an internal hairpin with a ΔG of -3 kcal/mol) is generally tolerable. The Gibbs Free Energy (G) is the measure of the amount of work that can be extracted from a process operating at a constant pressure, and, measures the spontaneity of the reaction. The stability of hairpin is commonly represented by its ΔG value, i.e. the energy required to break the secondary structure. A larger negative value for ΔG indicates stable and undesirable hairpins. The structure with the lowest Gibbs Free Energy (G) was the determining factor for the selection of primers for this experiment. It was predicted that the most stable primers, with the least predicted effect (e.g. hairpin loops) on the final PCR product, would include 18S, GUSB, HSP90, SDH and YWHAZ. In addition, alignments were performed and are shown below in order to indicate the position of the primer within the target sequence, as well as, the expected PCR amplicon size (see Table 1 in the main text).

**18S (NR_003286.2; NT_167214.1)**

NCBI Reference Sequence for *Homo sapiens* RNA, **18S** ribosomal 5 (RNA18S5), ribosomal RNA.

FASTA Sequence alignment with primers highlighted:

...GAGAGGGAGCCTG**AGAAACGGCTACCACATCCA**AGGAAGGCAGCAGGCGCGCAAATTACCCACTCCCGACCCGGGGAGGTAGTGACGAAAAATAACAATACAGGACTCTTTCGAGGCCCTGTAATTGGAATGAGTCCACTTTAAATCCTTTAACGAGGATCCAT**TGGAGGGCAAGTCTGGTG**CCAGCAGC...

**18S (NT_167214.1)**

Forward:

5’-AGAAACGGCTACCACATCCA-3’

ΔG(kcal.mole^-1^) = **0.41**

Reverse:

5’-CACCAGACTTGCCCTCCA-3’

ΔG(kcal.mole^-1^) = **0.81**


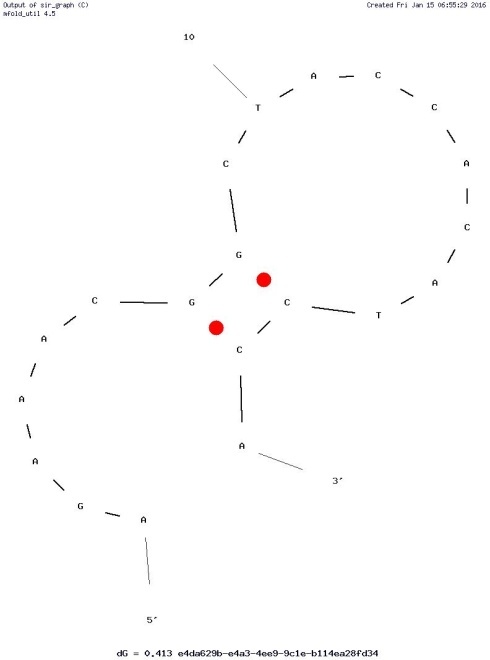


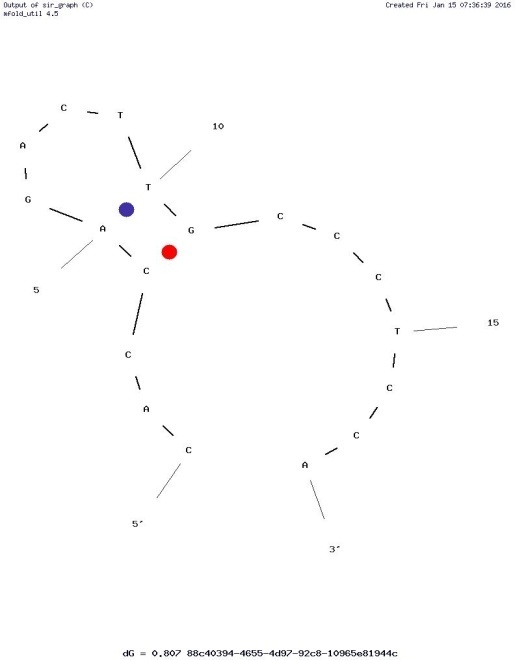


**ACTB (NM_001101)**

NCBI Reference Sequence for *Homo sapiens* **actin,** beta (ACTB), mRNA.

FASTA Sequence alignment with primers highlighted:

....TGG**AGAAAATCTGGCACCACACC**TTCTACAATGAGCTGCGTGTGGCTCCCGAGGAGCACCCCGTGCTGCTGACCGAGGCCCCCCTGAACCCCAAGGCCAACCGCGAGAAGATGACCCAGATCATGTTTGAGACCTTCAACACCCCAGCCATGTACG**TTGCTATCCAGGCTGTGCTA**TCCC.....

**ACTB (NM_001101)**

Forward:

5’-AGAAAATCTGGCACCACACC-3’

ΔG(kcal.mole^-1^) = **0.53**


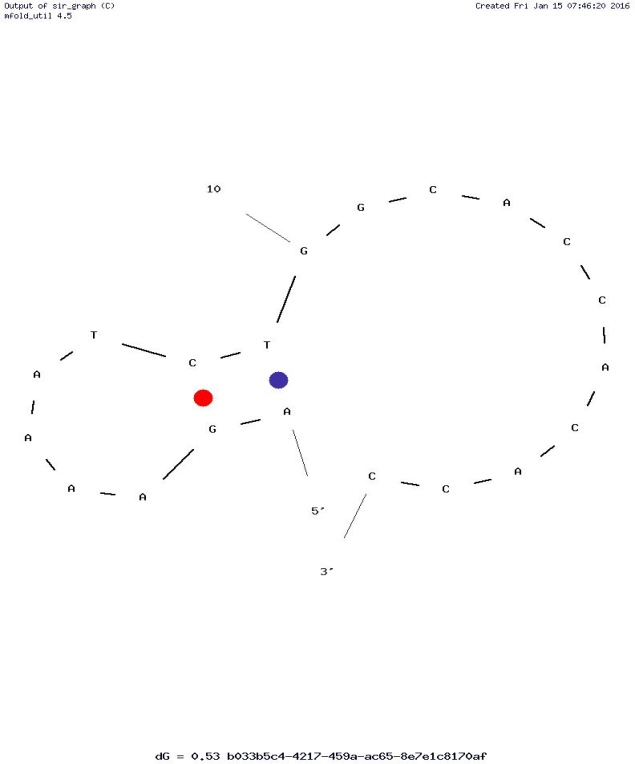


Reverse:

5’-TAGCACAGCCTGGATAGCAA-3’

ΔG(kcal.mole^-1^) = **-0.34**


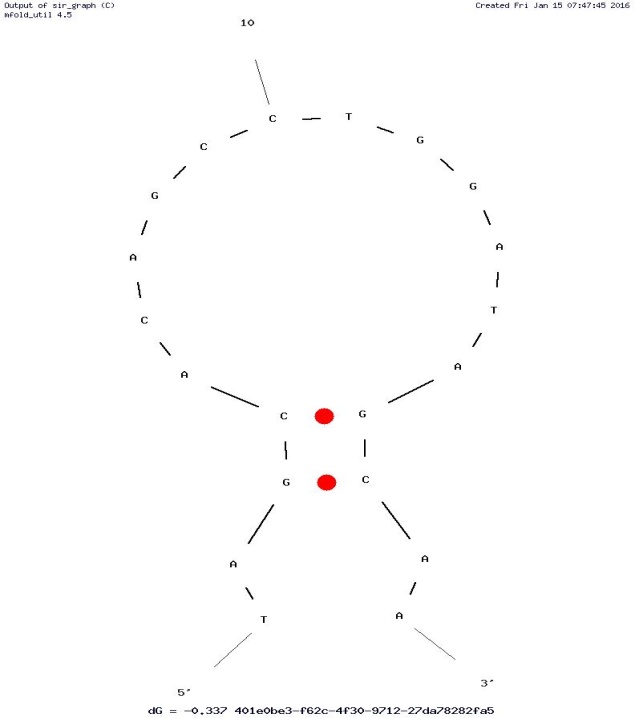


**GAPDH (NM_002046)**

NCBI Reference Sequence for *Homo sapiens* glyceraldehyde-3-phosphate dehydrogenase (**GAPDH**), transcript variant 1, mRNA.

FASTA Sequence alignment with primers highlighted:

...TTGAGCCCGCAGCCTCCCGCTTCGCTCTCTGCTCCTCCTGTT**CGACAGTCAGCCGCATCTT**CTTTTGCGTCGCCAGCCGAGCCACAT**CGCTCAGACACCATGGGG**AAGGTGAAGGTCGGAG...

**GAPDH (NM_002046)**

Forward:

5’-CGACAGTCAGCCGCATCTT-3’

ΔG(kcal.mole^-1^) = **0.81**

Reverse:

5’-CCCCATGGTGTCTGAGCG-3’

ΔG(kcal.mole^-1^) = **0.51**


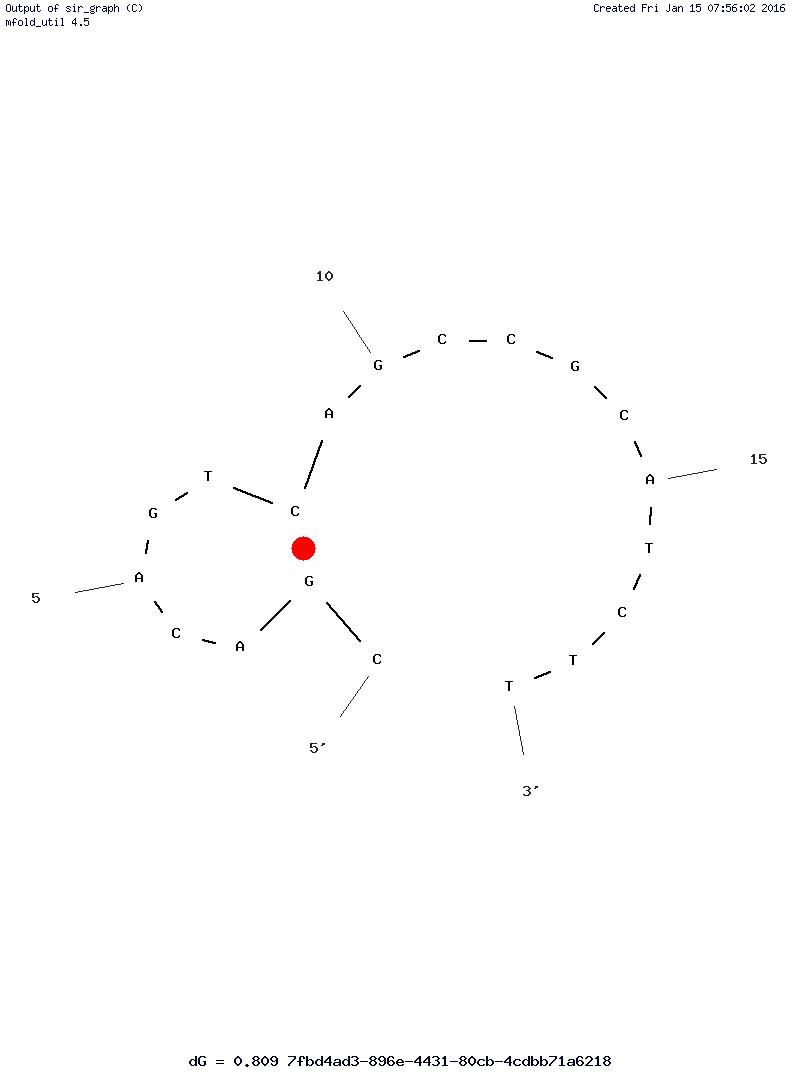


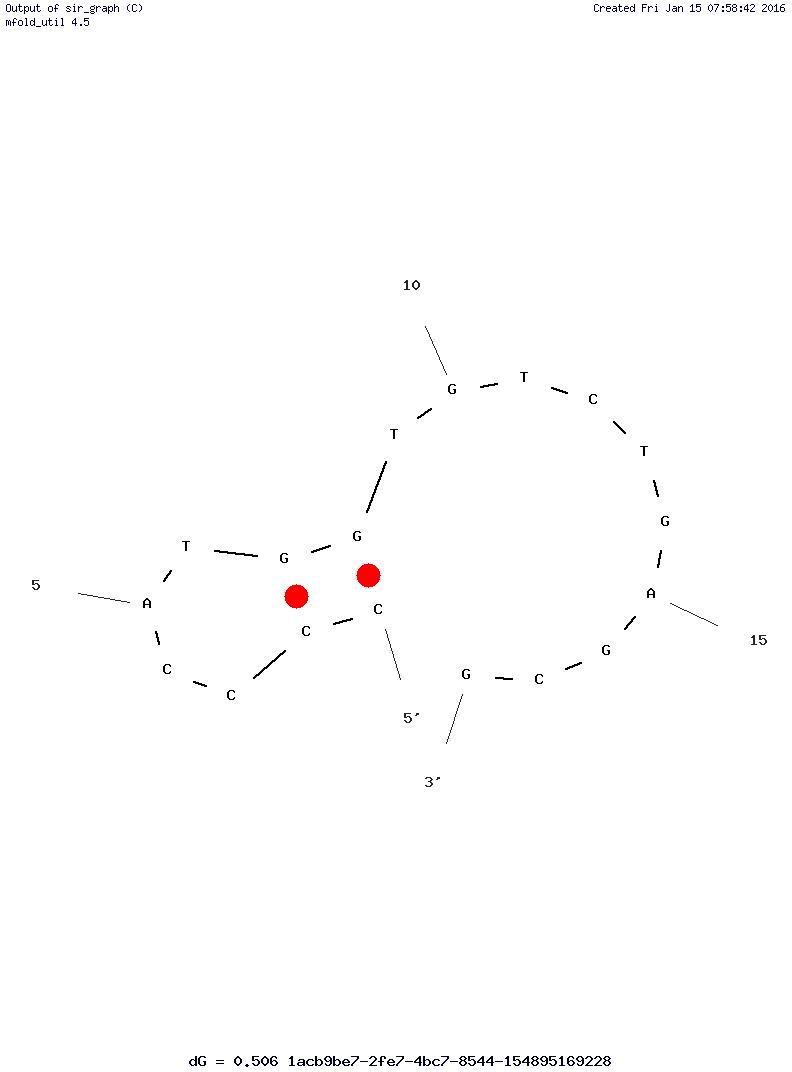


**GUSB (NM_000181)**

NCBI Reference Sequence for *Homo sapiens* glucuronidase, beta (GUSB), transcript variant 1, Mrna

FASTA Sequence alignment with primers highlighted:

...TGTCACCAAG**AGCCAGTTCCTCATCAATGG**GAAACCTTTCTATTTCCACGGTGTCAACAAGCATGAGGATGCGGACATCCGAGGGAAGGGCTTCGACTGGCCGCTGCTGGTGAAGGACTTCAACCTGCTTCGCTGGCTTGGTGCCAACGC**TTTCCGTACCAGCCACTACC**CCTATGCAGAGGAAGTGATGC....

**GUSB (NM_000181)**

Forward:

5’-AGCCAGTTCCTCATCAATGG-3’

ΔG(kcal.mole^-1^) = **-0.23**

Reverse:

5’-GGTAGTGGCTGGTACGGAAA-3’

ΔG(kcal.mole^-1^) = **-0.53**


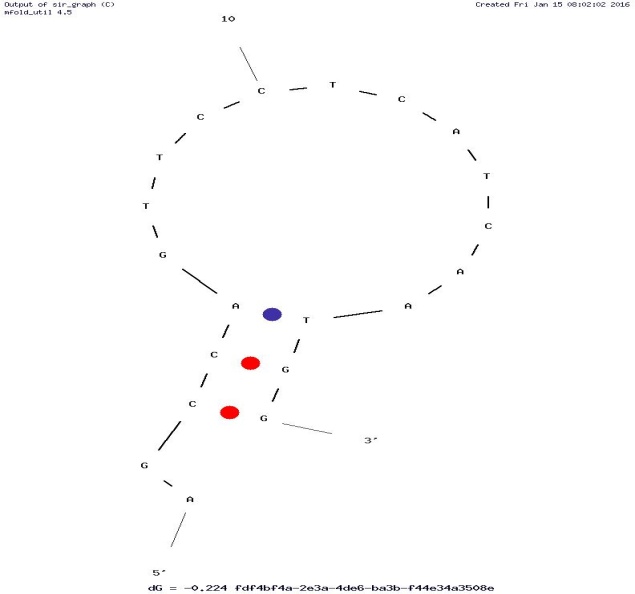


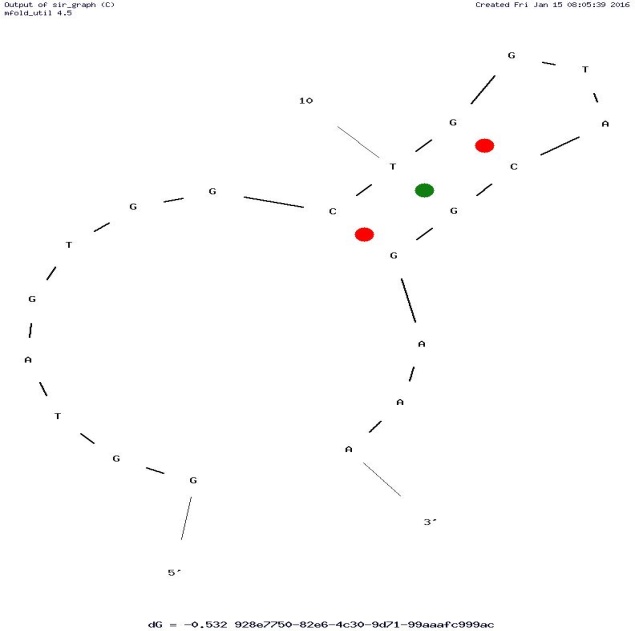


**HPRT1 (NM_000194)**

NCBI Reference Sequence for *Homo sapiens* hypoxanthine phosphoribosyltransferase 1 (**HPRT1**), mRNA.

FASTA Sequence alignment with primers highlighted:

...GTGGAAGATATAAT**TGACACTGGCAAAACAATGCA**GACTTTGCTTTCCTTGGTCAGGCAGTATAATCCAAAGATGGTCAAGGTCGCA**AGCTTGCTGGTGAAAAGGACC**CCACGAAGTGTTGGATATA...

**HPRT1 (NM_000194)**

Forward:

5’-TGACACTGGCAAAACAATGCA-3’

ΔG(kcal.mole^-1^) = **-1.9**

Reverse:

5’-GGTCCTTTTCACCAGCAAGCT-3’

ΔG(kcal.mole^-1^) = **-0.06**


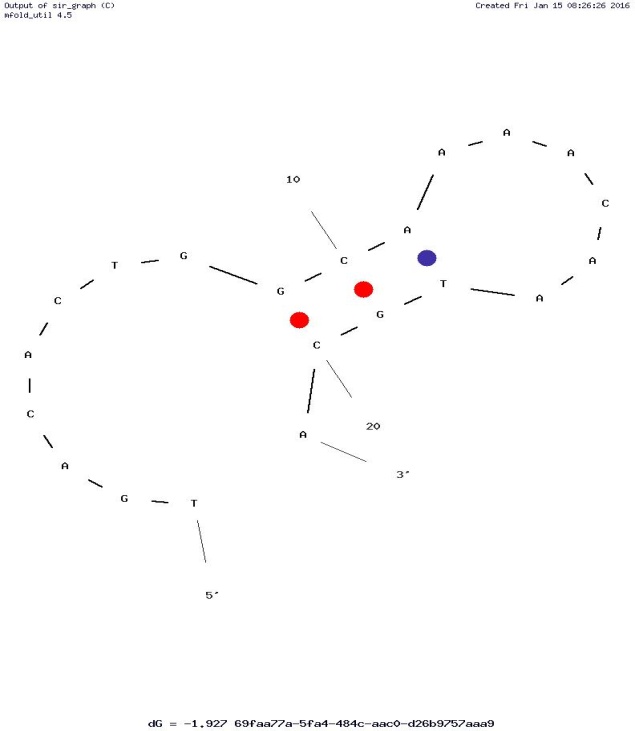


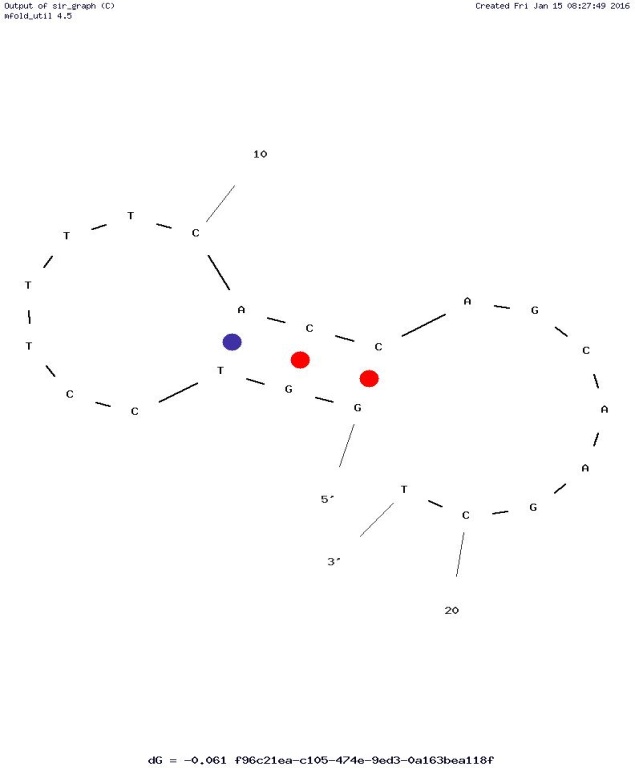


**HSP90 (NM-007355)**

NCBI Reference Sequence for *Homo sapiens* heat shock protein 90kDa alpha (cytosolic), class B member 1 (**HSP90**AB1), transcript variant 2, Mrna.

FASTA Sequence alignment with primers highlighted:

...GTCACTCCGGCGCAGTGTTGGGACTG**TCTGGGTATCGGAAAGCAAGCC**TACGTTGCTCACTATTACGTATAATCCTTTTCTTTT**CAAGATGCCTGAGGAAGTGCAC**CATGGAGAGGAGGAGGTGGAG...

**HSP90 (NM-007355)**

Forward:

5’-TCTGGGTATCGGAAAGCAAGCC-3’

ΔG(kcal.mole^-1^) = **0.54**

Reverse:

5’-GTGCACTTCCTCAGGCATCTTG-3’

ΔG(kcal.mole^-1^) = **-0.52**


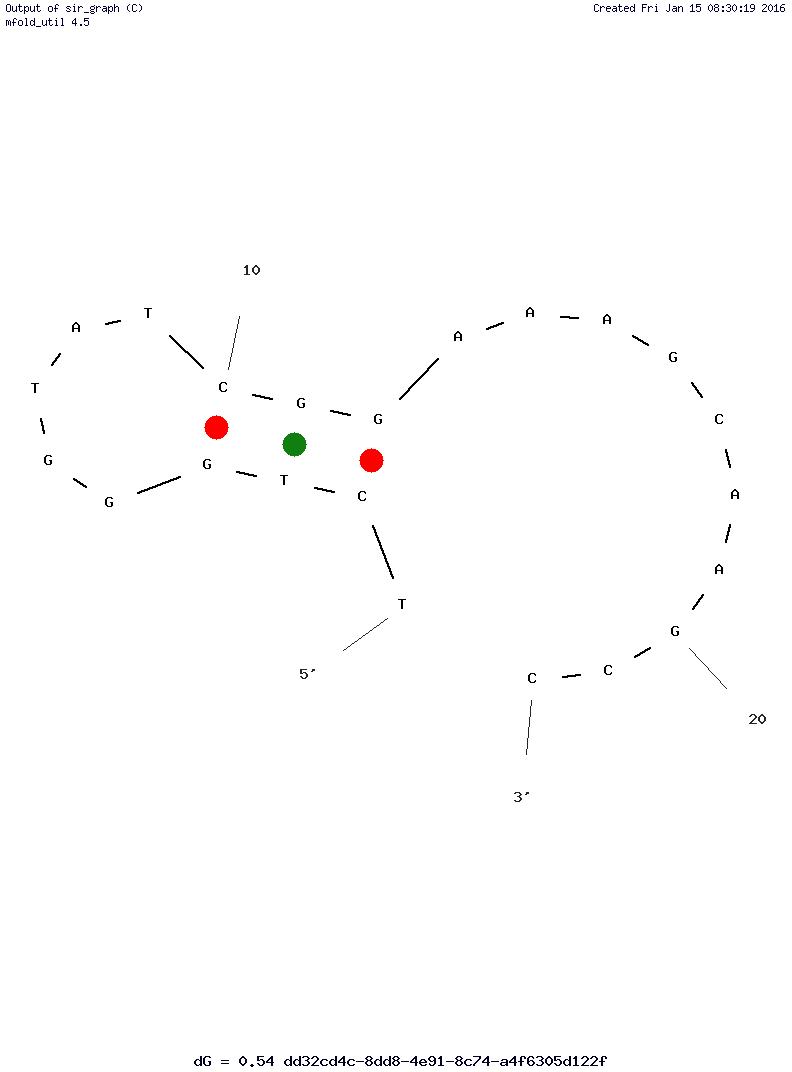


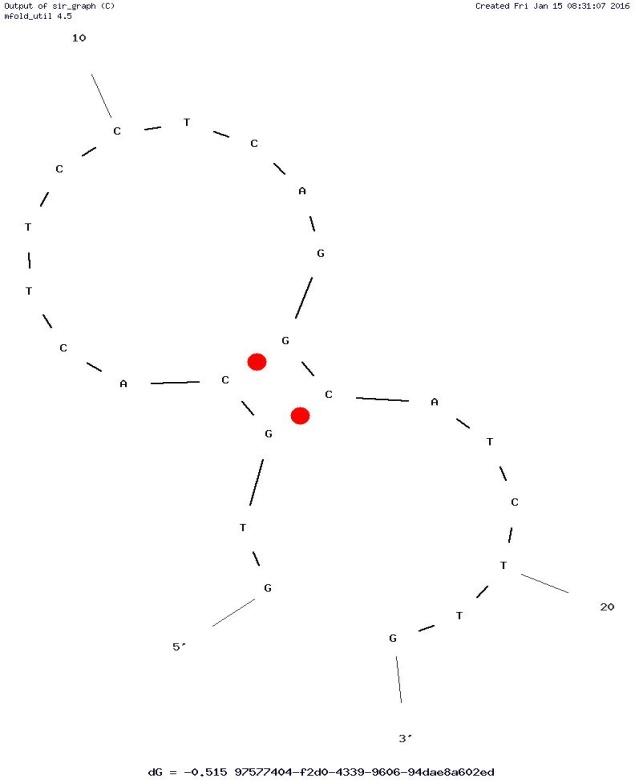


**PPI (NM_021130)**

NCBI Reference Sequence for *Homo sapiens* peptidylprolyl isomerase A (cyclophilin A) (**PPI**), transcript variant 1, mRNA.

FASTA Sequence alignment with primers highlighted:

...TTTGC**AGACAAGGTCCCAAAGAC**AGCAGAAAATTTTCGTGCTCTGAGCACTGGAGAGAAAGGATTTGGTTATAAGGGTTCCTGCTTTCACAGAATTATTCCAGGG**TTTATGTGTCAGGGTGGT**GACTT..

**PPI (NM_021130)**

Forward:

5’-AGACAAGGTCCCAAAGAC-3’

ΔG(kcal.mole^-1^) = **-0.83**

Reverse:

5’-ACCACCCTGACACATAAA-3’

ΔG(kcal.mole^-1^) = **0.67**


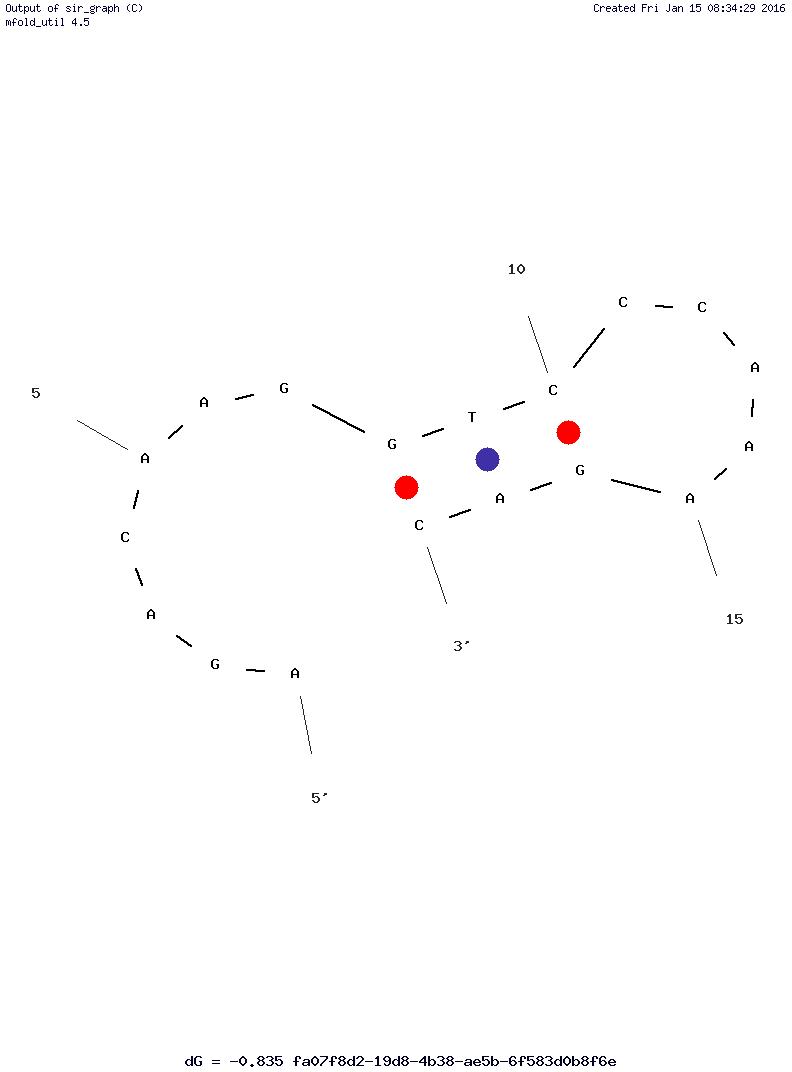


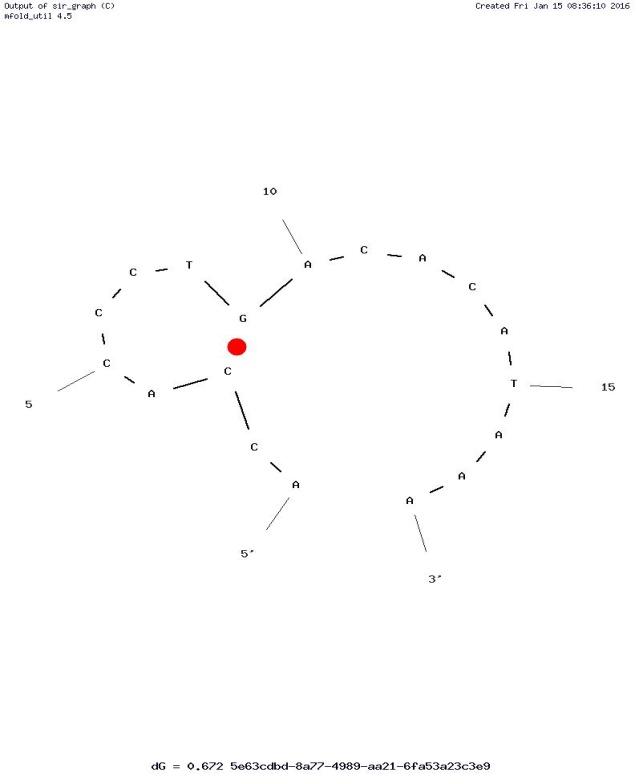


**SDH (NM004168)**

NCBI Reference Sequence for *Homo sapiens* succinate dehydrogenase complex, subunit A, flavoprotein (Fp) (**SDH**), transcript variant 1, mRNA.

FASTA Sequence alignment with primers highlighted:

...GAGGTTTTCACTTCACTGTTGA**TGGGAACAAGAGGGCATCTG**CTAAAGTTTCAGATTCCATTTCTGCTCAGTATCCAGTAGTGGAT**CATGAATTTGATGCAGTGGTGG**TAGGCGCTGGAGGGGCAG...

**SDH (NM004168)**

Forward:

5’-TGGGAACAAGAGGGCATCTG-3’

ΔG(kcal.mole^-1^) = **-0.52**

Reverse:

5’-CCACCACTGCATCAAATTCATG-3’

ΔG(kcal.mole^-1^) = **1.01**


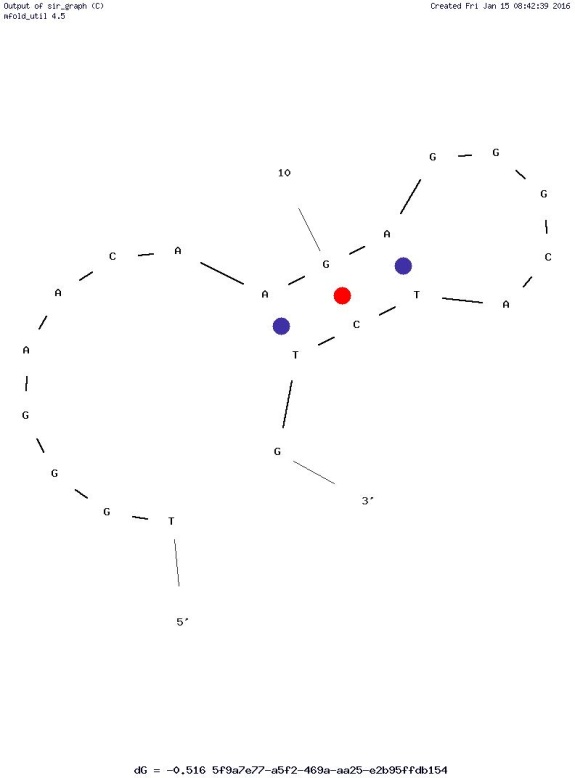

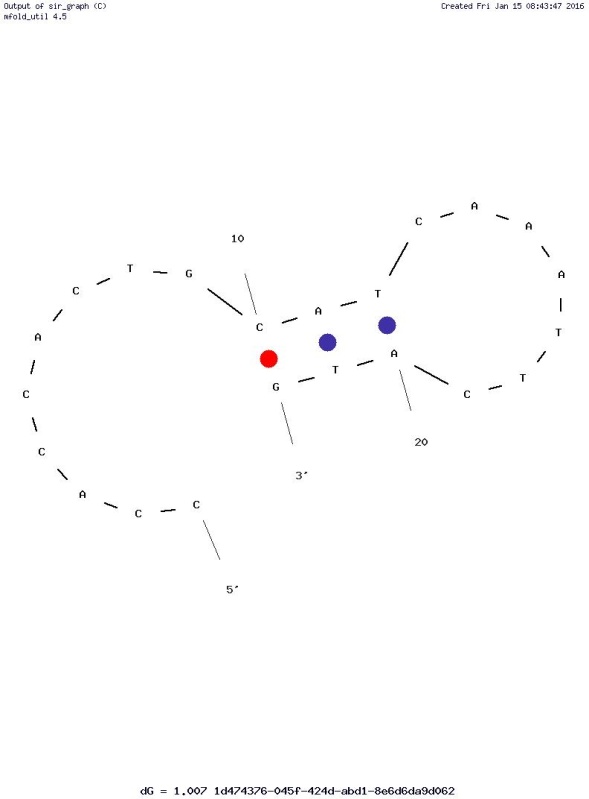


**TBP (NM_003194)**

NCBI Reference Sequence for *Homo sapiens* TATA box binding protein (**TBP**), transcript variant 1, mRNA.

FASTA Sequence alignment with primers highlighted:

...CTGGGAAAATGGTG**TGCACAGGAGCCAAGAGTGAA**GAACAGTCCAGACTGGCAGCAAGAAAATATGCTAGAGTTGTACAGAAGTTGGGTTTTCCAGCTAAGTTCTTGGACTTCAAGATTCAGAATA**TGGTGGGGAGCTGTGATGTG**AAGTTTCCTATAAGGTTAGAAGGCCTT...

**TBP (NM_003194)**

Forward:

5’-TGCACAGGAGCCAAGAGTGAA-3’

ΔG(kcal.mole^-1^) = **-1.14**

Reverse:

5’-CACATCACAGCTCCCCACCA-3’

ΔG(kcal.mole^-1^)

No (hairpin) structures were found for this sequence!


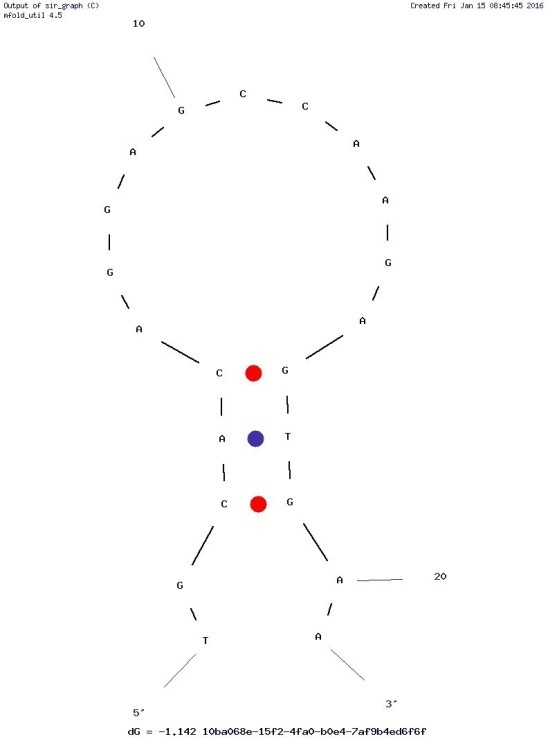


**YWHAZ (NM_003406.3)**

NCBI Reference Sequence for *Homo sapiens* tyrosine 3-monooxygenase/tryptophan 5-monooxygenase activation protein, zeta (**YWHAZ**), transcript variant 1, mRNA.

FASTA Sequence alignment with primers highlighted:

....CAAGTTATAAGTGTTTGGCATAGT**ACTTTTGGTACATTGTGGCTTCAA**AAGGGCCAGTGTAAAACTGCTTCCATGTCTAAGCAAAGAAAACTGCCTAC**ATACTGGTTTGTCCTGGCGG**GGAATAAAAGGGATCATTGGTT.....

**YWHAZ (NM003406)**

Forward:

5’-ACTTTTGGTACATTGTGGCTTCAA-3’

ΔG(kcal.mole^-1^) = **-0.1**

Reverse:

5’-CCGCCAGGACAAACCAGTAT-3’

ΔG(kcal.mole^-1^) = **-1.16**


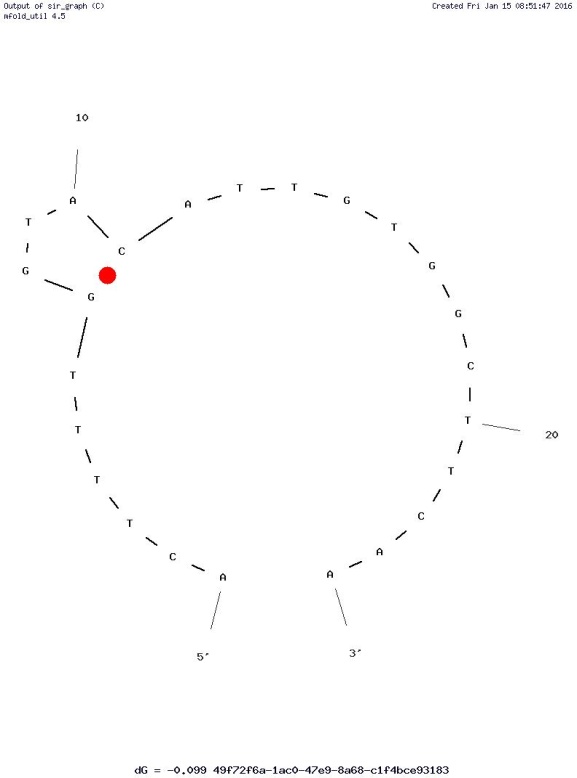


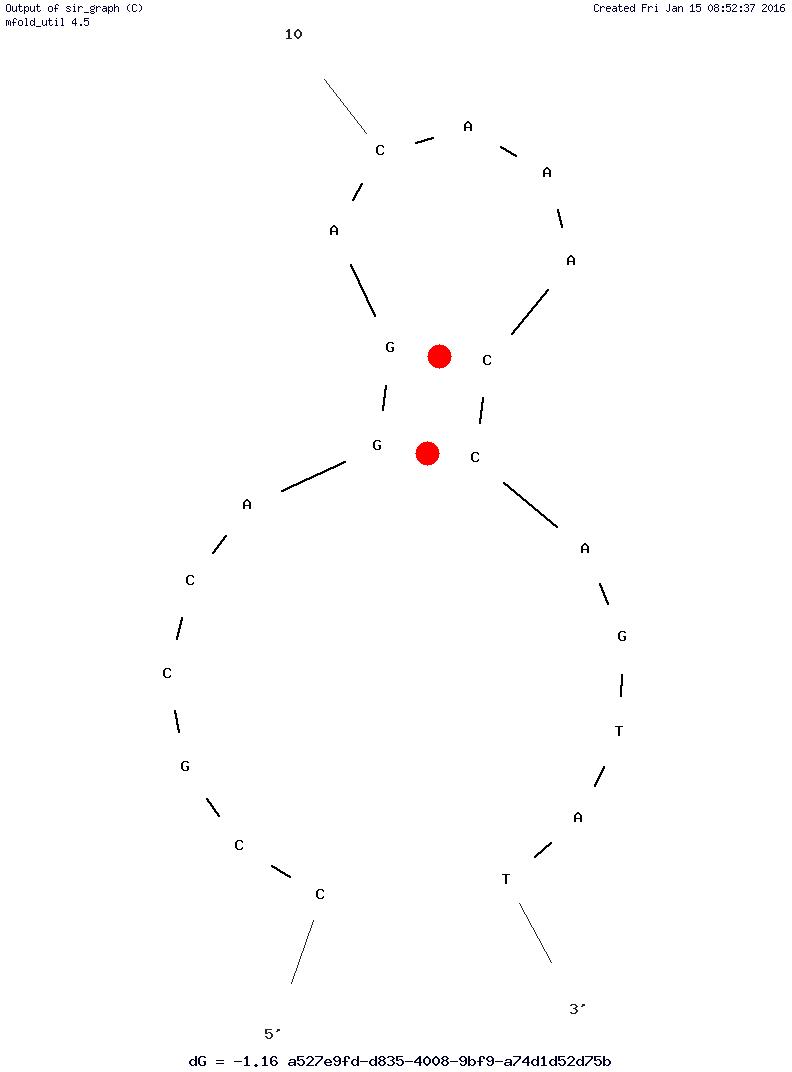

Supplement: Supplementary file 1 — Additional file 1. In silico analysis of primers. [file 12951_2017_299_MOESM1_ESM.docx]
