# Supplementary material for: The presence of residual gold nanoparticles in samples interferes with the RT-qPCR assay used for gene expression profiling
Source: J Nanobiotechnology. 2017 Oct 10;15:72. doi: 10.1186/s12951-017-0299-9 (PMC5633869; doi:10.1186/s12951-017-0299-9)
Supplement: Supplementary file 2 — Additional file 2. Assessment of the qPCR amplification of the reference genes, as influenced by AuNP treatment of BEAS-2B human cell line. [file 12951_2017_299_MOESM2_ESM.docx]

**Additional File 2: Analysis of RNA obtained from the BEAS-2B human cell line after treatment with AuNPs:**

Title: The presence of residual gold nanoparticles in samples interferes with the RT-qPCR assay used for gene expression profiling.

Authors: Natasha M Sanabria and Mary Gulumian

RNA was obtained from 3 biological repeats, where BEAS-2B cells were treated with AuNPs for 24 h. This RNA was reverse transcribed to generate cDNA. Again, a mix of an oligo-dT primer and a random hexamer was used. This cDNA was then amplified using 10 reference gene primer pairs and a SYBR Green PCR super-mix (for basic melt curve profiles). The BestKeeper analysis is shown in Table1. The NormFinder analysis is indicated in Table 2. The REST analysis is shown in Figure 1. The CFX Manager software was used to obtain the PCR efficiency (E), the linearity of the PCR assay (R^2^) as well as the slope obtained for the standard curve, which is summarised below (Table 3 and 4). All 10 primer pairs did amplify and produced PCR products (amplicons), i.e. the amplification was successful. The melt peak for each primer pair is shown below in the respective figures (Figures 2 to 6). Except for the 18S sample, all “non-template controls” (NTC) did not amplify before 35 cycles and confirmed that the experimental conditions were free of contaminating DNA/RNA.

The amplification step included both untreated/control samples, as well as, samples isolated after a 24 h exposure to AuNPs. These samples, thus, represented early gene expression after a short exposure time and had biological significance for ENM-related genotoxicity assays. These preliminary results indicate variation in the quantitative cycle (C_q_) between the control and treated samples. It must be emphasised that a full set of samples must be tested from various induction time points, i.e. the time-study, in biological triplicate, before one can be certain that the reference genes are truly stable for a particular experimental condition.

**Table 1:** Summarised results for BEAS-2B, showing initial descriptive statistics and secondary correlations, as generated by BestKeeper analyses.

|  | **Least Stable** |  |  |  |  |  |  |  |  | **Most stable** |
| --- | --- | --- | --- | --- | --- | --- | --- | --- | --- | --- |
| **SD** | TBP (4.87) | 18S (2.00) | PPI (1.11) | ACTB (1.06) | SDH (0.99) | HSP90 (0.53) | HPRT1 (0.52) | **YWHAZ** (0.42) | **GUSB** (0.25) | **GAPDH** (0.22) |
| **CV** | TBP (22.64) | 18S (17.37) | PPI (7.10) | ACTB (7.06) | SDH (4.99) | HSP90 (2.96) | HPRT1 (2.47) | **YWHAZ** (2.34) | **GAPDH** (1.44) | **GUSB** (1.14) |
| ***r*** | GUSB (-0.120) | GAPDH (0.065) | YWHAZ (0.111) | HPRT1 (0.370) | HSP90 (0.390) | SDH (0.499) | 18S (0.504) | PPI (0.535) | ACTB (0.543) | TBP (0.750) |

**SD**: Standard deviation **(***stability= ranked low to high**)**; **CV**: Coefficient of variance **(****stability = ranked low to high); ***r***: Pearson correlation coefficient (*******stability = ranked high to low)

**Table 2:** The summarised results for **BEAS-2B generated by the NormFinder analyses**.

| **Reference Gene** | **NormFinder Technical repeat 1** | **NormFinder Technical repeat 2** | **NormFinder Technical repeat 3** | **NormFinder Average** | **Manual Ranking using the NormFinder Average of the Reference Gene** | **NormFinder Global summary of the best gene*** |
| --- | --- | --- | --- | --- | --- | --- |
| **18S** | (0.298) | (0.499) | (0.003) | 0.267 | **1 ACTB(Most stable)**  **2 PPI**  **3 HSP90**  **4 GUSB**  **5 SDH**  **6 HPRT1**  **7 GAPDH**  **8 YWHAZ**  **9 18S**  **10 TBP (Least stable)** | **GAPDH (0.005)**  **GAPDH & HSP90 (0.013)** |
| **ACTB** | (0.218) | (0.003) | (0.030) | 0.084 |  |  |
| **GAPDH** | (0.282) | (0.297) | (0.110) | 0.197 |  |  |
| **GUSB** | (0.330) | (0.003) | (0.026) | 0.120 |  |  |
| **HPRT1** | (0.411) | (0.042) | (0.084) | 0.179 |  |  |
| **HSP90** | (0.012) | (0.251) | (0.083) | 0.116 |  |  |
| **PPI** | (0.215) | (0.055) | (0.065) | 0.112 |  |  |
| **SDH** | (0.016) | (0.345) | (0.038) | 0.133 |  |  |
| **TBP** | (0.012) | (1.530) | (0.019) | 0.520 |  |  |
| **YWHAZ** | (0.607) | (0.112) | (0.012) | 0.244 |  |  |

****The best gene has the lowest stability value.***


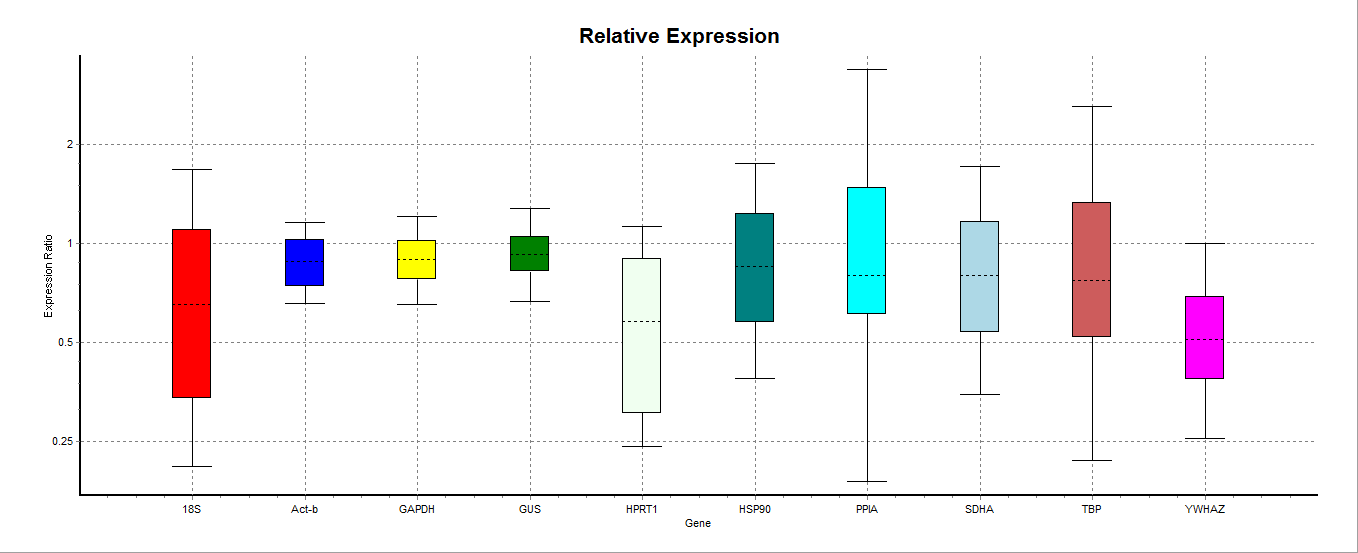


**Figure 1:** Summarised results for REST analysis.

Note: Only 2 biological repeats were used for this analysis

**Table 3:** Summarised results for BEAS-2B during AuNP-interference assessment, using CFX Manager^TM^ Software.

|  | 18S | ACTB | GAPDH | **GUSB** | HPRT1 | HSP90 | PPI | SDH | **TBP** | **YWHAZ** |
| --- | --- | --- | --- | --- | --- | --- | --- | --- | --- | --- |
| **E** (90 to 110%)***** | ***79.8-104.2*** | ***98,8-167.7*** | **85.9-98.5** | **95.5-102.6** | **83.2-100.2** | **83.2-100.4** | **82.7-98.6** | **94.7-136.8** | **91.8-104.3** | **89-96.2** |
| **R^2^** (>0.980)****** | ***0.945-0.994*** | ***0.986- 1.000*** | **0.997-0.999** | **0.994-0.999** | **0.998-1.000** | **0.999-1.000** | **0.998-0.999** | **0.998-1.000** | **0.997-0.999** | **0.998-1.000** |
| Slope (-3.1 to -3.6)***** | **-3.162 to -3.923** | **-2.338 to -3.352** | **-3.358 to -3.715** | **-3.262 to -3.434** | **-3.318 to -3.804** | **-3.313 to -3.803** | **-3.356 to -3.820** | **-2.671 to -3.456** | **-3.223 to -3.536** | **-3.401 to -3.617** |
| **NTC C_q_** | *33.92* | **N/A** | **N/A** | **N/A** | **N/A** | **N/A** | **N/A** | **N/A** | *37.33* | **N/A** |

**Note:** **Green/bold** = acceptable result; **Red/italics** = unacceptable result; **Black** = base-line result, e.g. for untreated/0%AuNP samples.

*****For an efficiency of 100%, the slope is -3.32. A good reaction should have an efficiency between 90% and 110%, which corresponds to a slope between -3.58 and -3.10 ******R^2^ <0.980 unacceptable; R^2^ ≥ 0.980 acceptable; R^2^ >0.990 expected; R^2^ >0.995 exceptional

**Table 4:** Summarised **C_q_** results for AuNP-interference assessment of BEAS-2B treated with AuNPs, by using CFX Manager^TM^ Software.

|  | 18S C_q_ | ACTB C_q_ | **GAPDH C_q_** | **GUSB C_q_** | **HPRT1 C_q_** | **HSP90 C_q_** | PPI C_q_ | SDH C_q_ | TBP C_q_ | YWHAZ C_q_ |
| --- | --- | --- | --- | --- | --- | --- | --- | --- | --- | --- |
| **Untreated Control** | 9.21 to  14.87 | 13.80 to  16.44 | 15.23 to  15.93 | 21.78 to  22.61 | 19.46 to  21.25 | 17.19 to  18.58 | 13.91 to  16.91 | 18.62 to  21.42 | 20.55  22.62  (8.17) | 17.35 to  18.12 |
| **24 h AuNP Treated** | **9.32 to**  **14.16** | **14.18 to**  16.72 | **15.27 to**  **15.85** | **22.15 to** **22.63** | 21.08 to  21.51 | 17.09 **to 18.55** | **14.14 to**  16.96 | **18.62 to**  **21.03** | **21.24**  22.74  (33.84) | **17.88 to** *19.37* |

**Note:** **Green/bold** = acceptable result; **Red/italics** = unacceptable result; **Black** = base-line result, e.g. for untreated/0%AuNP samples. ***A C_q_ change of 0.2 is acceptable, but >0.5 is unacceptable**.


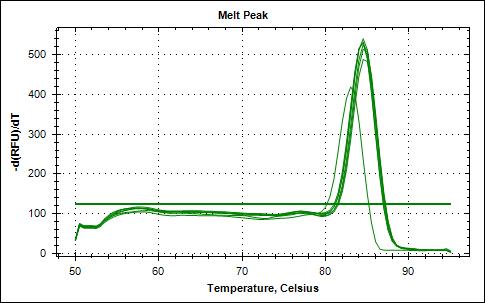

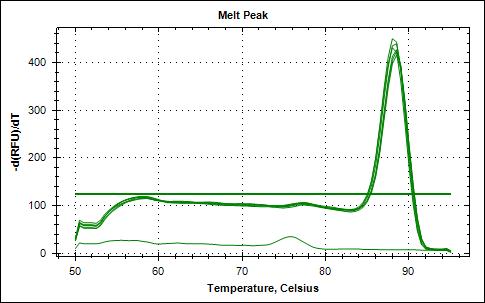


**Figure 2: Dissociation assay profile (melt peak) of (A) 18S and (B) ACTB.**


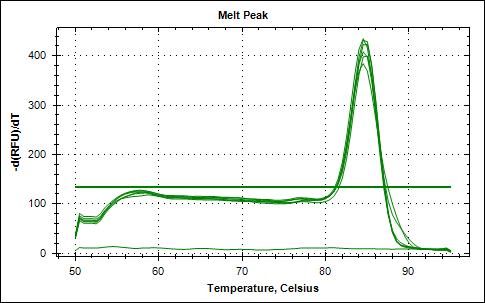

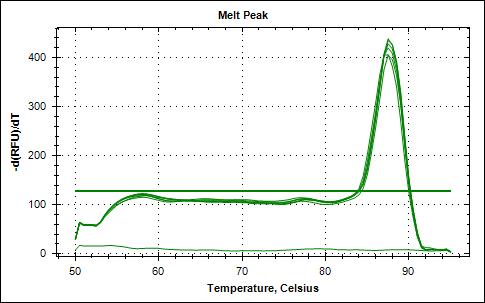


**Figure 3: Dissociation assay profile (melt peak) of (A) GAPDH and (B) GUSB.**

**
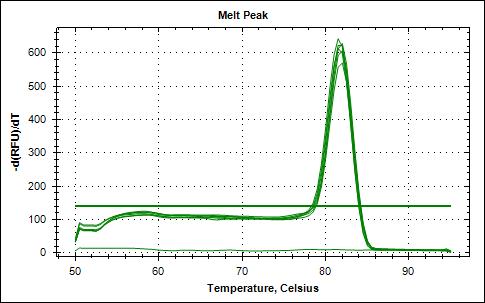
**
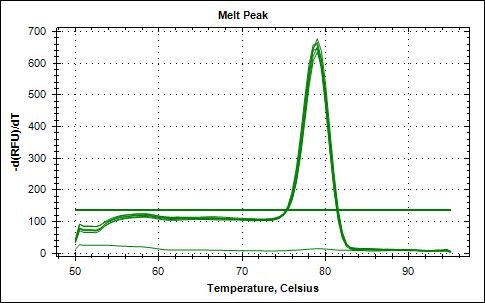


**Figure 4: Dissociation assay profile (melt peak) of (A) HPRT and (B) HSP90.**


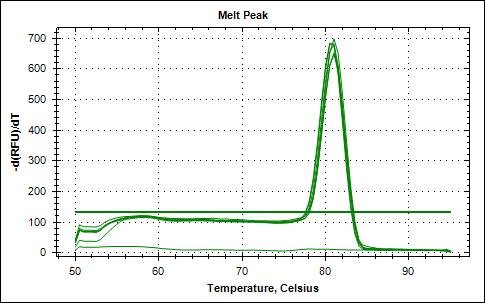

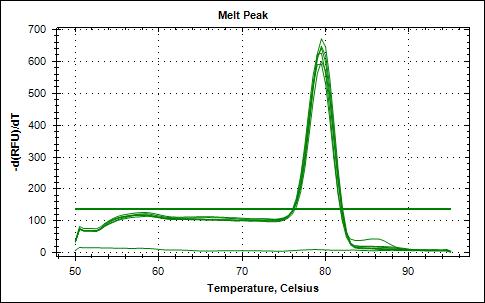


**Figure 5: Dissociation assay profile (melt peak) of (A) PPI and (B) SDH.**


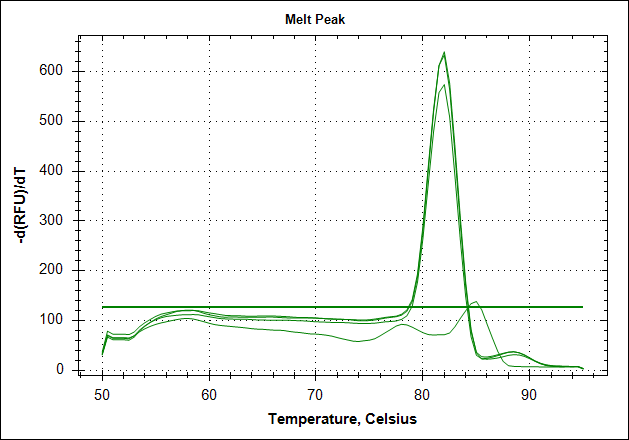

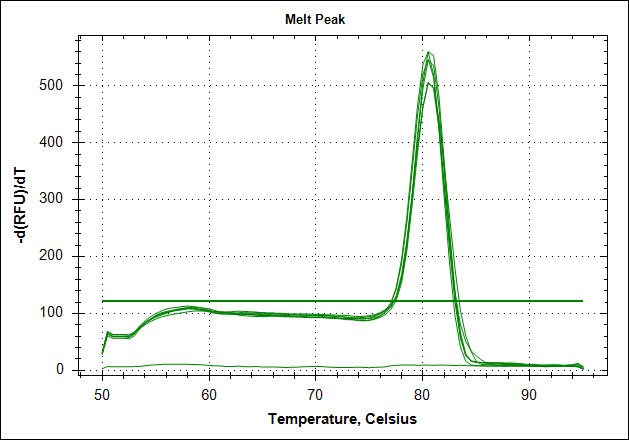


**Figure 6: Dissociation assay profile (melt peak) of (A) TBP and (B) YWHAZ.**
