# Supplementary material for: The presence of residual gold nanoparticles in samples interferes with the RT-qPCR assay used for gene expression profiling
Source: J Nanobiotechnology. 2017 Oct 10;15:72. doi: 10.1186/s12951-017-0299-9 (PMC5633869; doi:10.1186/s12951-017-0299-9)
Supplement: Supplementary file 3 — Additional file 3. Dissociation dynamics melt peaks of qPCR amplification of the reference genes, from the universal RNA standard. [file 12951_2017_299_MOESM3_ESM.docx]

**Additional File 3: Dissociation dynamics (melt peaks) of qPCR amplification of the reference genes, from the universal RNA standard.**

Title: The presence of residual gold nanoparticles in samples interferes with the RT-qPCR assay used for gene expression profiling.

Authors: Natasha M Sanabria and Mary Gulumian

**Part 1:**

The AuNPs were added to the universal RNA standard at the reverse transcription step (part 1 of assessment). This treatment has biological significance because it mimics any possible interference of residual ENMs present in biological samples, which would co-precipitate with isolated RNA intended for analyses during exposure assessment studies. It should be noted that the universal RNA standard does not include BEAS-2B, which was the template used for the preliminary study (see Supplementary data 2). The reference gene expression was screened to determine changes in the dissociation assay (melt peaks) of the different products formed, using the CFX Manager software (see Table 1). Initial observations of only the amplification plots obtained from the CFX Manager software indicated a change in the PCR profiles for some of the reference genes, i.e. before and after deliberate addition of AuNPs (see Figures 1-5). The resulting PCR amplicons from all 10 reference genes were separated on a 1% agarose gel prepared with TBE buffer and stained with ethidium bromide (see Figures 6-15).


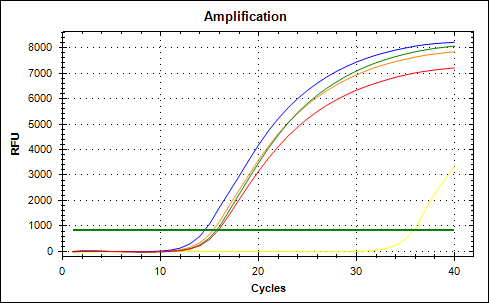

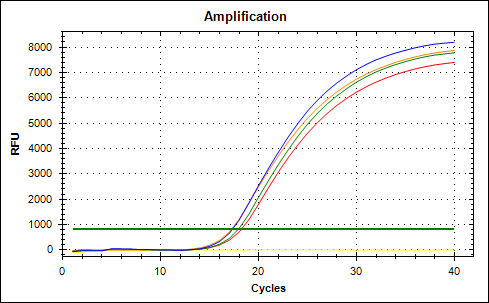


**Figure 1: Amplification plots (A) 18S (B) ACTB.** Untreated/control sample indicated in blue (0%AuNP), treated samples indicated in green (25%AuNP), orange (50%AuNP) and red (75%AuNP). The NTC is indicated in yellow.


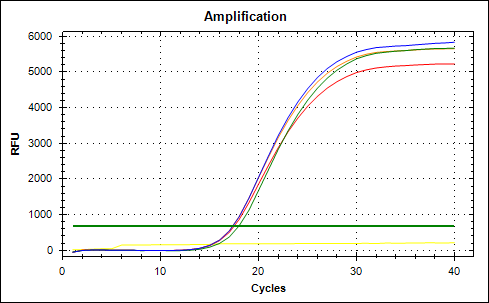

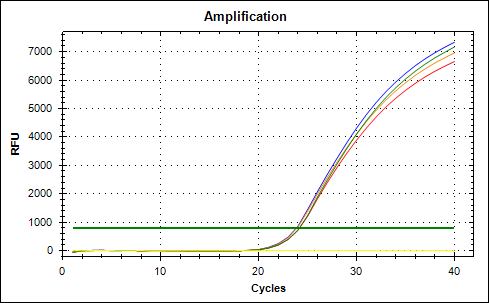


**Figure 2: Amplification plots (A) GAPDH (B) GUSB.** Untreated/control sample indicated in blue (0%AuNP), treated samples indicated in green (25%AuNP), orange (50%AuNP) and red (75%AuNP). The NTC is indicated in yellow.


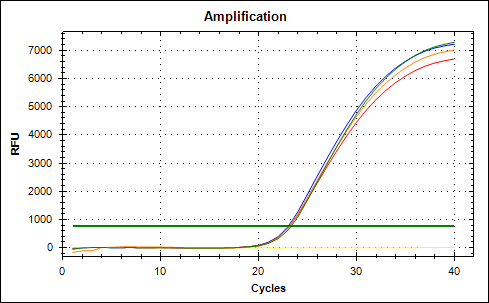

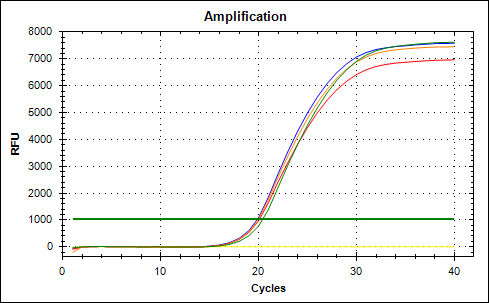


**Figure 3: Amplification plots (A) HPRT1 (B) HSP90.** Untreated/control sample indicated in blue (0%AuNP), treated samples indicated in green (25%AuNP), orange (50%AuNP) and red (75%AuNP). The NTC is indicated in yellow.


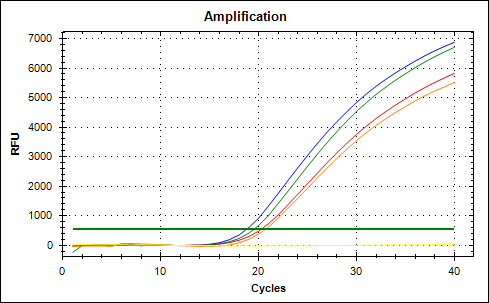

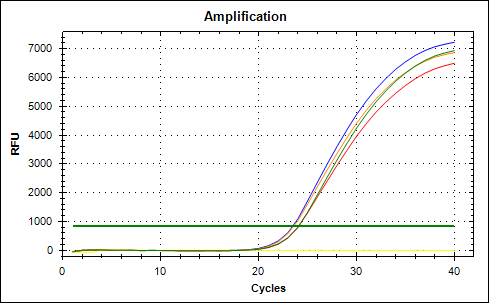


**Figure 4: Amplification plots (A) PPI (B) SDH.** Untreated/control sample indicated in blue (0%AuNP), treated samples indicated in green (25%AuNP), orange (50%AuNP) and red (75%AuNP). The NTC is indicated in yellow.


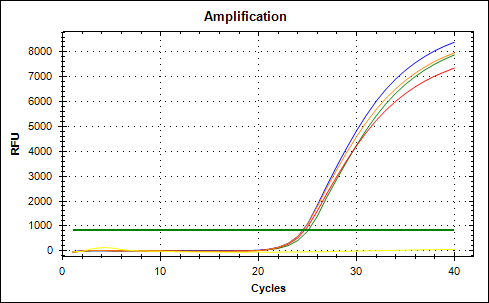

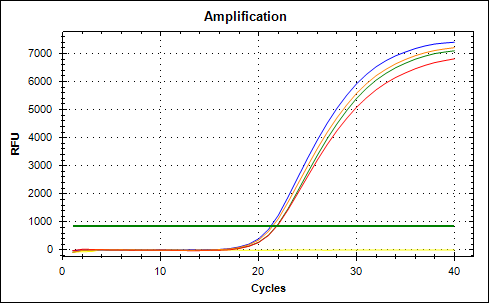


**Figure 5: Amplification plots (A) TBP (B) YWHAZ.** Untreated/control sample indicated in blue (0%AuNP), treated samples indicated in green (25%AuNP), orange (50%AuNP) and red (75%AuNP). The NTC is indicated in yellow.

**Table 1:** Summarised dissociation assay (melt peak) results for the universal RNA standard during AuNP-interference assessment, using CFX Manager software.

| **Melt Peak (**°C) ****** | **18S** | **ACTB** | **GAPDH** | **GUSB** | **HPRT1** | **HSP90** | **PPI** | **SDH** | **TBP** | **YWHAZ** |
| --- | --- | --- | --- | --- | --- | --- | --- | --- | --- | --- |
| **0%**  **AuNP** | 83.00 - 83.20 | 87.00 -87.40 | 83.5 - 83.60 | 86.2 -86.50 | 80.50 -80.60 | 77.50 - 77.60 | 79.50 - 79.80 | 78.00 - 78.20 | 80.50- 80.80 | 79.20-79.50 |
| **25%**  **AuNP** | 83.00 | 87.00-87.40 | 83.50-83.60 | 86.20-86.50 | 80.50 -80.60 | 77.50 - 77.60 | 79.50-79.60 | 78.00 - 78.20 | 80.50- 80.60 | 79.00-79.40 |
| **50%**  **AuNP** | 83.00-83.20 | 87.00-87.40 | 83.50-83.60 | 86.20-86.50 & *88.0* | 80.50 -80.60 | 77.40 - 77.60 | 79.40-79.60 | 78.00 - 78.20 | 80.50- 80.60 | 79.00-79.40 |
| **75%**  **AuNP** | 83.00- 83.20 | 87.00-87.40 | 83.50-83.80 | 86.20-86.50 | 80.40-80.60 | 77.40 - 77.60 | 79.40-79.60 | 78.00 - 78.20 | 80.50- 80.60 | 79.00-79.40 |
| **Number of peaks** | 1 peak @ 83 |  |  | 2 peaks @86, 88 |  |  |  |  |  |  |

**Note:** A PCR product represented by 1 melt peak is acceptable, where multiple products are not acceptable in this qPCR assay.

**(A) (B)**


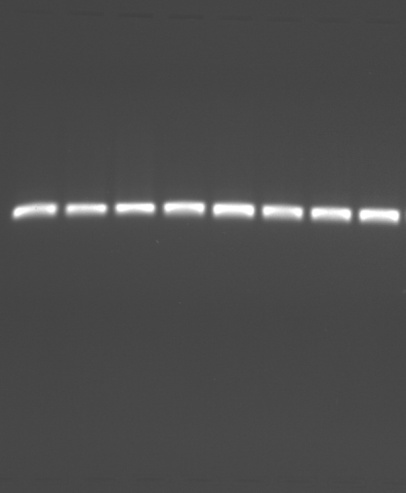


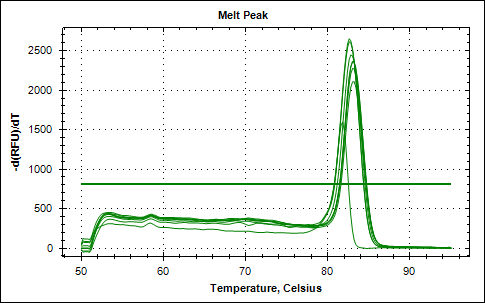


**Figure 6: Summarised results for 18S. (A) Dissociation assay profile (melt peak) of 18S, with (B) PCR amplicons separated by electrophoresis.** Lane (1) Undiluted qPCR standard (2) 2xDilution qPCR standard (3) 10xDilution qPCR standard (4) 20xDilution qPCR standard (5) Untreated/control sample (0%AuNP) (6) 25%AuNP sample (7) 50%AuNP sample (8) 75%AuNP sample.

**(A) (B)**


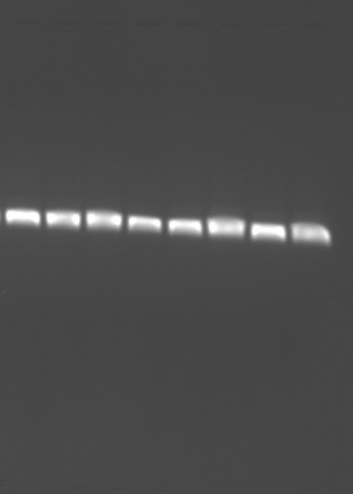


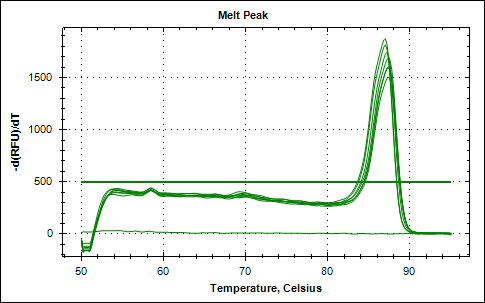


**Figure 7: Summarised results for ACTB. (A) Dissociation assay profile (melt peak) of ACTB.**

**(B) PCR amplicons separated by electrophoresis.** Lane (1) Undiluted qPCR standard (2) 2xDilution qPCR standard (3) 10xDilution qPCR standard (4) 20xDilution qPCR standard (5) Untreated/control sample (0%AuNP) (6) 25%AuNP sample (7) 50%AuNP sample (8) 75%AuNP sample.

**(A) (B)**

**
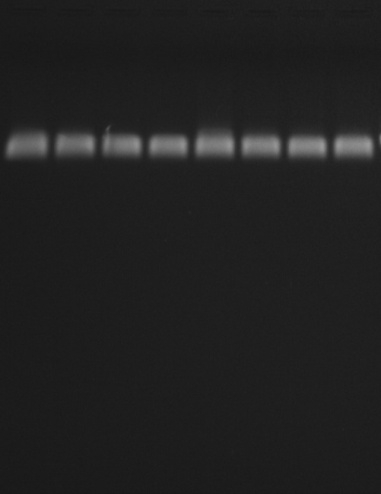
**


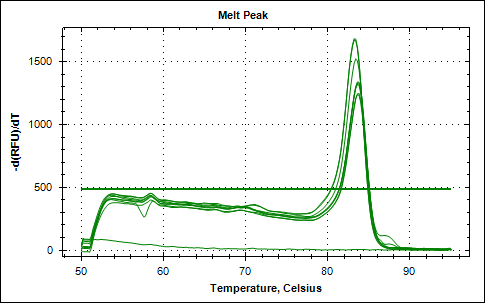


**Figure 8: Summarised results for GAPDH. (A) Dissociation assay profile (melt peak) of GAPDH. (B) PCR amplicons separated by electrophoresis.** Lane (1) Undiluted qPCR standard (2) 2xDilution qPCR standard (3) 10xDilution qPCR standard (4) 20xDilution qPCR standard (5) Untreated/control sample (0%AuNP) (6) 25%AuNP sample (7) 50%AuNP sample (8) 75%AuNP sample.

**(A) (B)**


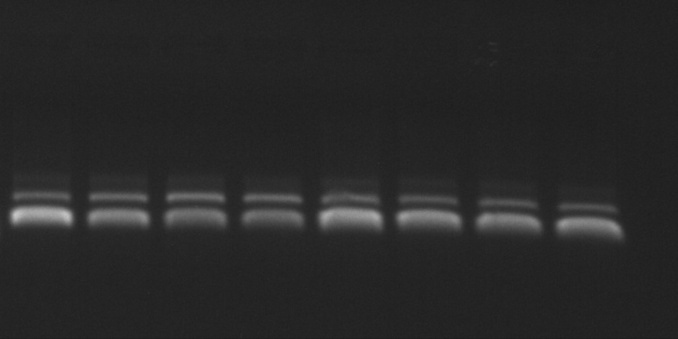


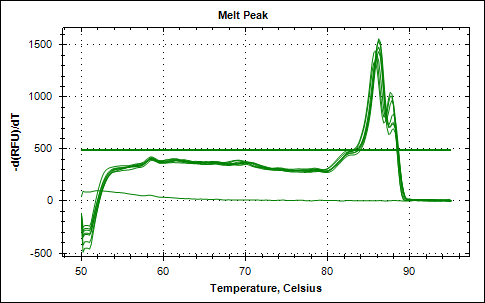


**Figure 9: Summarised results for GUSB. (A) Dissociation assay profile (melt peak) of GUSB. (B) PCR amplicons separated by electrophoresis.** Lane (1) Undiluted qPCR standard (2) 2xDilution qPCR standard (3) 10xDilution qPCR standard (4) 20xDilution qPCR standard (5) Untreated/control sample (0%AuNP) (6) 25%AuNP sample (7) 50%AuNP sample (8) 75%AuNP sample.

**(A) (B)**


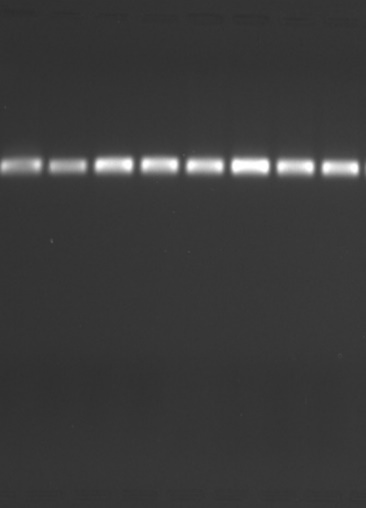


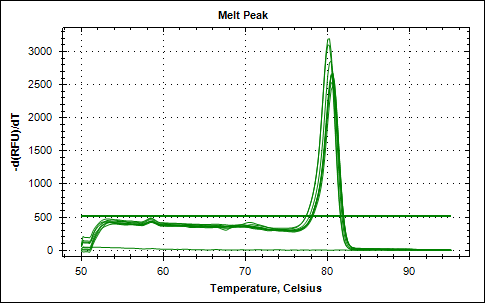


**Figure 10: Summarised results for HPRT1. (A) Dissociation assay profile (melt peak) of HPRT1. (B) PCR amplicons separated by electrophoresis.** Lane (1) Undiluted qPCR standard (2) 2xDilution qPCR standard (3) 10xDilution qPCR standard (4) 20xDilution qPCR standard (5) Untreated/control sample (0%AuNP) (6) 25%AuNP sample (7) 50%AuNP sample (8) 75%AuNP sample.

**(A) (B)**


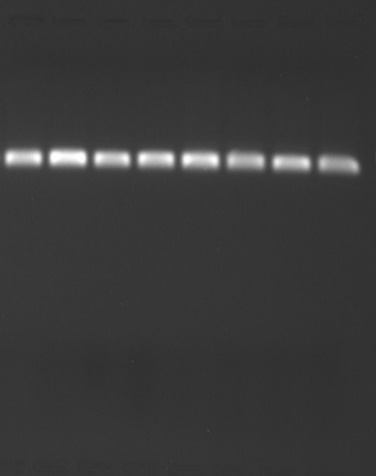


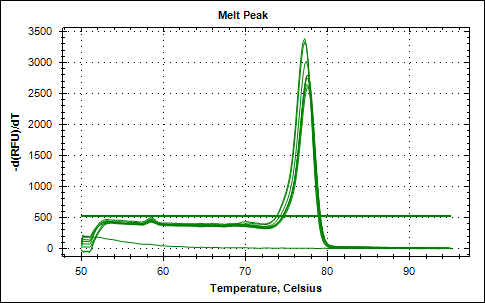


**Figure 11: Summarised results for HSP90. (A) Dissociation assay profile (melt peak) of HSP90. (B) PCR amplicons separated by electrophoresis.** Lane (1) Undiluted qPCR standard (2) 2xDilution qPCR standard (3) 10xDilution qPCR standard (4) 20xDilution qPCR standard (5) Untreated/control sample (0%AuNP) (6) 25%AuNP sample (7) 50%AuNP sample (8) 75%AuNP sample.

**(A) (B)**


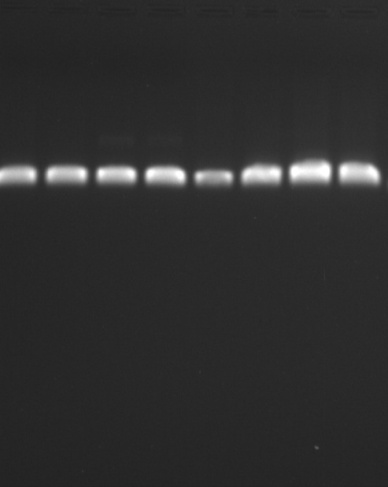


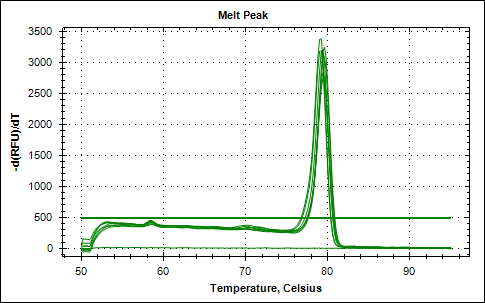


**Figure 12: Summarised results for PPI. (A) Dissociation assay profile (melt peak) of PPI. (B) PCR amplicons separated by electrophoresis.** Lane (1) Undiluted qPCR standard (2) 2xDilution qPCR standard (3) 10xDilution qPCR standard (4) 20xDilution qPCR standard (5) Untreated/control sample (0%AuNP) (6) 25%AuNP sample (7) 50%AuNP sample (8) 75%AuNP sample.

**(A) (B)**

**
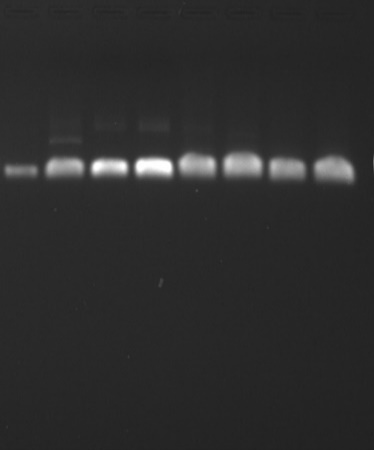
**


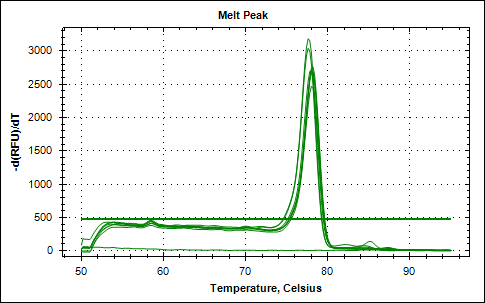


**Figure 13: Summarised results for SDH. (A) Dissociation assay profile (melt peak) of SDH. (B) PCR amplicons separated by electrophoresis.** Lane (1) Undiluted qPCR standard (2) 2xDilution qPCR standard (3) 10xDilution qPCR standard (4) 20xDilution qPCR standard (5) Untreated/control sample (0%AuNP) (6) 25%AuNP sample (7) 50%AuNP sample (8) 75%AuNP sample.

**(A) (B)**


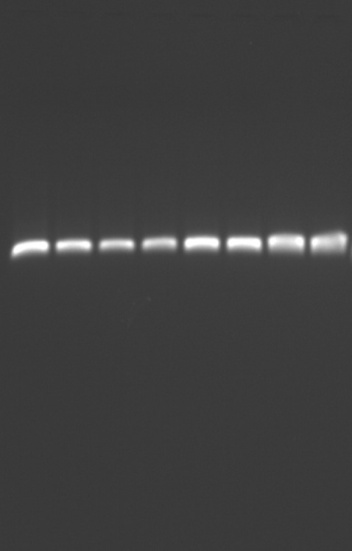


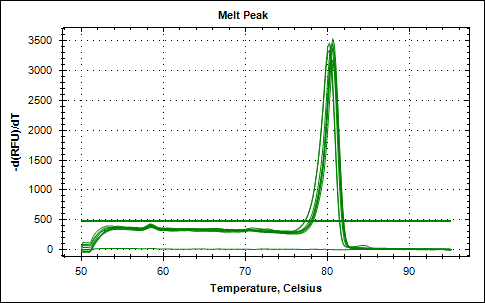


**Figure 14: Summarised results for TBP. (A) Dissociation assay profile (melt peak) of TBP. (B) PCR amplicons separated by electrophoresis.** Lane (1) Undiluted qPCR standard (2) 2xDilution qPCR standard (3) 10xDilution qPCR standard (4) 20xDilution qPCR standard (5) Untreated/control sample (0%AuNP) (6) 25%AuNP sample (7) 50%AuNP sample (8) 75%AuNP sample.

**(A) (B)**

**
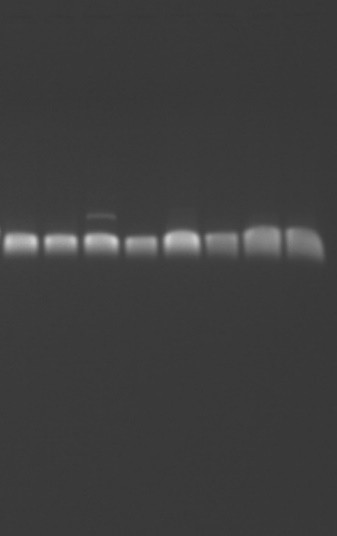
**


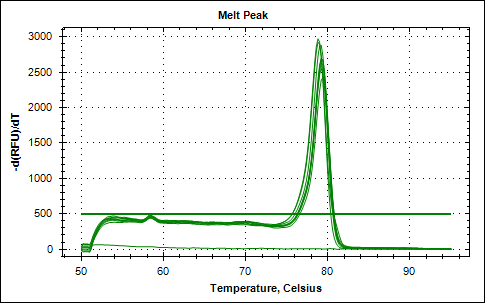


**Figure 15: Summarised results for YWHAZ. (A) Dissociation assay profile (melt peak) of YWHAZ. (B) PCR amplicons separated by electrophoresis.** Lane (1) Undiluted qPCR standard (2) 2xDilution qPCR standard (3) 10xDilution qPCR standard (4) 20xDilution qPCR standard (5) Untreated/control sample (0%AuNP) (6) 25%AuNP sample.

**Part 2:**

­­­­­­­In the second series of experiments (**part 2**), the universal RNA standard was reverse transcribed to generate cDNA. The PCR step was spiked with AuNPs and the DNA was amplified. This represented a technical control sample within the experiment, but did not provide any biological significance, i.e. any residual ENMs would be present after the isolation procedure and before reverse transcription. It should be noted that only a 25% spike was added to the PCR step due to restrictions on the final volume of the reaction. As mentioned in the previous sections, the amplification plots identified changes in the PCR profiles for the reference genes, i.e. before and after deliberate addition of 25% AuNPs (see Figures 16-20). The reference gene expression was screened to determine changes in the dissociation assay (melt peaks) of the different products formed, using the **CFX Manager** software (see Table 2). Amplification plots were not that informative in part 2, since only once sample type was measured (25%), i.e. the difference could not be associated with a concentration dependent response. However, the resulting PCR amplicons from all 10 reference genes were separated on a 1% agarose gel prepared with TBE buffer and stained with ethidium bromide (see Figures 21-30).


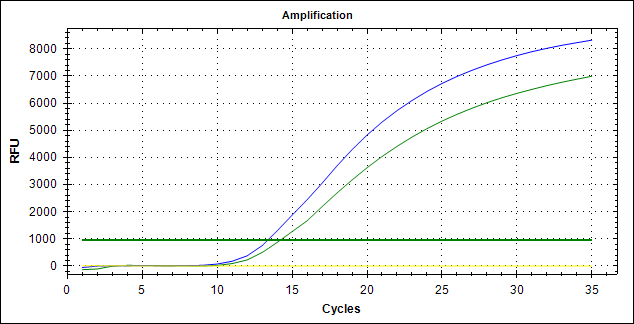

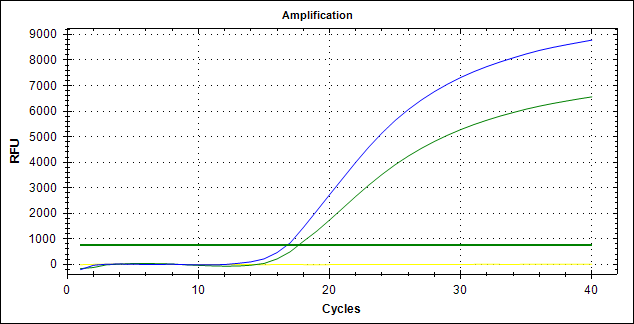


**Figure 16: Amplification plots (A) 18S (B) ACTB.** Untreated/control sample indicated in blue (0%AuNP), treated samples indicated in green (25%AuNP) and the NTC is indicated in yellow.


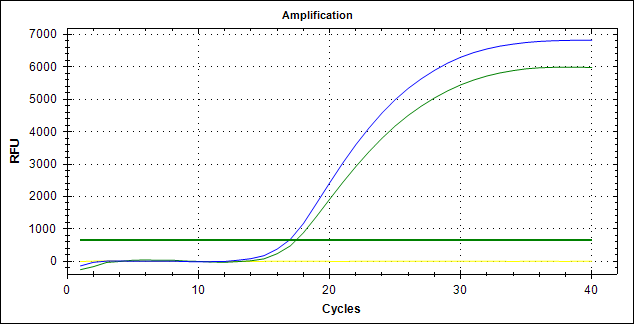

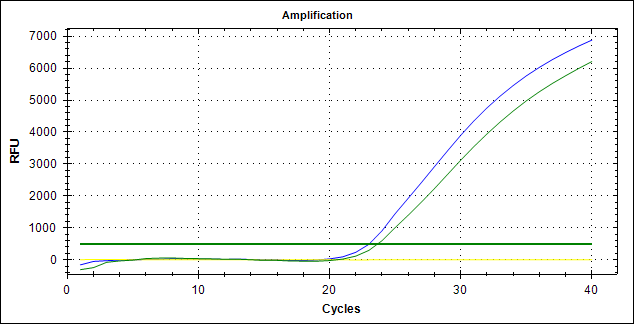


**Figure 17: Amplification plots (A) GAPDH (B) GUSB.** Untreated/control sample indicated in blue (0%AuNP), treated samples indicated in green (25%AuNP) and the NTC is indicated in yellow.


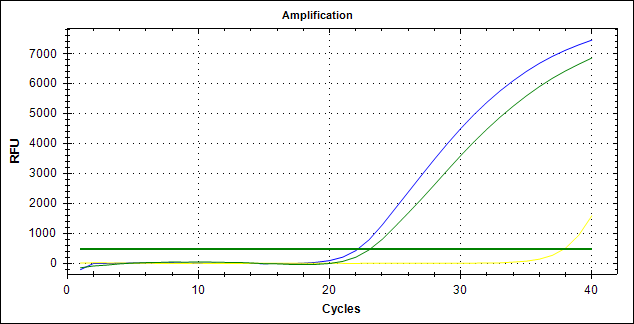

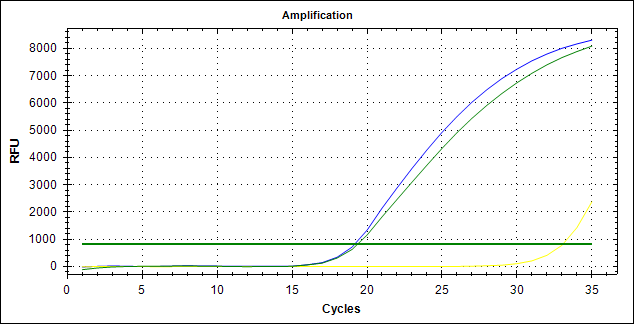


**Figure 18: Amplification plots (A) HPRT1 (B) HSP90.** Untreated/control sample indicated in blue (0%AuNP), treated samples indicated in green (25%AuNP) and the NTC is indicated in yellow.


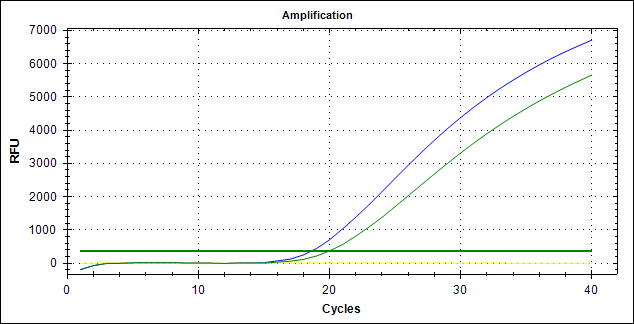

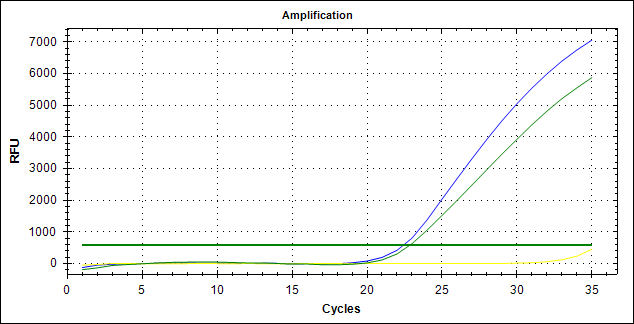


**Figure 19: Amplification plots (A) PPI (B) SDH.** Untreated/control sample indicated in blue (0%AuNP), treated samples indicated in green (25%AuNP) and the NTC is indicated in yellow.


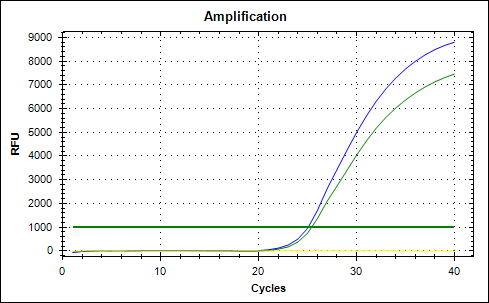

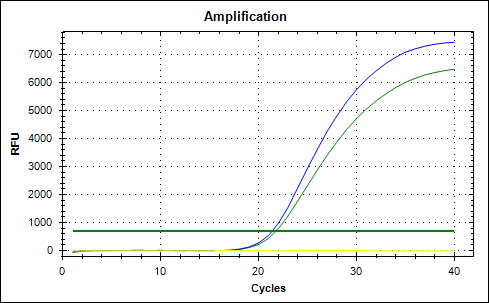


**Figure 20: Amplification plots (A) TBP (B) YWHAZ.** Untreated/control sample indicated in blue (0%AuNP), treated samples indicated in green (25%AuNP) and the NTC is indicated in yellow.

**Table 2:** Summarised dissociation assay (melt peak) results for the universal RNA standard during **AuNP-interference assessment,** using **CFX Manager** software.

| **Melt Peak (**°C) ****** | **18S** | **ACTB** | **GAPDH** | **GUSB** | **HPRT1** | **HSP90** | **PPI** | **SDH** | **TBP** | **YWHAZ** |
| --- | --- | --- | --- | --- | --- | --- | --- | --- | --- | --- |
| **0%**  **AuNP** | 83.00 | 87.20 | 83.60 | 86 & 87.80 | 80.40 | 77.40 | 79.60 | 78.20 | 80.60 | 79.40 |
| **25%**  **AuNP** | 83.00 | 87.20 | 83.60 | 86 &88 | 80.40 | 77.40 | 79.40 | 78.00 | 80.60 | 79.20 |
| **Number of peaks** |  |  |  | 2 peaks @86, 88 |  |  |  |  |  |  |

**Note:** A PCR product represented by **1 melt peak is acceptable, where multiple products are not acceptable** in this qPCR assay.

**(A) (B)**


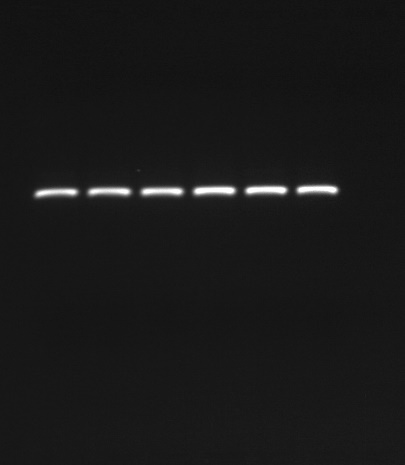


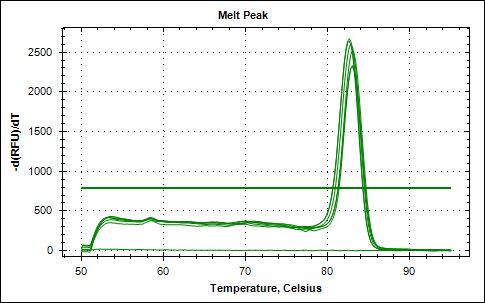


**Figure 21: Summarised results for18S. (A) Dissociation assay profile (melt peak) of 18S, with (B) PCR amplicons separated by electrophoresis.** Lane (1) Undiluted qPCR standard (2) 2xDilution qPCR standard (3) 10xDilution qPCR standard (4) 20xDilution qPCR standard (5) Untreated/control sample (0%AuNP) (6) 25%AuNP sample.

**(A) (B)**

**
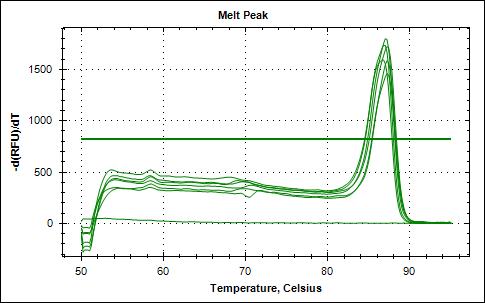
**
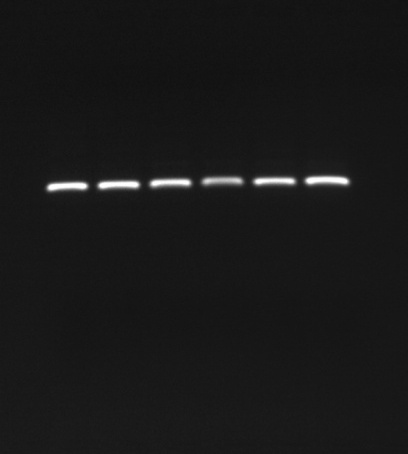


**Figure 22: Summarised results for ACTB. (A) Dissociation assay profile (melt peak) of ACTB, with (B) PCR amplicons separated by electrophoresis.** Lane (1) Undiluted qPCR standard (2) 2xDilution qPCR standard (3) 10xDilution qPCR standard (4) 20xDilution qPCR standard (5) Untreated/control sample (0%AuNP) (6) 25%AuNP sample.

**(A) (B)**


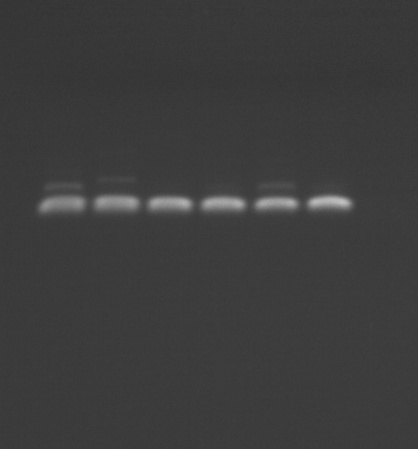


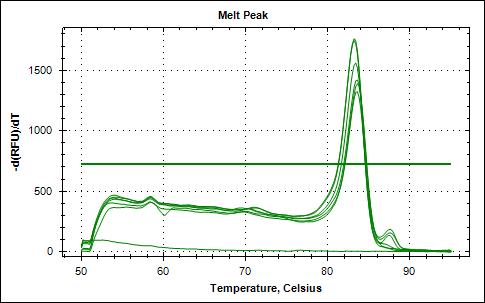


**Figure 23: Summarised results for GAPDH. (A) Dissociation assay profile (melt peak) of GAPDH, with (B) PCR amplicons separated by electrophoresis.** Lane (1) Undiluted qPCR standard (2) 2xDilution qPCR standard (3) 10xDilution qPCR standard (4) 20xDilution qPCR standard (5) Untreated/control sample (0%AuNP) (6) 25%AuNP sample.

**(A) (B)**


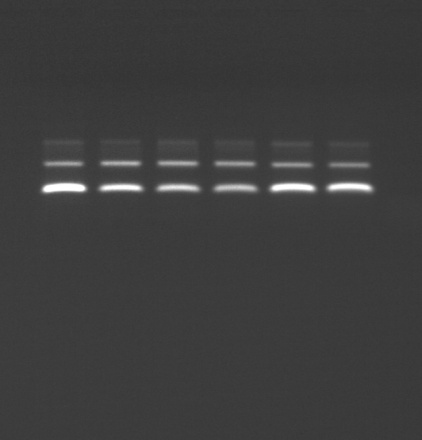


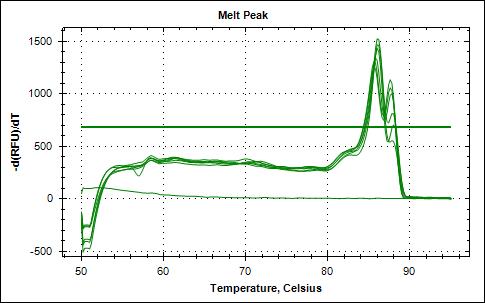


**Figure 24: Summarised results for GUSB. (A) Dissociation assay profile (melt peak) of GUSB, with (B) PCR amplicons separated by electrophoresis.** Lane (1) Undiluted qPCR standard (2) 2xDilution qPCR standard (3) 10xDilution qPCR standard (4) 20xDilution qPCR standard (5) Untreated/control sample (0%AuNP) (6) 25%AuNP sample.

**(A) (B)**


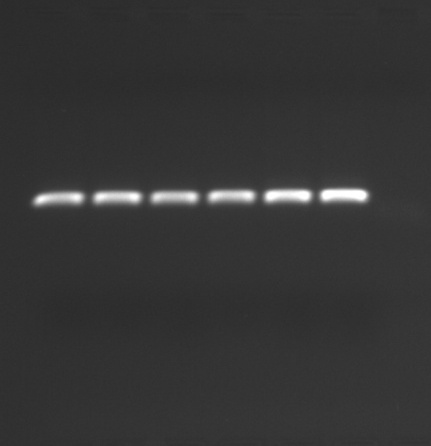


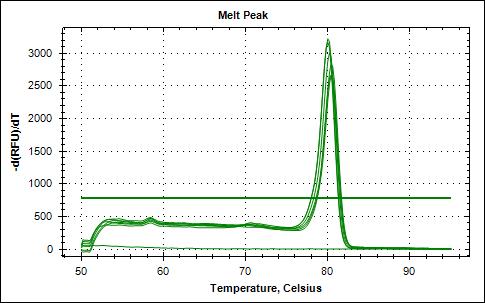


**Figure 25: Summarised results for HPRT1. (A) Dissociation assay profile (melt peak) of HPRT1, with (B) PCR amplicons separated by electrophoresis.** Lane (1) Undiluted qPCR standard (2) 2xDilution qPCR standard (3) 10xDilution qPCR standard (4) 20xDilution qPCR standard (5) Untreated/control sample (0%AuNP) (6) 25%AuNP sample.

**(A) (B)**


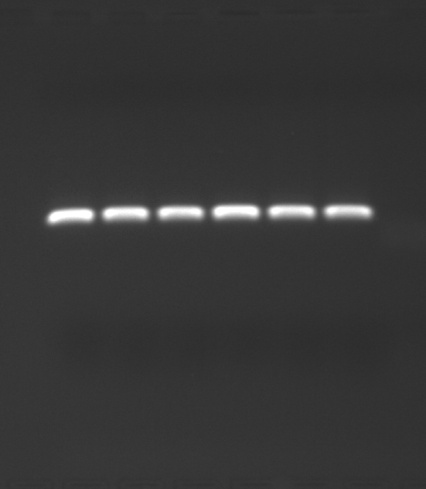


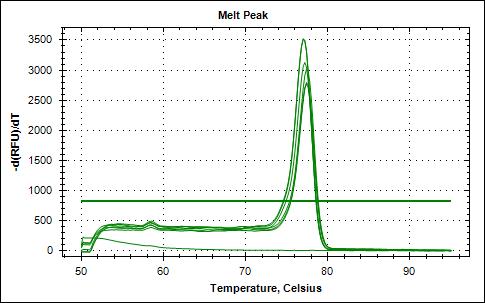


**Figure 26: Summarised results for HSP90. (A) Dissociation assay profile (melt peak) of HSP90, with (B) PCR amplicons separated by electrophoresis.** Lane (1) Undiluted qPCR standard (2) 2xDilution qPCR standard (3) 10xDilution qPCR standard (4) 20xDilution qPCR standard (5) Untreated/control sample (0%AuNP) (6) 25%AuNP sample.

**(A) (B)**


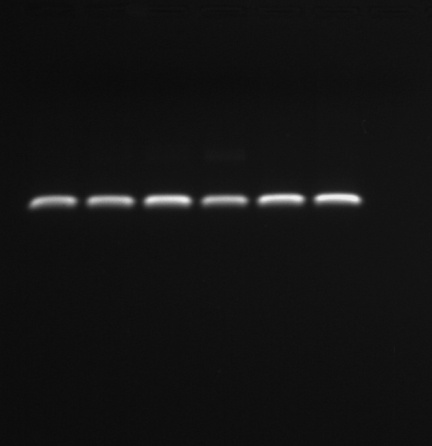


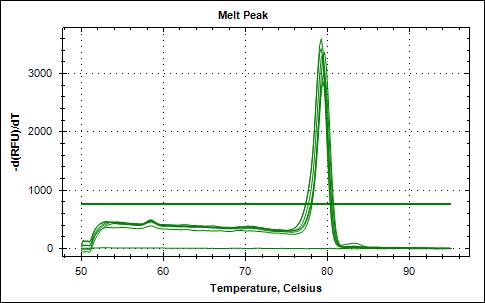


**Figure 27: Summarised results for PPI. (A) Dissociation assay profile (melt peak) of PPI, with (B) PCR amplicons separated by electrophoresis.** Lane (1) Undiluted qPCR standard (2) 2xDilution qPCR standard (3) 10xDilution qPCR standard (4) 20xDilution qPCR standard (5) Untreated/control sample (0%AuNP) (6) 25%AuNP sample

**(A) (B)**


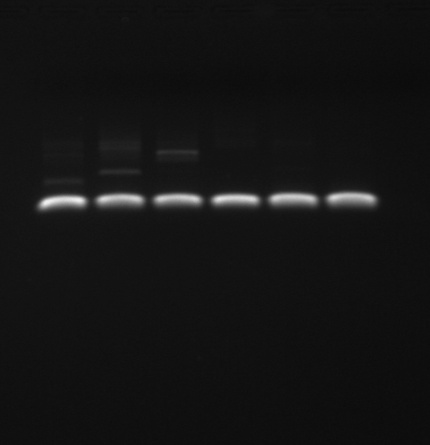


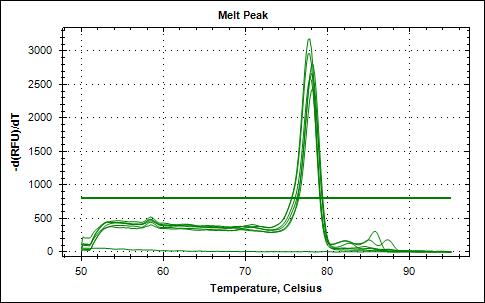


**Figure 28: Summarised results for SDH. (A) Dissociation assay profile (melt peak) of SDH, with (B) PCR amplicons separated by electrophoresis.** Lane (1) Undiluted qPCR standard (2) 2xDilution qPCR standard (3) 10xDilution qPCR standard (4) 20xDilution qPCR standard (5) Untreated/control sample (0%AuNP) (6) 25%AuNP sample.

**(A) (B)**


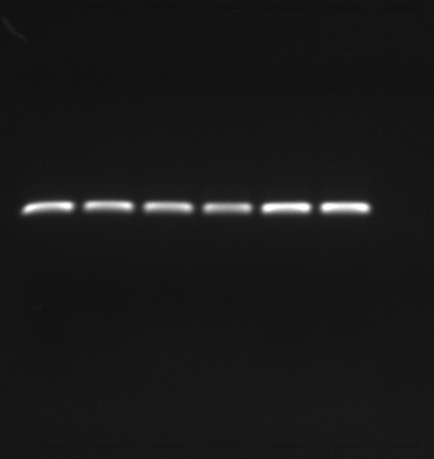


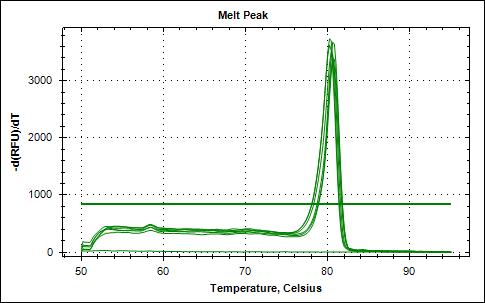


**Figure 29: Summarised results for TBP. (A) Dissociation assay profile (melt peak) of TBP, with (B) PCR amplicons separated by electrophoresis.** Lane (1) Undiluted qPCR standard (2) 2xDilution qPCR standard (3) 10xDilution qPCR standard (4) 20xDilution qPCR standard (5) Untreated/control sample (0%AuNP) (6) 25%AuNP sample

**(A) (B)**


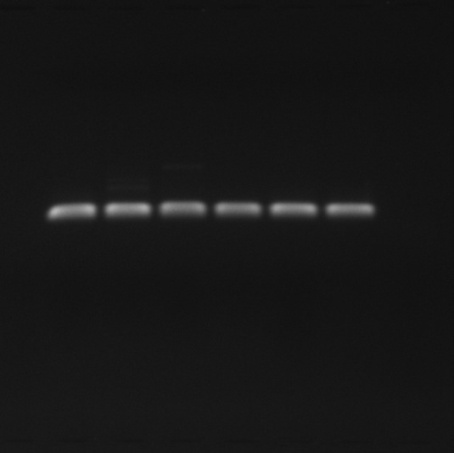


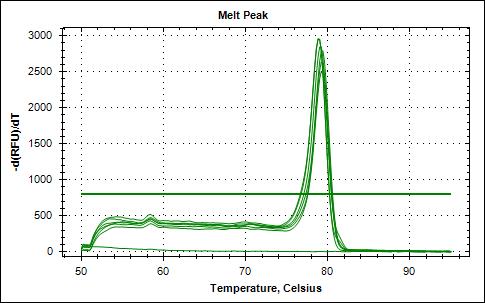


**Figure 30: Summarised results for YWHAZ. (A) Dissociation assay profile (melt peak) of YWHAZ, with (B) PCR amplicons separated by electrophoresis.** Lane (1) Undiluted qPCR standard (2) 2xDilution qPCR standard (3) 10xDilution qPCR standard (4) 20xDilution qPCR standard (5) Untreated/control sample (0%AuNP) (6) 25%AuNP sample.
