# Supplementary material for: The presence of residual gold nanoparticles in samples interferes with the RT-qPCR assay used for gene expression profiling
Source: J Nanobiotechnology. 2017 Oct 10;15:72. doi: 10.1186/s12951-017-0299-9 (PMC5633869; doi:10.1186/s12951-017-0299-9)
Supplement: Supplementary file 4 — Additional file 4. Traditional software and statistical analyses. [file 12951_2017_299_MOESM4_ESM.docx]

**Additional File 4: Traditional software and statistical analyses:**

Title: The presence of residual gold nanoparticles in samples interferes with the RT-qPCR assay used for gene expression profiling.

Authors: Natasha M Sanabria and Mary Gulumian

The **BestKeeper** software determined the best reference genes from a pool of 10 candidates and, thereafter, combined them to create an index (Pfaffl et al., 2004). Target genes may then be compared to this index in order to determine if the targets are differentially expressed under a certain treatment being applied. After the reference gene (HKG) expression levels have been calculated, the expression stability can be estimated based on the statistical variations, i.e. SD and CV values. The reference genes are ordered/ranked from the most stably expressed (which exhibits the lowest variation), to the least stable reference gene (which exhibits the highest variation). For example, a gene with an SD that is higher than 1 indicates a starting template variation by the factor 2 (Pfaffl et al., 2004), which can be considered as inconsistent. All the highly correlated reference genes are combined, where the geometric mean of the Cq values are used to calculate the BestKeeper index. In order to estimate inter-gene relations, Pearson correlation between the reference gene and the index generates the “BestKeeper correlation coefficient” [*r*] (McCulloch et al., 2012). The highest ranked gene is considered to be the most stable. The results obtained in this identified **GUSB, SDH, YWHAZ and HSP90** as suitable reference genes, where stability was inferred by the lowest variation (SD; CV) and the highest inter-gene relationship (*r*).

The **NormFinder** software generated a stability value for each gene that represented a direct measure for the expression variation estimation. Therefore, it was possible to evaluate systematic error introduced when using that specific candidate reference gene for normalisation. The suitable reference genes for normalisation were identified as a combination of **GUSB with HSP90**, where the top ranked gene had the lowest stability value. The **REST** software determined the best reference genes from a pool of candidates, based on the different PCR efficiencies and generated a graphical output. It provided a visual representation of variation for each gene and highlighted any potential issues or distribution skew. The smallest box-plots have the least variation and were, thus, the most stable reference genes under the experimental conditions being tested. The results identified **GAPDH**, **GUSB, HSP90, SDH and YWHAZ** as suitable reference genes.

The **qBase+** software program processed the data (see **Figure 3**), where the geNorm M results are displayed as a chart. The results show that **all the genes were below the exclusion limit of 0.2, except for PPI** (see **Figure 3A**). A geNorm V score of below 0.15 is recommended, where the results show that the optimal normalisation factor in this experiment will be generated by using the average of two reference genes in this test system (see **Figure 3B**). A multi-target bar chart for each reference gene was generated as the last output. The error bars indicate the variability, where very little difference was observed after being spiked with 25% AuNPs. However, increased amounts of 50% and 75% AuNPs produced distinct variability within the expression of the genes. To **summarise**, when comparing results obtained from the universal RNA that had been spiked with AuNPs at the reverse transcription step (part 1), all the statistical analysis programs found that the same reference genes exhibited the highest stability. These included **HSP90, SDH and YWHAZ**. In addition, stable combinations of reference genes were also identified, i.e. GUSB with HSP90.
