# Supplementary material for: The presence of residual gold nanoparticles in samples interferes with the RT-qPCR assay used for gene expression profiling
Source: J Nanobiotechnology. 2017 Oct 10;15:72. doi: 10.1186/s12951-017-0299-9 (PMC5633869; doi:10.1186/s12951-017-0299-9)
Supplement: Supplementary file 5 — Additional file 5. Manual assessment of qPCR results. [file 12951_2017_299_MOESM5_ESM.docx]

**Additional File 5: Manual assessment of qPCR results:**

Title: The presence of residual gold nanoparticles in samples interferes with the RT-qPCR assay used for gene expression profiling.

Authors: Natasha M Sanabria and Mary Gulumian

When working with ENMs, it is recommended to not solely rely on traditional qPCR software analysis programs, but to also do manual assessments, e.g. determine PCR efficiency (E) variations between treatments, as well as changes in the dissociation assay (melts) of the different products formed. All samples tested herein passed the internal quality control (QC) check, where the positive controls did not have a C_q_ greater than 30 cycles. In addition, this QC also applied when some of the reactions (specific to the 18S gene), amplified the negative/ non-template control (NTC), since it still did not have a C_q_ less than 35 cycles (see Table 4). Hence, these specific NTC results for 18S were negligible within the scope of this experimental series. The GUSB, HPRT1, HSP90 and TBP genes formed PCR products that fell within the acceptable parameters.

The CFX Manager software generated data that was used to screen for changes in the dissociation assay melt peaks of the different products formed (see Additional file 3). Due to the observed stability, these results were used to select GUSB, HPRT1 and HSP90 as candidate reference genes from the original 10 genes tested. There were no changes in the melt peaks, which indicated that the same PCR product was formed each time, irrespective of the amount of AuNPs. However, the increasing amounts of AuNPs lead to changes in the Cq. A C_q_ change of 0.2 was deemed acceptable and a C_q_ change greater than 0.5 cycles was unacceptable. This is due to the fact that a C_q_ change greater than 0.5 cycles indicates the presence of nucleotide insertions/deletions (in/dels) or even single nucleotide polymorphisms (SNPs). The increasing amounts of AuNPs lead to changes in how quickly the genes were amplified, indicated by changes in the C_q_ (see Table 5). Due to the changes in the Cq, GAPDH, GUSB, HPRT1 or SDH could possibly be suitable reference genes. However, the melt peak analysis of all the products formed did not identify any significant differences (data not shown).
