# Supplementary material for: The presence of residual gold nanoparticles in samples interferes with the RT-qPCR assay used for gene expression profiling
Source: J Nanobiotechnology. 2017 Oct 10;15:72. doi: 10.1186/s12951-017-0299-9 (PMC5633869; doi:10.1186/s12951-017-0299-9)
Supplement: Supplementary file 6 — Additional file 6. Analyses based on the point where the assay was spiked with AuNPs. [file 12951_2017_299_MOESM6_ESM.docx]

**Additional File 6: Analyses based on the point where the assay was spiked with AuNPs**

Title: The presence of residual gold nanoparticles in samples interferes with the RT-qPCR assay used for gene expression profiling.

Authors: Natasha M Sanabria and Mary Gulumian

The second part of this assessment analysed the effect of AuNPs on the DNA amplification (i.e. at the PCR step, part 2) step only. These experiments were required in order to compare the interference of AuNPs at each point in the experiment. The cDNA, obtained from the universal RNA standard, was spiked with AuNPs after reverse transcription was completed, in order to amplify the cDNA under PCR conditions with deliberate AuNP interference. This treatment would mimic those samples where DNA was isolated from AuNP-treated samples and amplified by qPCR, e.g. assessment of methylation studies, SNP and mutation analyses or genomic DNA etc. This also implies that testing only the PCR amplification step (but nothing prior), is not sufficient to verify qPCR assays for genotoxic studies related to ENM exposure assessments, where the starting material might be contaminated with residual ENMs.

The results obtained using **BestKeeper** software were, surprisingly, not as consistent as those obtained for part 1 of this study (where the reverse transcription step was spiked with AuNPs). In part 2, the greater variability between the samples was interpreted as assay interference during the amplification of the genes. In addition, it was visually observed that the addition of AuNPs reacted with the PCR enzyme cocktail and changed the colour of this super-mix to a clear solution during the preparation. Although greater variability was observed, it was still determined that GUSB and YWHAZ were the best reference genes. Similarly, the **NormFinder** software identified GUSB, HPRT1 and YWHAZ as stably expressed (see Table 7). The **REST** software recognized GUSB, HSP90 and YWHAZ as genes with stable expression. However, PPI was completely down regulated and SDH displayed a substantial degree of variance (see Figure 4). The **CFX Manager** software generated data that was used to manually assess the stability, where HPRT1, HSP90 and YWHAZ were selected as candidate reference genes. Changes to the Cq were used to assess AuNP interference and it was found that ACTB, GAPDH, HSP90, SDH and YWHAZ all fell within the acceptable limits when compared to untreated 0% AuNP samples (see Table 9). The **qBase+** software program processed the data (see Figure 5), where the geNorm M results were all below the limit, except for the PPI gene (see Figure 5A). The geNorm V results again indentified that the average of two genes would generate the optimal normalisation factor (see Figure 5B). Also, the multi-target bar chart showed that a 25% AuNP spike appeared to only affect two of the genes (see Figure 5C). In general, when comparing results obtained from the universal RNA that had been spiked with AuNPs at the amplification step (part 2), the combined results showed that only three reference genes exhibited the highest stability, i.e. GUSB, HSP90 and YWHAZ.

Further analyses deemed GAPDH and GUSB to be inappropriate as reference genes. Separation of the PCR amplicons via electrophoresis indentified the formation of multiple products for some of the replicates of GUSB, which was subsequently disqualified as a suitable reference gene. In addition, although GAPDH is a popular reference gene in many studies, it does have limitations (http://eu.idtdna.com). Firstly, GAPDH plays a role in glycolysis and as such, may result in variable expression in different tissues or disease states. Secondly, some GAPDH pseudogenes are expressed, where primers will detect the presence of both the pseudogenes and the cDNA of the active transcript. Lastly, considering that the human genome may contain up to 60 pseudogenes for GAPDH, DNase treatment may not always degrade the entire genomic DNA in which these sequences reside.

It was, thus, proposed that only YWHAZ and/or HSP90 be used as reference genes. In addition, future studies should focus on the clustering feature available in the Precision Melt Analysis^TM^ software, i.e. for high resolution melt (HRM) applications in order to identify AuNP interference of the qPCR assay. In fact, a few HRM shifts were identified by an in depth analysis of the melt profiles, where readings were taken every 0.2°C (data not shown). The same product was formed for those specific genes, where the only difference was the amount of AuNPs present in the reaction, i.e. the AuNPs appeared to influence the transcription. Thus, these genes have been identified as targets for developing a “diagnostic tool” and are currently being investigated further.
